# Progression-Mediated Changes in Mitochondrial Morphology Promotes Adaptation to Hypoxic Peritoneal Conditions in Serous Ovarian Cancer

**DOI:** 10.3389/fonc.2020.600113

**Published:** 2021-01-13

**Authors:** Joseph P. Grieco, Mitchell E. Allen, Justin B. Perry, Yao Wang, Yipei Song, Ali Rohani, Stephanie L. E. Compton, James W. Smyth, Nathan S. Swami, David A. Brown, Eva M. Schmelz

**Affiliations:** ^1^ Graduate Program in Translational Biology, Medicine, and Health, Virginia Tech, Blacksburg, VA, United States; ^2^ Department of Human Nutrition, Foods and Exercise, Virginia Tech, Blacksburg, VA, United States; ^3^ Electrical and Computer Engineering, University of Virginia, Charlottesville, VA, United States; ^4^ Fralin Biomedical Research Institute at Virginia Tech Carillion (VTC), Roanoke, VA, United States; ^5^ Department of Biological Sciences, Virginia Tech, Blacksburg, VA, United States; ^6^ Virginia Tech Carilion School of Medicine, Roanoke, VA, United States

**Keywords:** spheroids, hypoxia, fragmentation, fusion, fission, uncoupling protein, reactive oxygen species, mitophagy

## Abstract

Ovarian cancer is the deadliest gynecological cancer in women, with a survival rate of less than 30% when the cancer has spread throughout the peritoneal cavity. Aggregation of cancer cells increases their viability and metastatic potential; however, there are limited studies that correlate these functional changes to specific phenotypic alterations. In this study, we investigated changes in mitochondrial morphology and dynamics during malignant transition using our MOSE cell model for progressive serous ovarian cancer. Mitochondrial morphology was changed with increasing malignancy from a filamentous network to single, enlarged organelles due to an imbalance of mitochondrial dynamic proteins (fusion: MFN1/OPA1, fission: DRP1/FIS1). These phenotypic alterations aided the adaptation to hypoxia through the promotion of autophagy and were accompanied by changes in the mitochondrial ultrastructure, mitochondrial membrane potential, and the regulation of reactive oxygen species (ROS) levels. The tumor-initiating cells increased mitochondrial fragmentation after aggregation and exposure to hypoxia that correlated well with our previously observed reduced growth and respiration in spheroids, suggesting that these alterations promote viability in non-permissive conditions. Our identification of such mitochondrial phenotypic changes in malignancy provides a model in which to identify targets for interventions aimed at suppressing metastases.

## Introduction

Ovarian cancer is the fifth leading cause of cancer-related deaths in women, with an overall survival rate of 50%; early detection, however, increases the survival of afflicted women to 92% (1). It is a genetically and histologically heterogeneous disease and the most aggressive serous ovarian cancer is now thought to originate in the epithelial cells of the fimbriae of the fallopian tubes. Ovarian cancer is spread mostly throughout the peritoneal cavity. After exfoliation from the original tumor, the metastases are transported by the ascites to arrive at their first metastatic site, the omentum, within hours (2). Aggregation of metastases elicits a survival signal (3, 4) in a non-permissive environment that is highly hypoxic and low in nutrients; spheroid formation is also enhanced by physical stresses due to ascites build-up (5). These spheroids can remain even after cytoreductive surgery removes the original tumor and visible metastases, causing the recurrence of the disease and often patient death. The mechanisms of how aggregation increases the viability of these spheroids and their ability to adhere to secondary sites remain unclear.

Mitochondria are important signaling organelles that regulate bioenergetics and biosynthesis and are primary responders of stress sensing to aid in the adaptation to a changing microenvironment (2). Mitochondria are continuously undergoing fusion and fission to share organelle contents, allow for mitochondrial networking, enable quality control by regulating mitophagy and apoptosis, and the control of cell cycle progression (6). Fusion is regulated by mitofusin 1 (MFN1) and optic atrophy 1 (OPA1) while fission 1 (FIS1) and dynamin-related protein 1 (DRP1) regulate fission. Cells undergo controlled mitochondrial fragmentation during mitosis to permit equal distribution of mitochondria between daughter cells (7); this is reversible as cells in G1/S phase exhibit mostly filamentous mitochondria (8). Compromised mitochondria can fuse with healthy organelles in order to receive cellular components necessary to compensate for damage (9, 10). Additionally, in case of drastic changes in mitochondrial membrane potential, the E3 ligase Parkin tags such mitochondria for degradation through selective autophagy or mitophagy. Fragmentation of mitochondria in cardiac muscle is associated with cell death and cardiac dysfunction (11, 12). In contrast, mitochondrial fragmentation due to an increase in DRP1 expression in the lamellipodia precedes migration in metastatic breast cancer (13). Mitochondrial fragmentation has also been associated with neurodegenerative diseases, an aberrant lipid and glucose homeostasis in the liver, and impaired differentiation of stem cells (14, 15). Thus, the outcome of mitochondrial fragmentation appears to be tissue specific and its role in cancer cells is yet to be clearly elucidated.

By constantly adapting their bioenergetic processes to fulfill cellular energy demand, mitochondria produce a large amount of ROS which can be damaging to the organelles and the cell. However, balanced levels of intracellular ROS can be beneficial in activation of proliferative signaling pathways, including mitogen-activated protein kinase and phosphoinositide 3-kinase (MAPK and PI3K), especially through balancing redox potential (16–20). ROS have been previously shown to stabilize hypoxia inducible factor 1α which activates adenosine monophosphate-activated protein kinase to enhance cellular energetic homeostasis in several tumors including human prostate cancers (21). Also, ROS have been shown to activate uncoupling proteins (UCP) 2 and 3 to limit proton leaks and oxidative stress (22, 23), linking ROS to alterations in cellular metabolism. Further, oxidative stress reduces both fusion and fission events in skeletal muscle myoblasts and causes mitochondrial fragmentation and subsequent apoptosis (24). This process is highly reliant on the mitochondrial membrane potential and the ability of the cells to remove oxidative stress, as well as release cytochrome c, which impacts mitochondrial dynamics. Upon short-term exposure to H_2_O_2_, myoblasts induce mitochondrial fragmentation through translocation of DRP1 to the outer mitochondrial membrane and promote fission (24). Cancer cells have been shown to have imbalanced expression of these proteins, promoting cell survival and preventing apoptosis. For example, overexpression of OPA1 can be induced through hypoxic stress leading to a thickening of the inner mitochondrial membrane that prevents cytochrome c release (10, 13). Thus, oxidative stress and mitochondrial dynamics are critically connected to the fate of the cells.

Previous studies in our lab have shown that ovarian cancer cells acquire a more glycolytic and metabolically flexible phenotype during progression (25, 26). Further, aggregation and hypoxia reduced cellular respiration that corresponds with a significantly reduced proliferation rate (27). Here we investigated how mitochondrial morphology and dynamics are altered during ovarian cancer progression and upon aggregation to gain insight into how changes in mitochondrial morphology contributes to the survival of aggregates in the non-permissive environment of the peritoneal cavity during metastasis. Utilizing our previously described mouse ovarian surface epithelial (MOSE) model for serous ovarian cancer (28–33), we identified the morphological and functional changes that occur during the progression from benign (MOSE-E), to slow (MOSE-L) and fast-developing disease (MOSE-L_TIC_ *_v_*), and in response to aggregation and hypoxia. These changes may contribute to the increased survival and metastatic potential of the most aggressive MOSE-L_TIC_ *_v_* and could therefore represent a novel target for treatment strategies aimed at suppressing ovarian cancer metastasis.

## Materials and Methods

### Cell Culture

The MOSE cell lines represented benign (MOSE-E), slow-developing (MOSE-L), and aggressive (MOSE-L_TIC_ *_v_*) ovarian cancer cells generated from C57BL/6 mice have been extensively characterized previously (28–33). These cells express fallopian tube markers (29) and are therefore a model for the highly aggressive serous ovarian cancer. All cells were grown in high glucose DMEM (Sigma Aldrich) supplemented with 4% fetal bovine serum (Atlanta Biological), 3.7g/l sodium bicarbonate, 10 ml/l of penicillin-streptomycin solution at 37°C with 5% CO2 under normoxic (21% O_2_) or hypoxic conditions (1–2% O_2_). Human TERT-immortalized benign and malignant fallopian tube (FNE1, FNLE1) and benign ovarian epithelial cells (OCE1) were obtained from the Live Tumor Culture Core at the University of Miami Sylvester Comprehensive Cancer Center and cultured in Primaria tissue culture flasks (Becton Dickinson) with FOMI media supplemented with 25ng/ml cholera toxin as described (34). SKOV3 (ascites-derived ovarian serous carcinoma) were from ATCC and cultured in DMEM supplemented with 10% fetal bovine serum. Spheroids from transformed cells were generated by seeding cells onto ultra-low adherence plates (Corning) for 48 h. Benign cells do not form viable spheroids.

### Immunofluorescence Staining

Cells were grown to 80% confluency and trypsinized before seeding. Single cells were plated at a density of 2 x 10^4^ onto individual 12mm^2^ glass coverslips and incubated for 48 h to allow for cells to adhere and begin to grow. Coverslips were stained with 50nM MitoTracker Red CMXRos (Molecular Probes) for 15 min at 37°C, fixed with paraformaldehyde with 0.5% triton-X 100 and quenched with 50mM glycine. To study mitophagy, MOSE cells were fixed in methanol and immunostained with anti-LC3B (Cell Signaling) and with a FITC-conjugated rabbit secondary antibody (Molecular Probes). Coverslips were mounted onto glass slides using Prolong gold antifade mounting medium with DAPI (Molecular Probes) to allow for visualization of the nuclei. Images were captured with a Nikon 80/fluorescent microscope equipped with DAPI, FITC, and TRITC filters using the NIS elements BR 3.0 software and were processed using Adobe Photoshop CS6. DAPI and TRITC images were merged to display localization and spread of mitochondria from the nucleus.

### Mitochondrial Characterization of Adherent Cells

The MyMia algorithm measures 25 different morphological features in each cell including both cell level measurement and branch level measurements as described previously (35). In order to separate mitochondrial features from those of the nucleus, we used color channeling to identify the differentially stained features. This structure was then used to identify branches (individual mitochondrion) and branch points (mitochondrial joints) using previously described formulas (35). Each branch is used as a mask to perform measurements such as length, width, area of pixels on each individual mitochondrion. Four different parameters were quantified and observed for about 50 images per cell type; number of branches (subtracting all the intersection from the skeleton of mitochondria network and labeling all the remaining branches), mean branch length (observed length of quantified branches per image), distance from the nucleus (mean distance between centroid of the branches and the centroid of the nuclei), and circularity (determination of how many of the objects are circular.

### Transmission Electron Microscopy

MOSE cells were grown to 80% confluency, trypsinized, and fixed with Karnovsky’s fixative (4g PFA/50ml, 10ml 50% glutaraldehyde in 100ml 0.2M PBS) overnight. Cells were washed 3x with 0.1M PBS for 15 min. After treatment with 1% OsO_4_ in 0.1M PBS for 1 h, cells were washed 2x for 10 min with PBS. Samples were then dehydrated with increasing concentrations of graded ethanol as follows: 15%, 30%, 50%, 70%, 95%, 100% for 15 min each. Dehydration was completed using propylene oxide. After incubation with a 50:50 propylene oxide: Poly/Bed812 (Polysciences Inc.) solution for 24 h, the samples were embedded in 100% Poly/Bed 812 in flat embedding molds and placed in a 60°C oven for 48 h. Mitochondrial ultrastructure was visualized at 60 and 80x magnification on a JEOL JEM 1400 scope. Images were assembled in Adobe Photoshop™.

### Western Blotting

Adherent cells and spheroids were cultured for 48 h under normoxic and hypoxic conditions. Cells were lysed in radioimmunoprecipitation buffer supplemented with protease and phosphatase inhibitors. Protein concentrations in the lysates were quantified using the Pierce Bicinchoninic acid assay (Thermo Fisher Scientific). Equal protein concentrations were loaded in a 4% stacking and 10% SDS separating gel. Proteins were transferred onto a PVDF membrane (Bio-Rad) and blocked with 5% milk in TBST. Primary antibodies were used against DRP1, OPA1 (Novus Bio), MFN1, FIS1 (Protein Tech), UCP2 (Santa Cruz), UCP3 (ThermoFisher Scientific), TOMM 20 (Millipore), and superoxide dismutase 2 (SOD2) antibody (Cell Signaling). Proteins were normalized using the ribosomal protein L19 (L19) or to total protein normalization substrate (Thermo Fisher Scientific). Blots were subsequently probed with the appropriate HRP-conjugated mouse and rabbit or IRDye 680/800cw (Licor) secondary antibodies Proteins were visualized using chemiluminescence Pico ECL (ThermoFisher Scientific) solution with an exposure of 15–30s using the transilluminator from Bio-Rad or the Licor Odyssey Clx imaging system. Proteins quantified using ImageJ software. Data presented as mean ± SEM from at least three biological replicates.

### Imaging of Mitochondrial Membrane Potential on Ovarian Cancer Cells

Cells were plated at 10,000 cells per well in black 96-well microplates with glass bottom (Corning) in normoxic and hypoxic media conditions. Prior to imaging, cells were treated with 10nM tetramethyl rhodamine (TMRM) for 30 min, protected from light in a 37°C/5% CO2 incubator. Fluorescent images of mitochondrial membrane potential were obtained at 542.0 nm (27.0 nm bandpass) excitation and 587nm (45nm bandpass) emission on a GE INCell Analyzer 2200 (GE Healthcare). Sequential qualitative images were taken in 10 fields of view for each channel in each well at 37°C. Images were analyzed using GE’s InCarta software version 1.6. Multiple parameters were collected from each plate by creating custom “masks” that captured the TMRM fluorescence signal, allowing for subsequent quantification. Data are expressed as intensity (the mean pixel value under the mask)—background (mean pixel value for the local background) in Arbitrary Units (A.U.) ± SEM.

### Reactive Oxygen Species Production Assay

MOSE cells were seeded at 2.5 x 10^4^ cells/well and incubated in normoxic and hypoxic conditions for 24 h in flat, glass bottom 96-well microplates. The cells were washed with 0.25mM sodium phosphate solution (pH to 7.4) warmed to 37°C 30 min prior to the experiment. Cells were stained with 25µM 2’7’-dichlorofluorescin diacetate (DCFDA) (Abcam) in Krebs-ringer phosphate buffer for 45 min at 37°C to measure H2O2 levels in live cells as previously described (36, 37). ROS production was quantified using a TECAN plate reader measuring the excitation fluorescence (set at 485ex/535em). MOSE-E cells treated with 1mM H2O2 served as positive control. To measure extracellular ROS production, we used Amplex Red (ThermoFisher Scientific) at 50µM in combination with 10U/ml horseradish peroxidase, according to the manufacturer’s instructions; fluorescence was read at 571ex/585em. ROS production was normalized by the protein concentration.

### Confocal Microscopy of Mitochondria in Spheroids

Confocal imaging of spheroid mitochondria was performed as described previously (34). Briefly, spheroids grown in normoxia or hypoxia were treated with 100nM of TMRM (Molecular Probes) for 30 min to ensure penetration throughout the spheroid. After incubation, the samples were washed with PBS and plated on glass coverslips in DMEM media for imaging using a confocal Leica SP8 DMi8 microscope, at excitation/emission wavelengths of 552/576nm, respectively. Images were processed using the Leica LASX software (512x512 pixel resolution) and assembled in Adobe Photoshop™.

### Super-Resolution Microscopy to Quantify Mitochondrial Fragmentation in Spheroids

Cultivated MOSE-L and MOSE-L_TIC_ *_v_* spheroids were treated with 25nM MTDR. Spheroids were then placed onto glass bottom 35mm petri dishes (Cellvis) to adhere for 2 h prior to fixation with 4% PFA. Stochastic Optical Reconstruction Microscopy (STORM) imaging was conducted on a Vutara SR 350 system (Bruker) using 50mM Tris-HCl, 10mM NaCl, 10% (wt/vol) glucose buffer containing 20mM mercaptoethylamine, 1% (vol/vol) 2-mercaptoethanol, 168 active units/ml glucose oxidase, and 1,404 active units/ml catalase. Five thousand frames were acquired for each dataset and cluster analysis of assigned localizations performed using Vutara SRX software. Mitochondrial fragmentation was assessed and compared between different cell types and media conditions using the image-based cluster analysis module within the Vutara SRX software. Specifically, individual mitochondria were identified as clusters through implementation of the following parameters: density map resolution: 100px/µm, density axial resolution 5 slices/µm, minimal cluster area: 0µm^3^, maximum cluster area: 10,000µm^3^, minimum particle count: 250, particle size: 100nm, opacity: 0.30, accumulation threshold: 0.0l, half alpha shape radius: 0.10µm. STORM localization datasets were acquired for 9 separate sections of each aggregate to quantify and compare the cluster area and particle count between different cell types and media conditions. The surface area and particle count were also assessed for the top, middle, and bottom portions of each spheroid.

### Statistical Analyses

Data are presented as mean ± SEM. Comparisons between different cell types and oxygen content were analyzed using a one-way ANOVA followed by Tukey’s multiple comparison test. Spheroid comparisons between MOSE-L and MOSE-L_TIC_ *_v_* were analyzed using a student’s two-tailed *t-test.* Results were considered significant at p<0.05.

## Results

### Cancer Progression and Hypoxic Conditions Promote Changes in Mitochondrial Morphology in Mouse and Human Cell Lines

**Figure 1:**
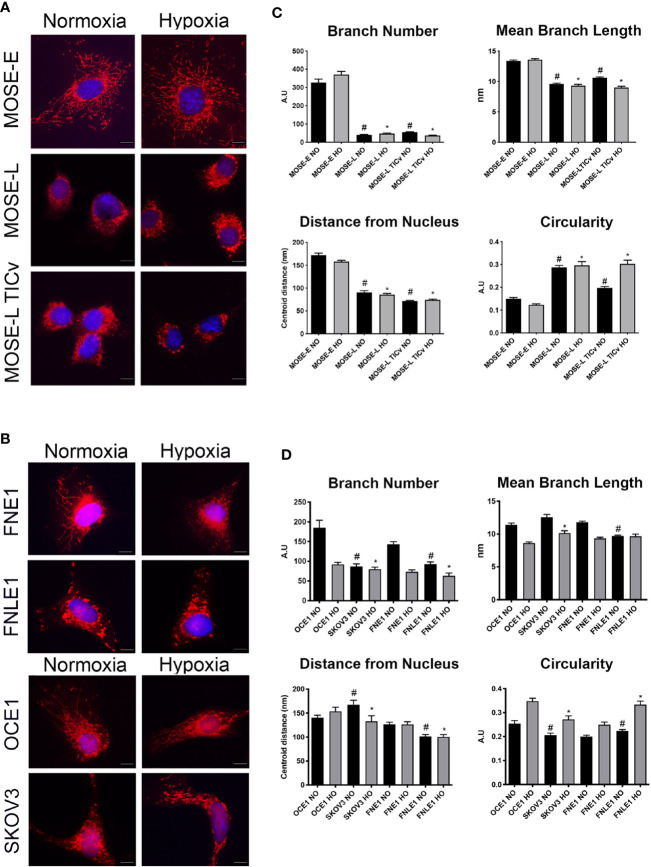
Increasing malignancy and hypoxia promote a fragmented mitochondrial phenotype in mouse and human cell lines. Adherent mouse benign MOSE-E, malignant MOSE-L and MOSE-L_TIC_ *_v_* cells **(A)**, human benign (FNE1), and malignant fallopian tube (FNLE1) and benign (OCE1) and malignant ovarian cells (SKOV3) cells **(B)** were stained with MitoTracker CMXRos and DAPI. Scale bar set at 20µm. The MyMia algorithm was used to characterize and quantify mitochondrial morphology (branch number, mean branch length, localization and circularity **(C, D).** Comparison to benign cells ^#^p < 0.05 in normoxic (NO), *p < 0.05: in hypoxic (HO) conditions.

The MOSE model for progressive serous ovarian cancer (29–33) was used to initiate investigations of differential mitochondrial morphology changes during tumorigenesis in culture conditions that begin to more accurately reflect conditions in the peritoneal cavity. **Figure 1A** illustrates qualitatively how the elongation of the mitochondria was severely reduced during cancer progression from a filamentous phenotype observed in the MOSE-E cells, to mitochondria appear aggregated and localized mostly perinuclearly in the transformed cells with no apparent difference between the MOSE-L and MOSE-L_TIC_ *_v_*. Mitochondrial morphology changes were compared to benign (FNE1) and malignant (FNLE1) human fallopian tube cells and benign (OCE1) and malignant (SKOV3) human ovarian cells using the same culture conditions (**Figure 1B**). The human cells showed a similar loss of filamentous mitochondria after transformation as seen in MOSE cells, indicating that such changes are conserved between mice and humans and occur in both ovarian and fallopian tube epithelia. Hypoxia only minimally affected the mitochondrial morphology in both the mouse and human cells (**Figures 1A, B**, right panels).

In order to quantify the alterations in mitochondrial morphology, at least 40 images of each cell type were analyzed for localization of the mitochondria, their number of branches, mean branch length, and circularity as indicators of normal morphology and distribution. Both benign mouse and human cells had a significantly higher number of mitochondrial branches with a larger mean branch length in comparison to their malignant counterparts (both p<0.05) (**Figures 1C, D**). While hypoxia did not affect these parameters in the mouse cells, the benign human cells showed a significantly reduced mitochondrial branch count and area under hypoxic conditions (p<0.001) (**Figures 1C, D**). The mitochondria were distributed throughout the cells in all benign cell types but were mostly localized in the perinuclear region in the transformed cells as indicated by the significantly smaller average distance of mitochondria to the nucleus. Mitochondrial shape was significantly more circular in the transformed cells in both the mouse and human cells except the SKOV3 line; hypoxia significantly increased circularity in both mouse and human cells except in the MOSE-L and FNE1 cells (p<0.001 for all cells, p<0.01 for SKOV3 cells). These results indicate that transformation in both human and mouse ovarian and fallopian tube epithelial cells is accompanied by morphological changes and relocation of more circular mitochondria to the perinuclear region of the cells; these morphological changes are very little affected by hypoxia. While there were some differences in the metrics between the human and the mouse cells, these may be the result of the multiple transfections of the human cell lines to achieve immortalization and transformation *versus* the spontaneous processes in the MOSE cells; transfection reagents and empty vector backbones have been shown to affect the expression of off-targets genes (38). Further, the rounded morphology of the human cells may have contributed to the selection of cells that allowed for mitochondrial imaging. Overall, our results show the same changes in mitochondrial shape, size, and localization in both mouse and human cells. Thus, we continued our studies with the better characterized MOSE cells having validated their relevance to human disease.

### Ultrastructure of Mitochondria During Malignant Transition

**Figure 2:**
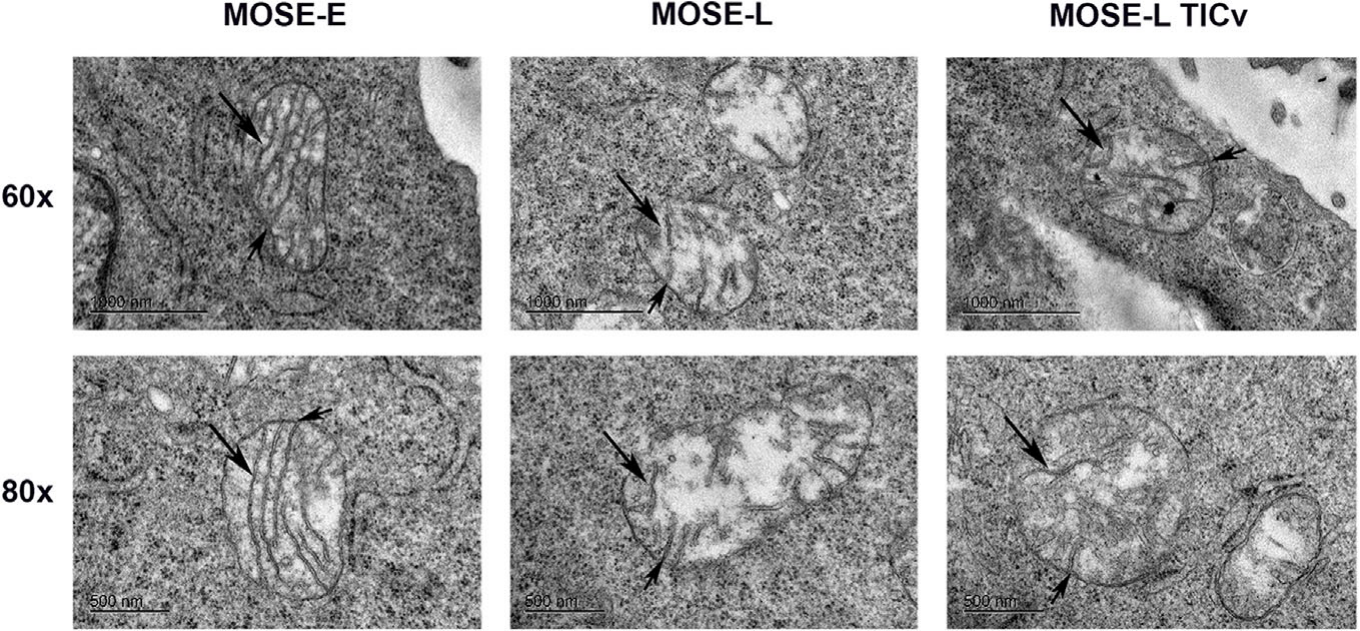
Changes in the ultrastructure of mitochondria during malignant progression. Mitochondria in benign MOSE-E and malignant MOSE-L and MOSE-L_TIC_ *_v_* cells were observed by TEM. Long arrow: mitochondrial cristae; short arrow: cristae junctions.

We next used transmission electron microscopy (TEM) to determine if the ultrastructure of these organelles changes during malignant progression, and if the large round mitochondria localized in the perinuclear region of the cancer cells are the result of aberrant fusion. As shown in **Figure 2**, the MOSE-E show well developed cristae and cristae junctions to the inner membrane. This organization is lost during malignant progression and there were fewer and disorganized cristae in the cancer cells. These cancer mitochondria were enlarged single organelles rather than fused aggregates. While the TEM images do not allow for the visualization of connections, as apparent in **Figure 1** these organelles appear not to be connected in a mitochondrial network. However, even the benign MOSE-E show some swelling, suggesting that either the immortalization process or cell culture conditions impact mitochondrial morphology of cell lines. These results indicate that with increasing malignancy, mitochondria have less structural integrity; this does not impact their viability since the cancer cells grow faster than the MOSE-E (28) but may contribute to their altered metabolic phenotype (25, 26).

### Hypoxia Increases Autophagy in Ovarian Cancer Cells

**Figure 3:**
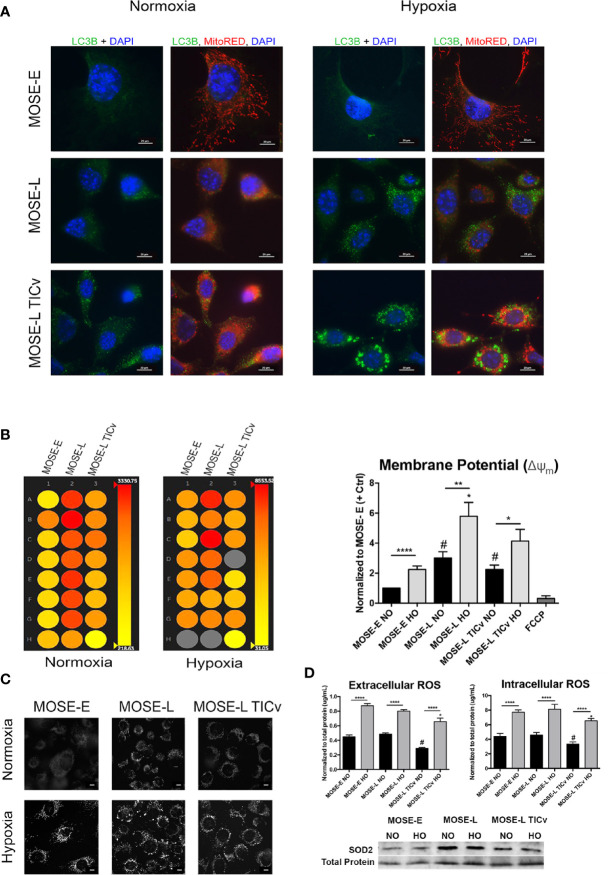
Disease-stage dependent responses to hypoxia. **(A)** Determination of autophagy as apparent by LC3B expression in MOSE cells of increasing metastatic potential grown in normoxic and hypoxic conditions. **(B)** Representative heat map (left) and quantitation (right) of mitochondrial membrane potential of MOSE cells stained with tetramethyl rhodamine (TMRM) as determined by INcell 2200 analysis. **(C)** Representative images of TMRM stained adherent cells (NO/HO). Scale bar set at 20µm. (**D**, top) Quantitation of intracellular and extracellular ROS production normalized to total protein content. *p < 0.05, **p < 0.01 ****p < 0.001; ^#^p < 0.05 different from the benign MOSE-E cells. (**D**, bottom) SOD2 protein expression in adherent MOSE cells (NO/HO) normalized to total protein normalization substrate.

To determine if the morphologic changes during cancer progression are associated with autophagy or mitophagy as a measure of mitochondrial quality control, we used indirect immunofluorescence staining for LC3B to visualize autophagosomes. As shown in **Figure 3A**, there were no autophagosomes detectable in normoxic benign cells but low levels of LC3B-labeled autophagosomes were observed in transformed cells. This was drastically increased under hypoxic conditions (**Figure 3A**, right panels). The MOSE-E cells, however, do not alter levels of autophagy during hypoxia suggesting that these cells do not respond in the same manner to lower oxygen levels in order to enhance survival.

Changes in mitochondrial membrane potential and phenotype precedes mitophagy (39). Therefore, we next investigated how the mitochondrial membrane potential is altered during cancer progression to distinguish between autophagy and mitophagy using qualitative and quantitative TMRM detection. The transformed cells have a significantly higher mitochondrial membrane potential than the benign MOSE-E cells despite their altered morphology (p<0.0001 for MOSE-L and p<0.05 MOSE-L_TIC_ *_v_* respectively) (**Figures 3B, C**). Furthermore, hypoxia increased the membrane potential in all cells (p<0.0001 for MOSE-E, p<0.01 for MOSE-L, and p<0.05 for MOSE-L_TIC_ *_v_*). Interestingly, this increase in membrane potential coincides with the increased levels of autophagy; while a collapsed mitochondrial membrane potential identifies damaged mitochondria and often induces mitophagy, our data indicate that in the malignant MOSE cells the membrane potential is increased and enhances autophagy to promote cell survival as has been observed in breast cancer cells (40).

The shift in the balance of general ROS production and elimination has been suggested to increase ROS steady-state levels in cancer cells that is counter-balanced by a higher antioxidant capacity (41). Since ROS production is often associated with high mitochondrial membrane potential and induction of autophagy, we measured intracellular and extracellular ROS production. There was no detectable difference in intra- or extracellular ROS levels between the MOSE-E cells and the MOSE-L; however, the highly tumorigenic MOSE-L_TIC_ *_v_* showed significantly lower ROS levels (P<0.05 *vs*. MOSE-E and MOSE-L). Hypoxia significantly increased ROS levels in all three cell types (P<0.0001) with the MOSE-L_TIC_ *_v_* still having the lowest amounts (**Figure 3D**). To determine if hypoxic ROS accumulation is indicative of limited scavenging capacity, protein expression of SOD2 was observed by Western blotting. Neither cell line increases their SOD2 expression in hypoxia, thus explaining the increased ROS accumulation. The MOSE-L_TICv_, cells, however, show consistent levels of SOD2. This indicates that either the MOSE-L_TIC_ *_v_* cells reduce ROS production or the accumulated ROS were scavenged in an alternative way to limit redox potential and prevent apoptosis. These results suggest that in contrast to the MOSE-L cells, the most aggressive MOSE-L_TIC_ *_v_* may be able to control their ROS content through selective removal of damaged mitochondria *via* autophagic degradation to support their viability in the hypoxic peritoneal cavity.

### Aggregation of Tumorigenic Mouse Ovarian Surface Epithelial Cells Causes Mitochondrial Fragmentation

**Figure 4:**
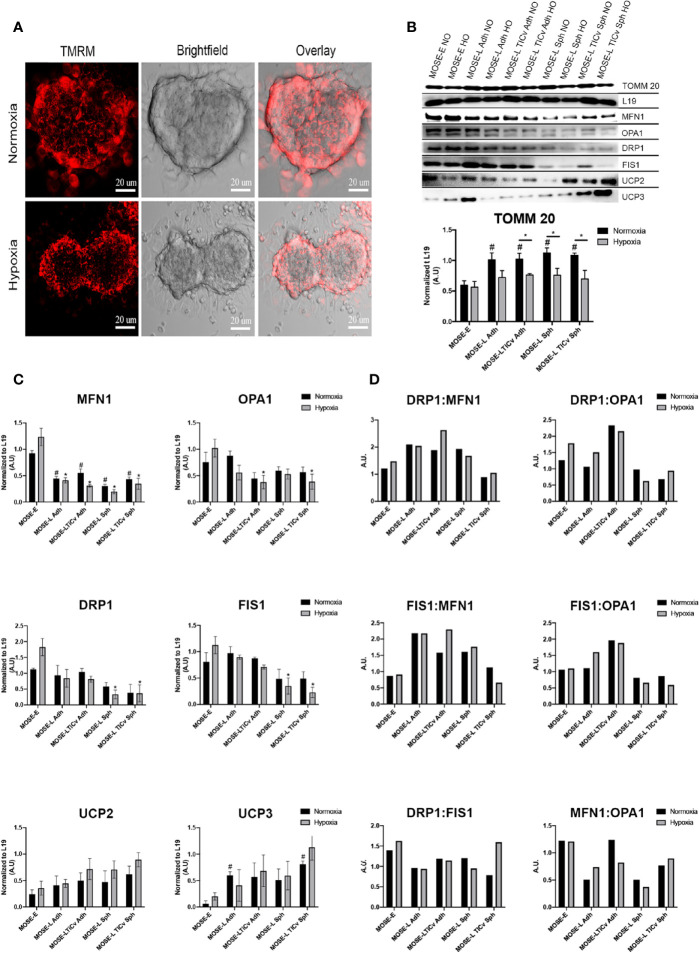
Changes in mitochondrial morphology during aggregation. **(A)** Mitochondrial morphology in tetramethyl rhodamine (TMRM) stained MOSE-L_TIC_ *_v_* cells grown as spheroids. Scale bar set at 20µm. **(B, C)** MOSE cells grown as adherent monolayers (Adh, lanes 1–6) or spheroids (Sph, lanes 7–10) were exposed to NO/HO conditions and analyzed *via* western blot. Mitochondrial protein TOMM 20, fusion [MFN1, OPA1 (top band)], fission (DRP1, FIS1) and uncoupling (UCP2, UCP3) proteins were normalized to the ribosomal protein 19 (L19). Displayed blots have been cropped using Adobe Photoshop. Each protein quantified at n ≥ 3. Exposure set at 15–30s for each protein. *p < 0.05; # Different from the benign MOSE-E at p < 0.05. **(D)** Ratios of fission and fusion proteins.

Aggregation is a survival signal for cancer cells and increases their metastatic potential (3, 4). To investigate how aggregation affects mitochondrial morphology, we generated spheroids from MOSE-L_TIC_ *_v_* cells since these cells are most responsive to hypoxia (**Figure 2**) and nutrient deprivation (25). The cells were stained with the mitochondria-specific dye, TMRM that permeates throughout the spheroid (**Figure 4A** and **Supplementary Video 1**). Using confocal microscopy, we show that the mitochondria are fragmented in the spheroid core; the higher fluorescence levels in the outer layer of the spheroids indicate that mitochondrial fragmentation is less frequent in areas where mitotic features can be observed (42). The fragmentation of mitochondria in the spheroid core was further enhanced by hypoxia, suggesting an adaptation of the mitochondrial morphology to differential oxygen levels.

To investigate whether the altered mitochondrial morphology was the result of changes in the expression of proteins regulating fusion and fission dynamics, we determined the expression levels of MFN1 and OPA1 (fusion) and FIS1 and DRP1 (fission). As evident by the levels of TOMM 20 (translocase of outer mitochondrial membrane 20), there was a significant increase in total mitochondrial protein content during malignant transition which was significantly reduced under hypoxic conditions in the transformed but not in the benign cells. As shown in **Figures 4B, C**, all observed fusion and fission proteins were lowered in the MOSE-L and MOSE-L_TIC_ *_v_* cells in comparison to the MOSE-E cells in hypoxic conditions (normalized to L19) but only MFN1 and DRP1 proteins were increased in the MOSE-E cells in normoxic conditions (0<0.05). Hypoxia had a nominal effect on protein expression in the cancer cells but significantly increased both fusion and fission proteins (MFN1 and DRP1, respectively) in the benign MOSE-E cells (p<0.05). However, with increasing malignancy, the ratio of fission to fusion proteins increased in the adherent cells, specifically the DRP1:MFN1 ratio (**Figure 4D**) which could contribute to their increasing fragmented phenotype illustrated in **Figure 1**. Interestingly, aggregation did not exacerbate these ratios and in the most aggressive MOSE-L_TIC_ *_v v_* spheroids despite the changes in mitochondrial morphology, the ratios more closely mimicked those observed in the benign MOSE-E. Fission and fusion proteins were significantly lower after aggregation of MOSE-L (MFN1, DRP1, and FIS1: p<0.05 in hypoxia, MFN1: p<0.05 in normoxia), and MOSE-L_TIC_ *_v_* (MFN1, OPA1, DRP1, and FIS1: p<0.05 in hypoxia, MFN1: p<0.05 in normoxia. However, there was an increase in OPA1 proteins in comparison to DRP1 and FIS1 in the MOSE-L and MOSE-L_TIC_ *_v_* spheroids.

Uncoupling proteins regulate cellular metabolism and ROS production (43) and are overexpressed in several cancers (44). UCP2 and UCP3 protein expression increased during ovarian cancer progression and after aggregation in comparison to the MOSE-E with a significant increase in UCP3 for the adherent MOSE-L and MOSE-L_TIC_ *_v_* spheroids (p<0.05). Both proteins were further increased in hypoxic conditions especially in the MOSE-L_TIC_ *_v_* aggregates. Taken together, these data indicate that a shift in the balance of regulatory protein expression rather than an increase in fission proteins contributes to mitochondrial fragmentation in aggressive cancers. 

### Super Resolution Analyses of Spheroid Mitochondria Morphology Confirm Enhanced Fragmentation With Increasing Malignancy

**Figure 5:**
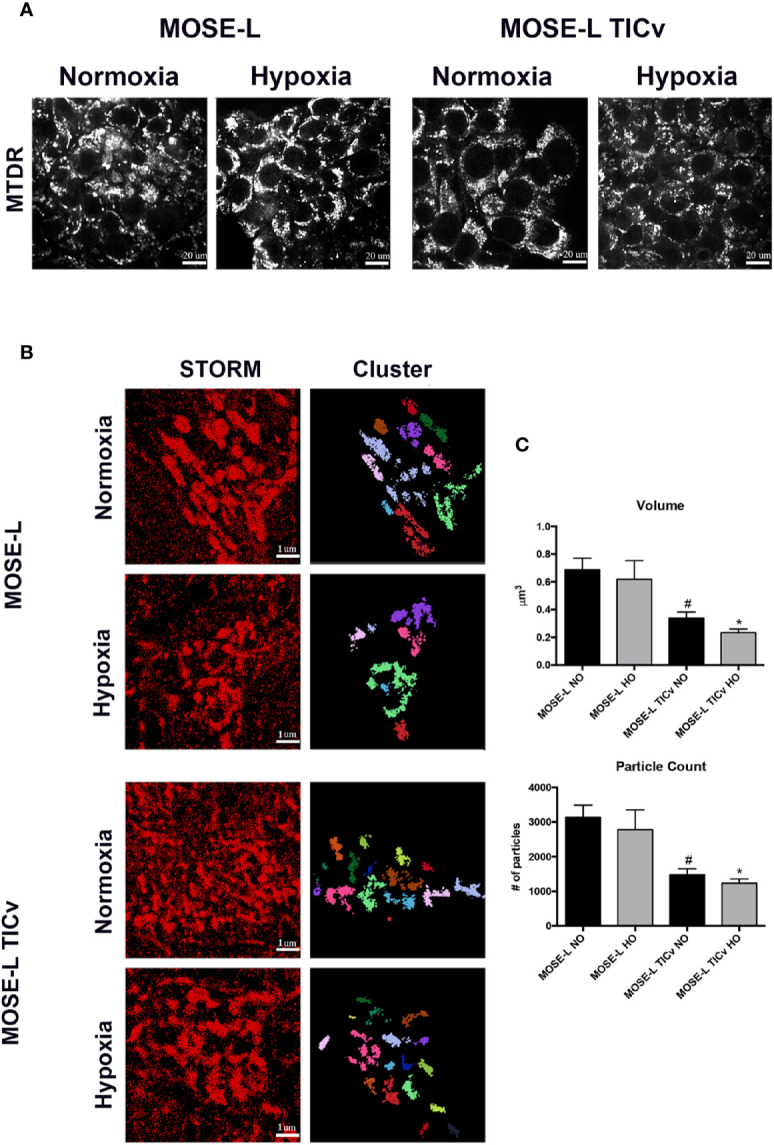
Mitochondrial fragmentation is enhanced during aggregation. **(A)** Representative images of mitochondria in MOSE-L and MOSE-L_TIC_ *_v_* spheroids obtained by confocal fluorescent microscopy (scale bar set at 20µm) indicating MitoTracker deep red (MTDR) staining was retained during aggregation and after 48 h incubation prior to MeOH fixation. **(B)** STORM imaging (scale bar set at 1µm.) with cluster representations of assigned localizations (right) from super resolution images. **(C)** Mitochondrial fragmentation was quantified using the image-based cluster analysis module with the SRX software. ^#^p < 0.05 different from MOSE-L in normoxia, *p < 0.05 different from MOSE-L in hypoxia.

To quantify the fragmented mitochondrial phenotype in spheroids, MOSE-L and MOSE-L_TIC_ *_v_* cells were incubated with MitoTracker deep red (MTDR) prior to aggregation to label mitochondria in all cells. Representative confocal images were taken to confirm MTDR staining prolonged the 48hr incubation prior to fixation (**Figure 5A**). Stochastic optical reconstruction microscopy (STORM) was employed to identify mitochondrial morphology at nanoscale resolution. Individual clusters were selected in the super-resolution localizations, identifying individual mitochondria within the spheroid core (**Figure 5B**). Volume and particle count were quantified using the Vutara SRX software cluster analysis to determine fragmentation levels. As shown in **Figure 5C**, mitochondrial volume and particle counts are significantly lower in MOSE-L_TIC_ *_v_* after aggregation than in MOSE-L spheroids (p<0.01 in normoxic and p<0.001 in hypoxic conditions); while hypoxia reduced both parameters even further this was not statistically significant. These data suggest that the more aggressive MOSE-L_TIC_ *_v_* phenotype is associated with the ability to respond to aggregation and hypoxia with mitochondrial fragmentation.

### Regional Mitochondrial Quantification Shows Increased Fragmentation at the Core of Ovarian Cancer Spheroids

**Figure 6:**
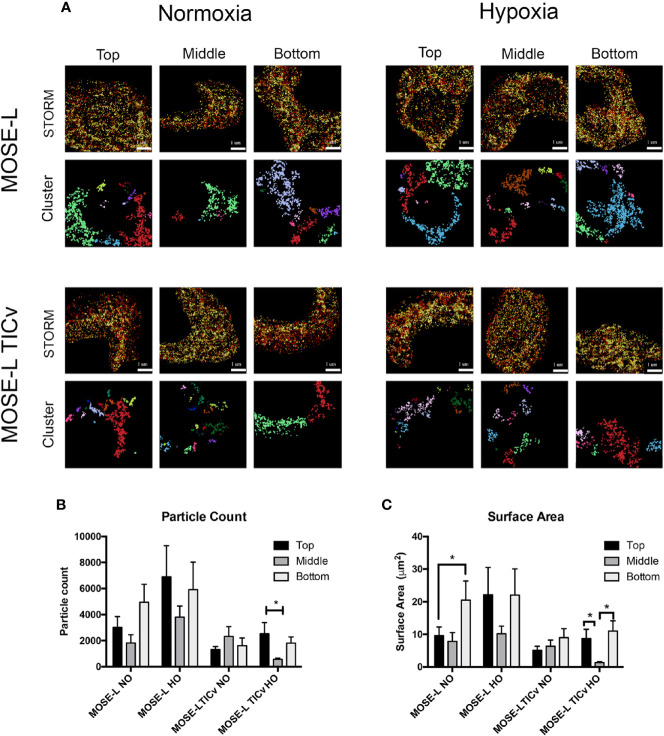
Mitochondrial fragmentation is regionally altered in ovarian cancer spheroids. **(A)** Regional super resolution stochastic optical reconstruction microscopy (STORM) imaging of MOSE-L and MOSE-L_TIC_ *_v_* spheroids and cluster representations of assigned localizations. Localizations colored by depth (darker indicates increasing spheroid depth) scale bar set at 1µm. **(B, C)** Quantitation of mitochondrial fragmentation and morphological structure using the image-based cluster analysis module with the Vutara SRX software system for cluster particle count and surface area. *p < 0.05.

To determine how mitochondrial morphology differs at specific regions of the cultured spheroids, and if there is a difference between the slow and fast-developing disease, STORM localization was used to image mitochondria in the top, middle, and bottom portions of MOSE-L and MOSE-L_TIC_ *_v_* spheroids in normoxic and hypoxic conditions. Individual mitochondria were identified as separate clusters using the same parameters as described above. As shown in **Figures 6A–C**, while there were fewer and smaller mitochondria particles in the core of MOSE-L spheroids than on the outer layer, data were variable and, thus, not statistically significant. This did not change in hypoxic conditions. However, MOSE-L_TIC_ *_v_* spheroids exhibited a lower number of mitochondrial particles with no detectable differences between the core and the top or bottom layers but a significant reduction of both particle count and surface area in hypoxic conditions (also significantly lower than MOSE-L core area, p<0.05). These results suggest that the more aggressive MOSE-L_TIC_ *_v_* spheroids reduce mitochondrial number and size in response to aggregation and reduced oxygen availability that may contribute to the more flexible metabolic phenotype we have described previously (25, 26).

## Discussion

Ovarian cancer cells exfoliate from the original tumor and disseminate throughout the peritoneal cavity. Aggregation enhances their survival; however, the mechanisms of this protection from non-permissive (hypoxic, low nutrient, and serum starved) conditions are not known. Here we utilized our syngeneic MOSE model that represents different stages of the disease with the same cell origin found also in the human disease. This model shows the same changes in functional categories as described in the human disease (29) and eliminates the inter-individual differences that can affect the findings and data interpretation. We investigated quantitative and qualitative changes in mitochondrial morphology and localization during cancer progression and as a result of aggregation and in more physiologically relevant culture conditions. We show for the first time that progression changed the mitochondrial morphology from a filamentous network to fewer large single organelles with a greater degree of circularity that are often localized around the nucleus (**Figure 1**). The cancer mitochondrial phenotype was characterized by a swollen appearance with disorganized cristae (**Figure 2**), increased mitochondrial membrane potential but not elevated ROS production (**Figure 3**). Aggregation increased the fragmentation of the mitochondria in core portions of spheroids, especially under hypoxic conditions. This was not due to an increase in the expression of proteins that regulate fission but rather an imbalance of fission to fusion protein ratio since the expression of all investigated regulatory proteins was significantly decreased in cancer cells and after aggregation (**Figure 4**). In contrast to reports of detrimental mitochondrial fragmentation in other tissues, the changes observed here did not induce cell death in the ovarian cancer cells; despite their changed morphology, the cancer mitochondria are functional—albeit more glycolytic—and support rapid growth (25, 26, 28). Cellular metabolism was identified as one of the functional categories altered during MOSE cell progression (29), and recent studies in our lab have shown that aggregation and hypoxia reduce the growth rate and cellular respiration of MOSE spheroids Compton et al. (27), suggesting that mitochondrial fragmentation may represent an adaptation to external conditions and contribute to the survival of the spheroids in a non-permissive environment.

TEM imaging in **Figure 2** showed that the mitochondria in the cancer cells were larger and appeared swollen. It is thought that the swelling is the result of fluid influx that expands the mitochondrial matrix; however, it is unclear how this affects mitochondrial functions since an increase, decrease, or no change in mitochondrial membrane potential have all been reported to promote swelling (45). Mild swelling in response to an osmotic imbalance has been shown to increase mitochondrial function and metabolism (46) while non-physiological swelling can rupture the outer mitochondrial membrane and induce cell death (47). Thus, the impact of mitochondrial swelling may be tissue specific and dependent on the degree of enlargement. Enlarged but functional mitochondria were also observed in several osteosarcoma, lung, and renal cancer cells after exposure to hypoxia; in contrast to fluid influx, this was due to MFN1-mediated abnormal fusion events and protected hypoxic cells against apoptosis (42). Further, the expanded matrix physically modulates the cristae structure with a subsequent change in mitochondrial functions such as oxidative phosphorylation, membrane transporter activity, and apoptosis (48). While we observed both the swelling of the matrix and the loss of cristae organization in the MOSE-L and MOSE-L_TIC_ *_v_*, we did not find aberrant fusion events and the expression of MFN1 and OPA1 were low (**Figure 4**). Further, the swelling was associated with an increased rather than a lower membrane potential, a higher rate of proliferation (28), and a metabolic switch to glycolysis (25, 26). How these events are coordinated and how the expansion of the matrix allows for enhanced proliferation and survival needs to be investigated in more detail.

Mitochondrial fusion and fission events support the adaptation to cellular energy demands and ensure quality control of the mitochondria (6). This may be different in cancer cells since in some cancers (including MCF7 breast cancer cells), fission promotes a proliferative advantage in driving stemness in tumor cells (15). The filamentous, branched mitochondrial network that is highly connected has been suggested to support ATP synthesis in oxygen-low areas of the cell (49). In contrast, other studies found that fragmented and dense mitochondria increased respiration (50) or reduced respiration and proliferation (51). Most studies targeting mitochondrial dynamics in ovarian cancer focus on overexpression and knockout of the regulatory proteins or toxic and pharmacological compounds that may not reflect physiologic events (52). In our study, we show that the filamentous morphology found in benign cells was changed to rounded, larger mitochondria in adherent cancer cells and fewer and smaller mitochondria in the core of the cancer spheroids upon aggregation and exposure to hypoxia (**Figures 1** and **6**). The altered mitochondrial morphology was not caused by overexpression of fission proteins since both fission and fusion proteins were lower in the cancer cells than in the benign cells; however, the ratio of fission to fusion proteins was elevated, indicating that the dynamic events were driven towards fission (**Figure 4**). This shift in the balance of these proteins has been previously observed with other cancers, including pancreatic, breast, head and neck squamous cell carcinoma, and lung cancer (13, 53, 54) and the expression of increased fission proteins has been correlated to decreased drug sensitivity and uncontrolled proliferation (55). Super-resolution microscopy provides quantitative analyses of mitochondrial numbers and size in sub-sections of a single cell (**Figures 5** and **6**). Our data demonstrate that the size of the mitochondria becomes smaller during progression while the total protein does not change; this may be due to fragmentation rather than enhanced fission since the expression of regulatory proteins was significantly lower in the cancer cells despite their higher total mitochondrial mass (**Figure 4**). This observed imbalance of mitochondrial dynamic proteins in relation to structure has not only been implicated in cancer but also neurodegenerative disorders (14, 56) and regulation of apoptosis (11, 12, 57). Both MOSE-L and MOSE-L_TIC_ *_v_* respond to aggregation with a decrease in mitochondrial volume in the core of the spheroid while the metabolically flexible MOSE-L_TIC_ *_v_* (25) further reduce mitochondria size in hypoxia (**Figure 6**). Only cells on the surface of the spheroids are actively dividing (unpublished data); the surface cells also contain larger and more numerous mitochondria. Importantly, we have recently shown that aggregation and hypoxia lower the proliferation rate and respiration of MOSE spheroids with a stronger response of the MOSE-L_TIC_ *_v_* (27). Together, our results suggest that the fragmentation of the mitochondria is the result of cellular adaptation to an environment that is low in nutrients and oxygen with a reduced growth rate and energy production.

Mitophagy removes damaged or depolarized mitochondria and is upregulated in response to stressors such as nutrient deprivation and hypoxia to reduce mitochondrial mass and may promote survival (58). Mitophagy is preceded by mitochondrial fragmentation that allows for isolation and engulfment of the damaged mitochondria (59) and loss of membrane potential (39, 54). Our data show that the number and size of autophagosomes increased during malignant progression especially in hypoxia but this was not accompanied by the loss of mitochondrial membrane potential (**Figure 3**). This has been previously reported in other cancers including hepatic and metastatic breast cancer (60). Other studies, however, have shown contradictory results where hypoxia depolarizes mitochondria and leads to an induction of autophagy (61, 62). This may be due to the differences in methodology i.e., hypoxia levels, use of dye in concentrations that induce quenching, or the culture conditions permissive or non-permissive for growth and cellular functions (25, 63). It can be speculated that the increase in mitophagy that is regulated independent of the mitochondrial membrane potential may be counteracted by mitobiogenesis to maintain a level of healthy mitochondria (64, 65). The balance of mitophagy upon aggregation is then shifted favoring mitophagy rather than mitobiogenesis. This may limit ROS production and maximize oxygen use to increase the bioenergetic capacity of cells, supporting growth and survival of the cancer cells (54, 66). Future studies will investigate changes in mitobiogenesis/mitophagy balance during progression and aggregation.

Mitochondrial respiration is a major producer of ROS. High ROS levels can induce DNA mutations involved in tumor initiation and progression while excessive levels cause protein oxidation, lipid peroxidation, and promote apoptosis. ROS also activates signaling pathways that regulate cellular functions and proliferative pathways such as the PI3K and MAPK pathways (16–20). The transformed MOSE cells exhibit higher membrane potentials than the benign cells especially in hypoxic conditions (**Figure 3**). High mitochondrial membrane potential can support cell viability and apoptosis (67) but a remarkable heterogeneity in mitochondrial membrane potential has been observed *in vivo* (68) that may be affected by the cells’ localization and access to oxygen and nutrients. Concurrent with our results, *in vitro* studies have shown higher mitochondrial membrane potential in breast, prostate and lung cancer and glioblastoma cell lines (69–71) that may be the result of higher rhodamine uptake and retention (72). The lower ROS levels despite the higher membrane potential that indicates high electron transport chain (ETC) activity in the fast-growing MOSE-L_TIC_ *_v_* cells suggest that these cells can either reduce the generation of ROS or control ROS levels by pathways other than SOD, to limit oxidative stress-induced cell death and promote a beneficial redox balance (73–75).

Interestingly, the MOSE-L_TIC_ *_v_* cells have stem-like properties; the injection of only 10 cell causes lethal disease (unpublished data). Cancer stem cells often exhibit low levels of ROS that allow for the expression of stem cell markers, the reprogramming of metabolism (76) and the resistance to apoptosis (77). Studies comparing the generation of ROS in benign and cancer cells in hypoxia have resulted in conflicting results showing either ROS generation in response to HIF1α signaling or a steady state (78, 79). Cell type-specific responses in addition to experimental designs and investigated timepoints can contribute to these results. Further differences may arise from the use of DCFDA that in addition to H_2_O_2_ also identifies other radicals and therefore may indicate an increase in general oxidative stress and total radicals (80, 81). In addition, hypoxia has been shown to influence ROS production by acting specifically on complexes I, II, and III of the ETC (82, 83). The observed increase in ROS in hypoxia in our study did not show a decrease in the expression of a key scavenging enzyme SOD2. However, the activity of scavenging enzymes has not been evaluated in this current study. Other studies have indicated an increase in SOD2 expression after resveratrol treatment that was paralleled by elevated ROS levels; albeit, SOD2 activity was significantly reduced (84). Thus, future studies will examine the activity of scavenger enzymes and the role of ROS production on metabolic adaptations in the maintenance of viability, stemness, and redox balance in relation to peritoneal conditions in our ovarian cancer model.

UCPs are integral membrane proteins that reside in the inner mitochondrial membrane that are important in regulating protein leak and thermogenesis (22, 23). However, UCP2 and 3 have recently been identified as not only important in regulating proton leaks by negatively regulating mitochondrial membrane potential and ATP production during oxidative phosphorylation but to have a potential for regulating superoxide formation as well (85, 86). Paradoxically, UCPs have been shown to also be activated by superoxide production to prevent subsequent proton leak and oxidative stress (22, 23, 87). Recent studies have found that high levels of UCP2 and UCP3 appear to be beneficial for cancer cells as well as influence mitochondrial structural adaptation. UCP2 has been shown to improve detrimental mitochondrial fragmentation in kidney cells by a preserving mitochondrial integrity through stabilizing membrane potential and reducing mitophagy (88). Further, UCP2 and UCP3 activation can reduce ROS (42, 89) which is thought to promote chemoresistance (90). Inhibition of UCP2 and UCP3 increased ROS formation, reduced viability via autophagic cell death, and increased the toxic effects of chemotherapeutics in breast cancer cells while high UCP2 levels confer a poor prognosis to breast cancer patients (91). Interestingly, the activation of UCP2 also increases cancer proliferation (92) potentially by increasing glycolysis (93) and the expression of genes that promote mitobiogenesis (94). Thus, the high levels of UCP2 and UCP3 observed in the cancer spheroids could contribute to the regulation of ROS levels, survival, and proliferation.

In summary, this is a proof-of-concept study that shows that changes in mitochondrial morphology during ovarian cancer progression do not affect mitochondrial functions such as induction of apoptosis and respiration or compromise the cells’ viability. This study is novel because it qualitatively and quantitatively identifies structural mitochondrial adaptations during malignant progression in 2D and 3D culture in a syngeneic model representing benign, slow-developing, and fast-developing disease in more physiologically relevant conditions. Aggregation induced fragmentation of mitochondria which correlates well with a reduced respiration and proliferation (27). We find that the highly aggressive MOSE-L_TIC_ *_v_* ovarian cancer cells are more adept to survive conditions non-permissive for epithelial cells potentially through alterations in mitochondrial morphology and function in comparison to our benign MOSE-E and slow-developing MOSE-L cells. This includes enhancing autophagy and increasing UCP expression to counteract cellular oxidative stress and increasing mitochondrial fragmentation through an imbalance of dynamic protein expression to compromise integral mitochondrial ultrastructure. Understanding how these molecular events and phenotypic changes that enable survival of metastatic cancer cells in an environment low in oxygen and nutrients are regulated may provide specific targets for interventions to suppress metastatic outgrowth of disseminating cells. Future studies will be dedicated to identifying if suppression of these molecular adaptations of mitochondrial dynamics will limit aggregation capacity and survival of disseminating ovarian cancer cells.

## Data Availability Statement

The raw data supporting the conclusions of this article will be made available by the authors, without undue reservation.

## Author Contributions

JG and ES wrote the manuscript. JG, ES, JS, NS, and SC made substantial contributions to conception and design of experiments. JG, ES, MA, JP, YW, AR, and YS participated in acquisition, analysis, and interpretation of *in vitro* experiments. ES, JS, DB, and NS provided technical and materials supports. JG and ES participated in in-depth analysis and interpretation of results. ES design and supervised the study. All authors contributed to the article and approved the submitted version.

## Funding

This work is supported by the USDA National Institute of Food and Agriculture Hatch project 1006578 (ES), CEH seed funds (ES), NIH NHLBI R01 grant HL132236 (JS), NIH Grant R01 CA200755 (NS), UVA Cancer Center Seeds Funds (NS). NS was supported in part by the National Institutes of Health’s National Center for Advancing Translational Sciences under Award number UL1TR003015 and R01 CA200755. This content is solely the responsibility of the authors and does not necessarily represent the official views of the National Institutes of Health.

## Conflict of Interest

The authors declare that the research was conducted in the absence of any commercial or financial relationships that could be construed as a potential conflict of interest.

## Supplementary Material

Supplementary Video 1 Mitochondria were stained as described under methods and materials, and the images were taken on Leica SP8 DMi8 microscope.

## Abbreviations

DRP1, dynamin related protein 1; FIS1, fission 1; HO, hypoxic; MFN1, mitofusin 1; MOSE, murine ovarian surface epithelial cells; MOSE-E, MOSE earlybenign; MOSE-L, MOSE late-tumorigenic; MOSE-L_TIC*v*_, MOSE tumor initiating cell variant; NO, normoxic; OPA1, optic atrophy protein 1; ROS, reactive oxygen species; SOD2, superoxide dismutase 2; STORM, stochastic optical reconstruction microscopy; TMRM, tetramethyl rhodamine; TOMM 20, translocase of the outer mitochondrial membrane protein 20; UCP, uncoupling.
