# Supplementary material for: Progression-Mediated Changes in Mitochondrial Morphology Promotes Adaptation to Hypoxic Peritoneal Conditions in Serous Ovarian Cancer
Source: Front Oncol. 2021 Jan 13;10:600113. doi: 10.3389/fonc.2020.600113 (PMC7838066; doi:10.3389/fonc.2020.600113)
Supplement: Supplementary file 2 [file Presentation_1.pptx]

## Slide 1
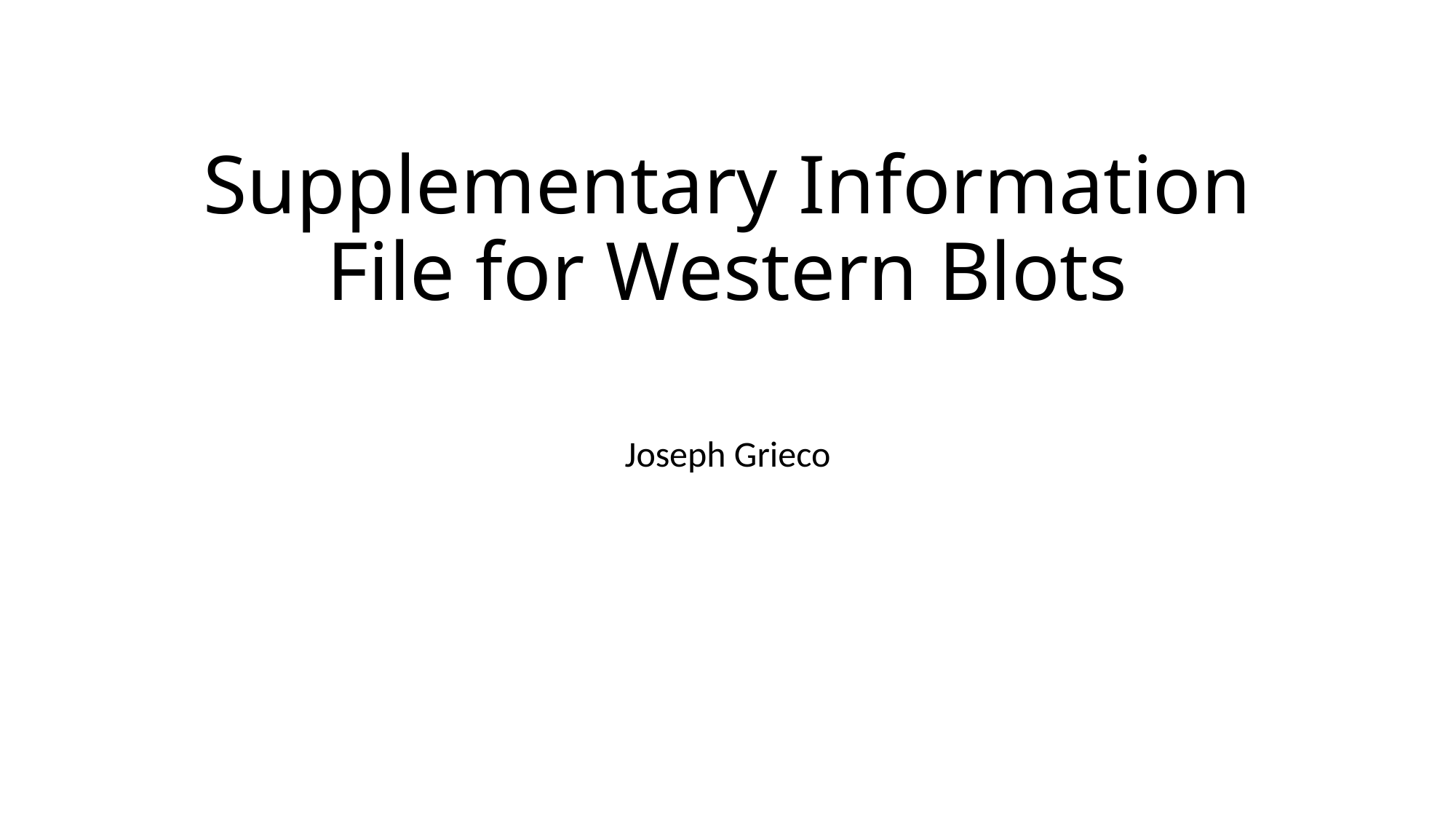

# Supplementary Information File for Western Blots
Joseph Grieco

## Slide 2
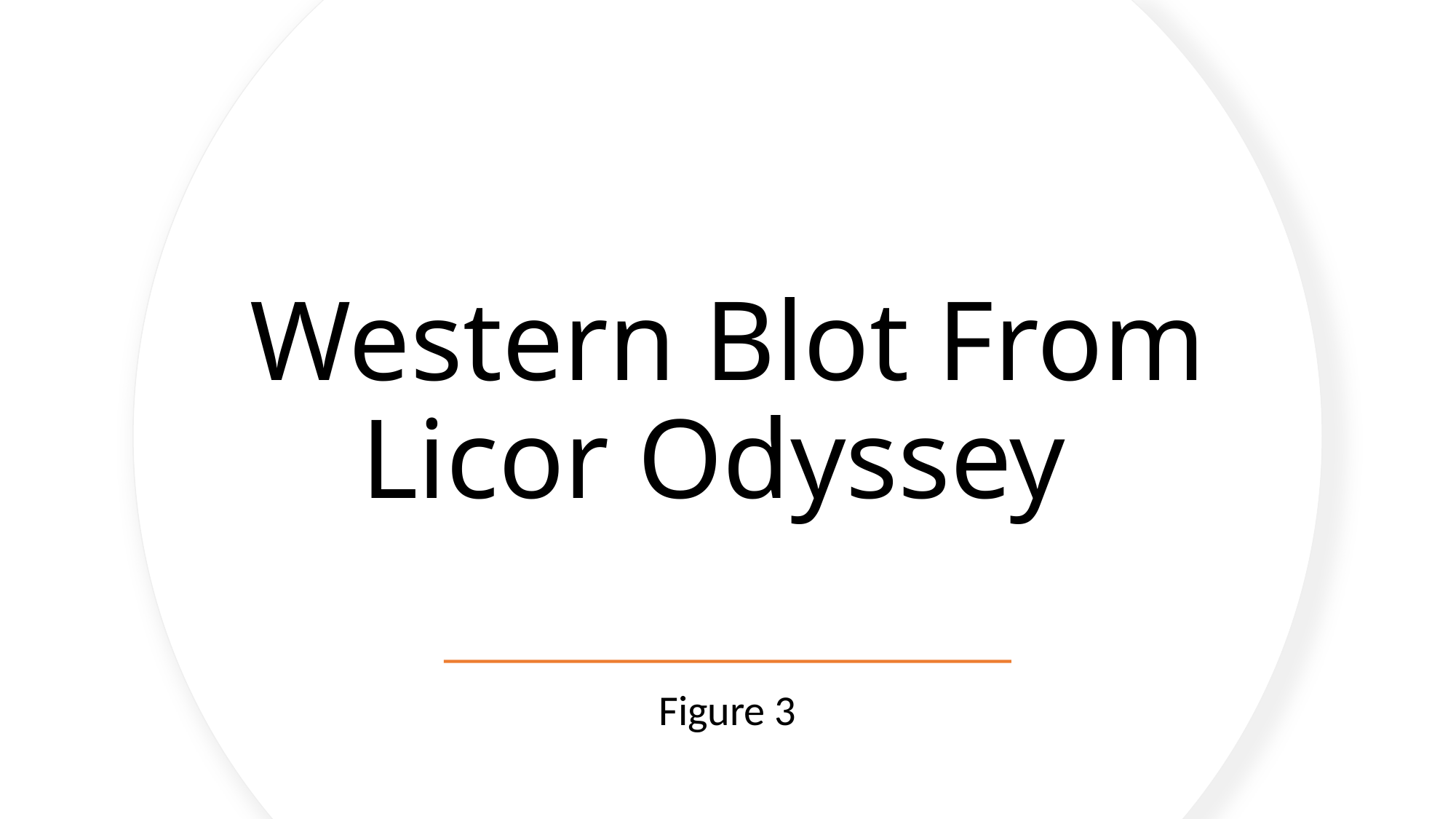

# Western Blot From Licor Odyssey
Figure 3

## Slide 3
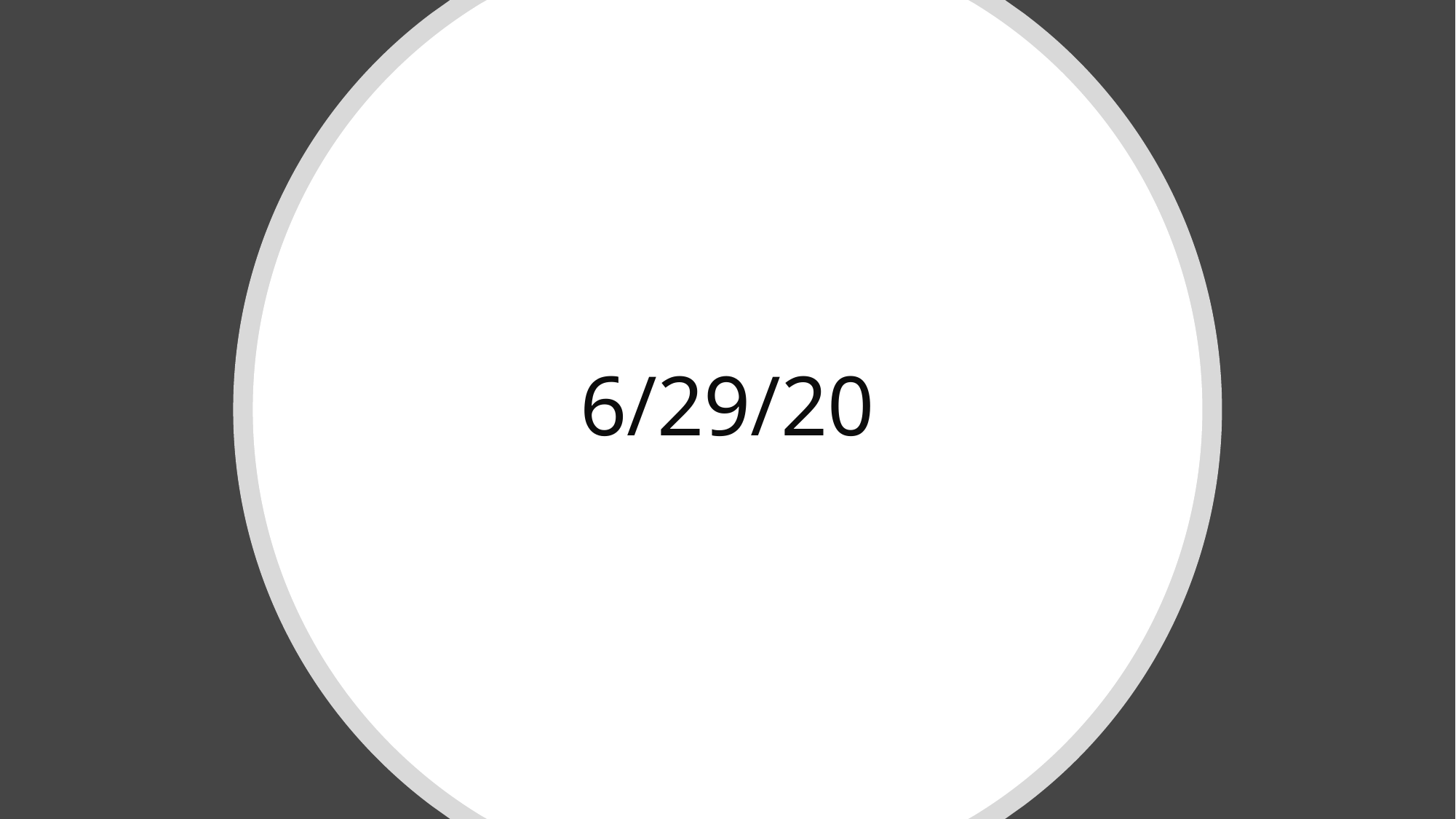

# 6/29/20

## Slide 4
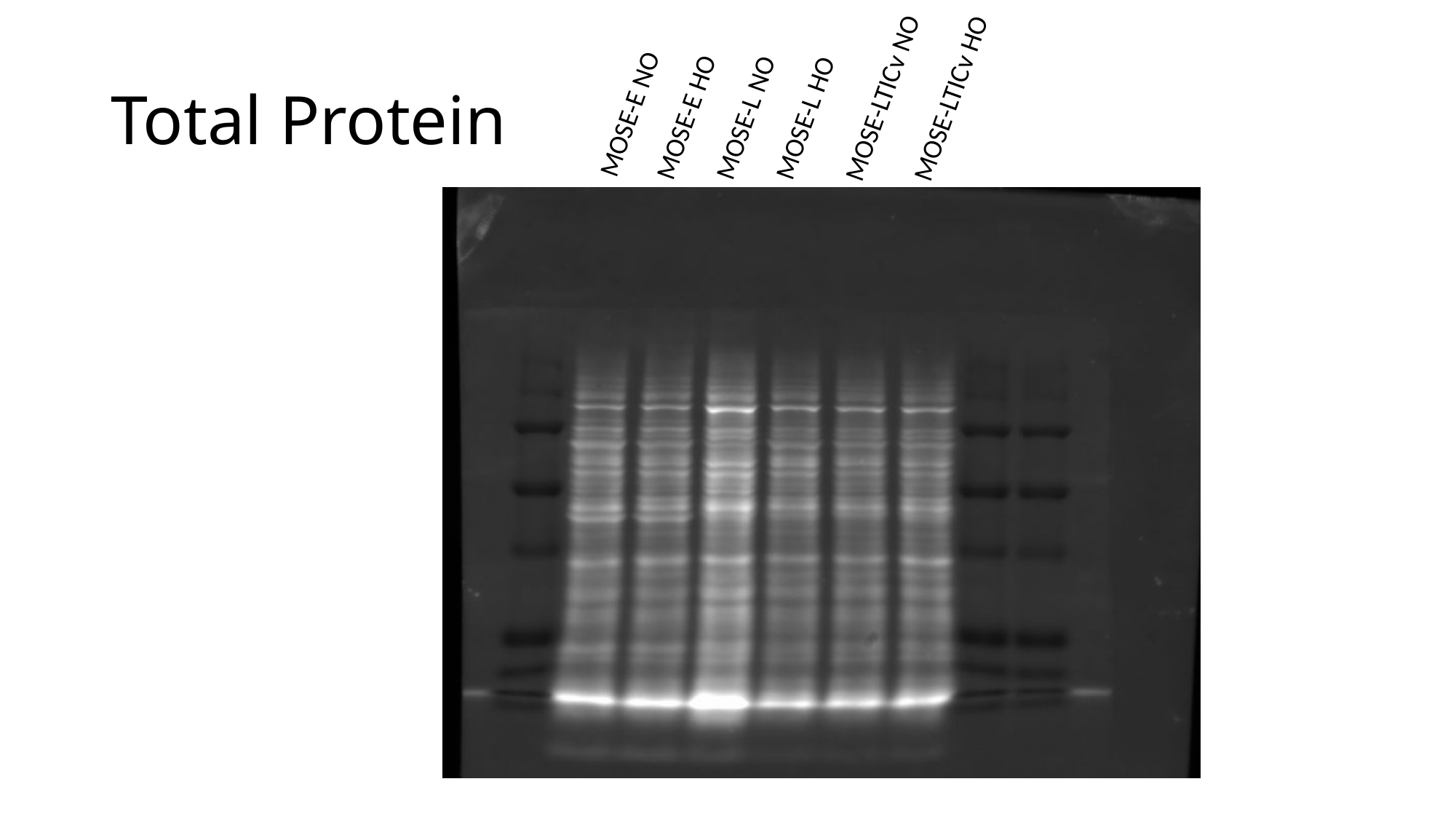

MOSE-LTICv NO
MOSE-LTICv HO
MOSE-E NO
MOSE-E HO
MOSE-L NO
MOSE-L HO
# Total Protein

## Slide 5
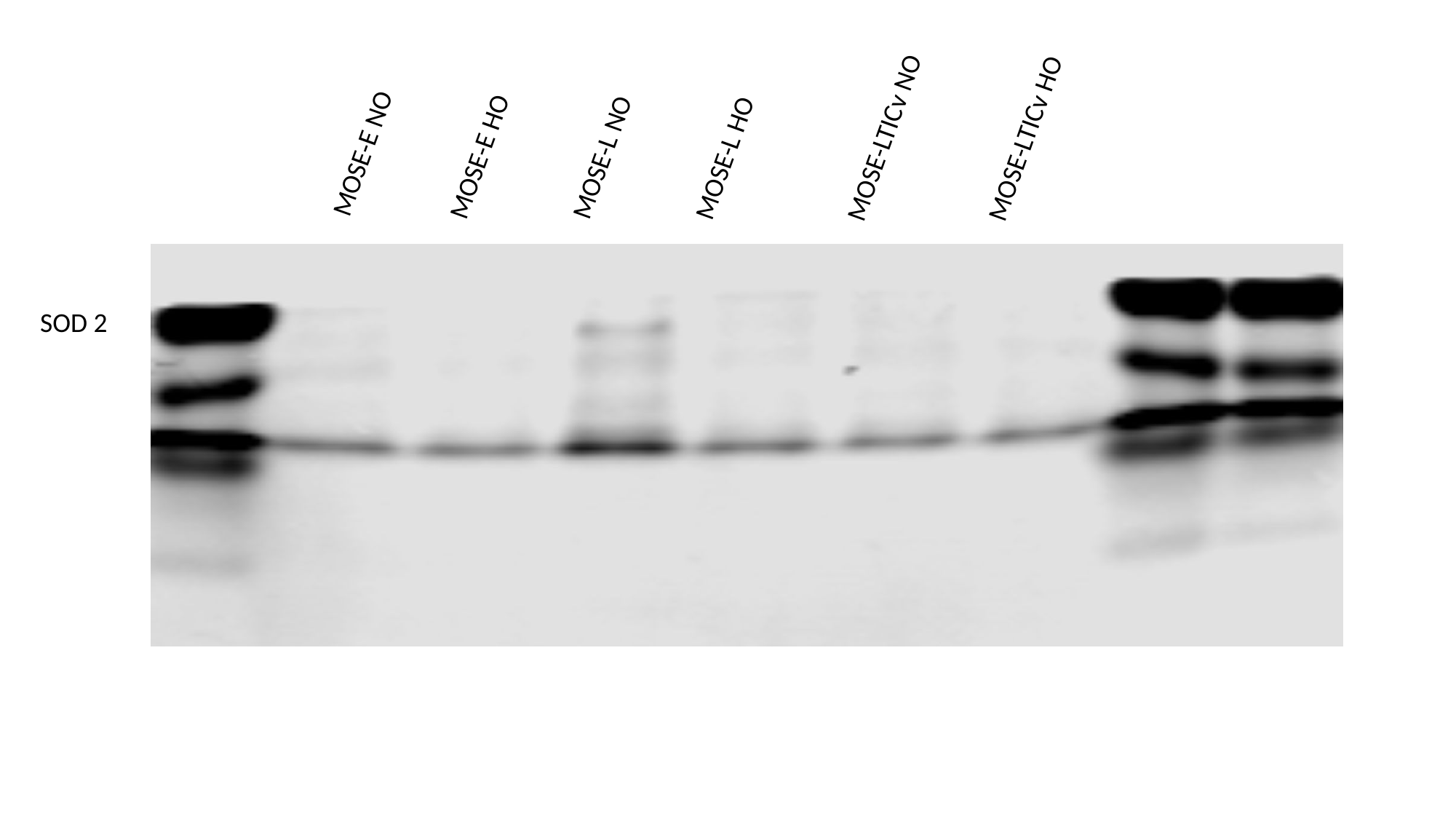

MOSE-LTICv NO
MOSE-LTICv HO
MOSE-E NO
MOSE-E HO
MOSE-L NO
MOSE-L HO
SOD 2

## Slide 6
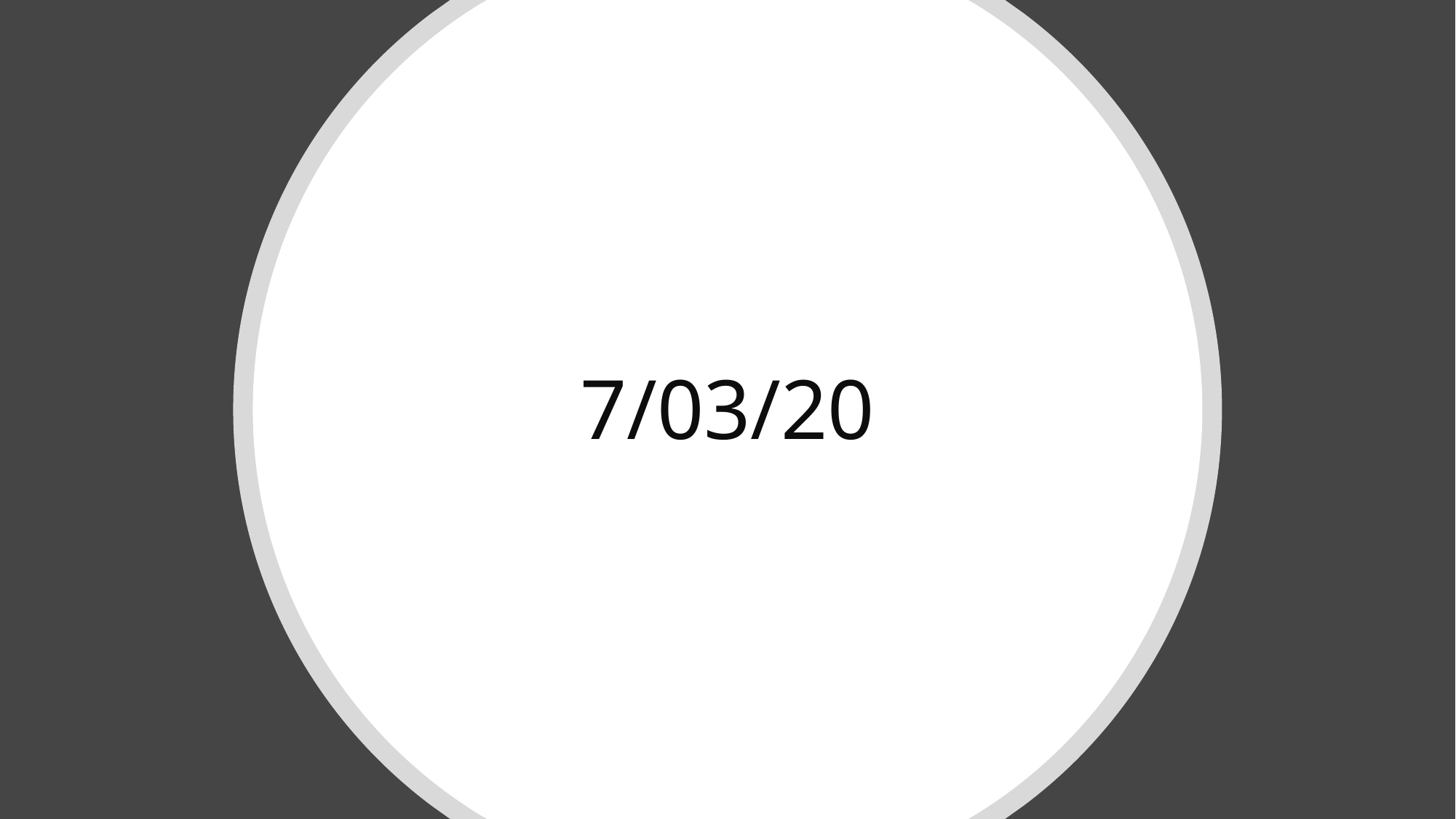

# 7/03/20

## Slide 7
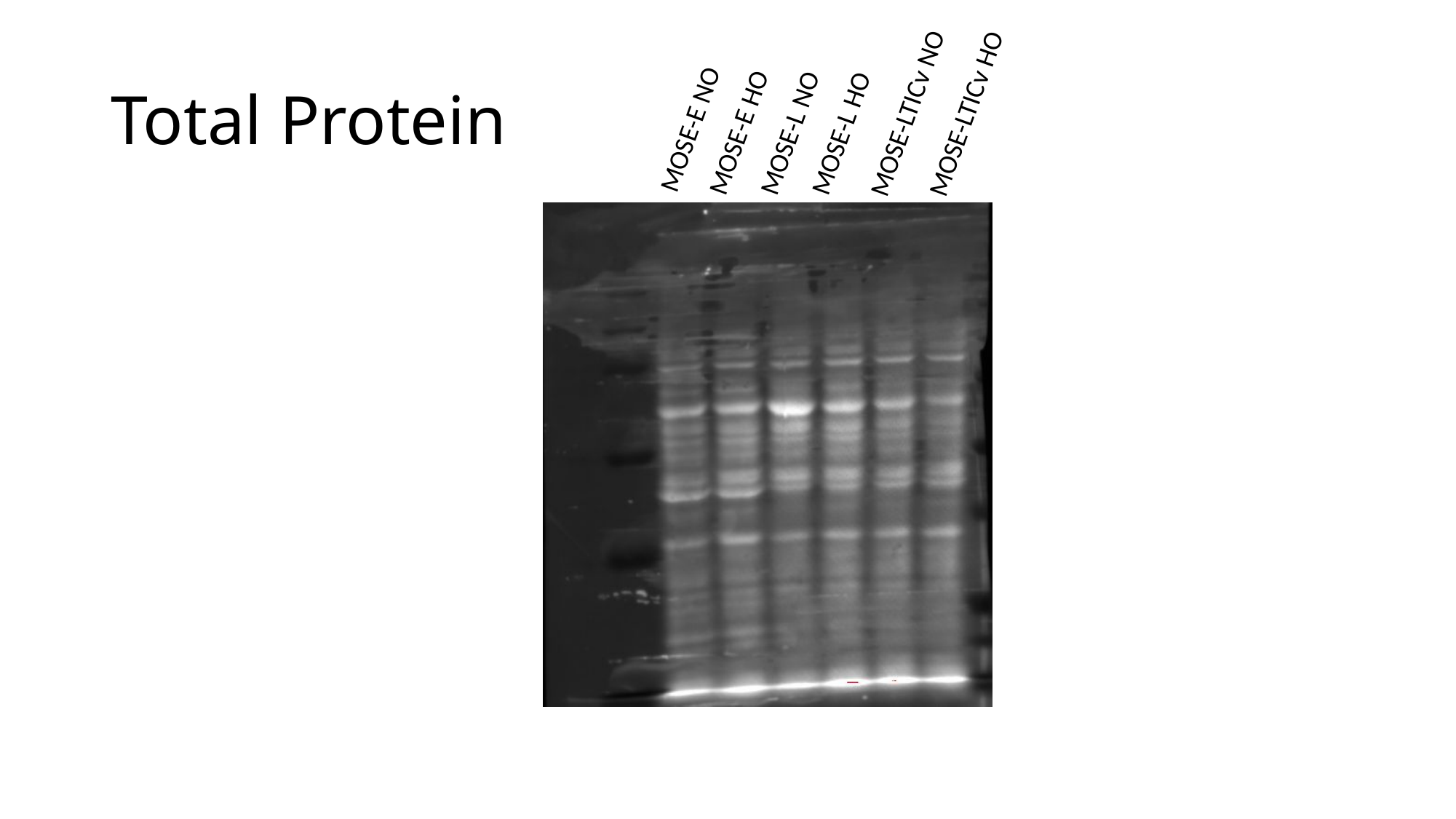

MOSE-LTICv NO
MOSE-LTICv HO
MOSE-E NO
MOSE-E HO
MOSE-L NO
MOSE-L HO
# Total Protein

## Slide 8
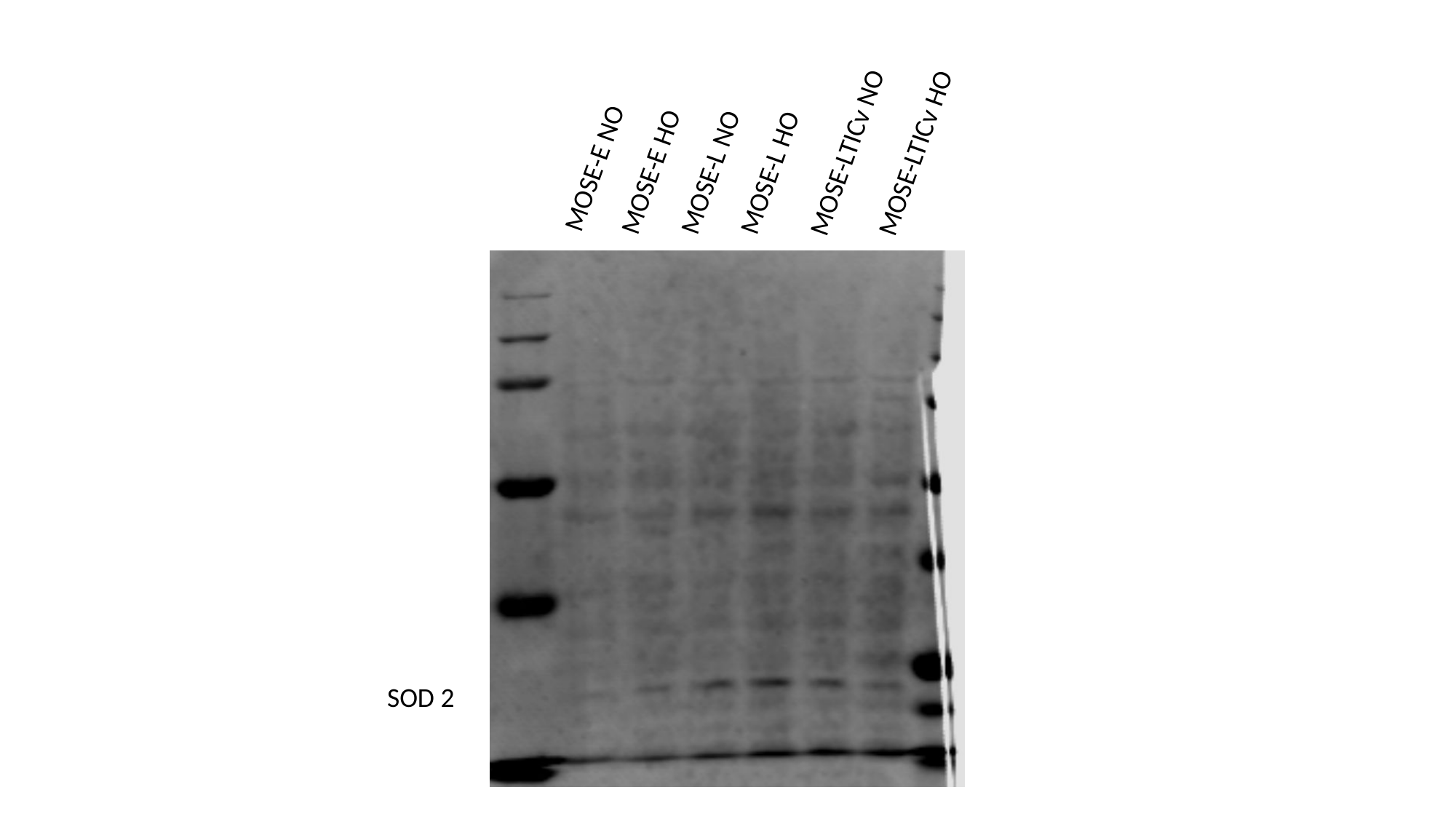

MOSE-LTICv NO
MOSE-LTICv HO
MOSE-E NO
MOSE-E HO
MOSE-L NO
MOSE-L HO
SOD 2

## Slide 9
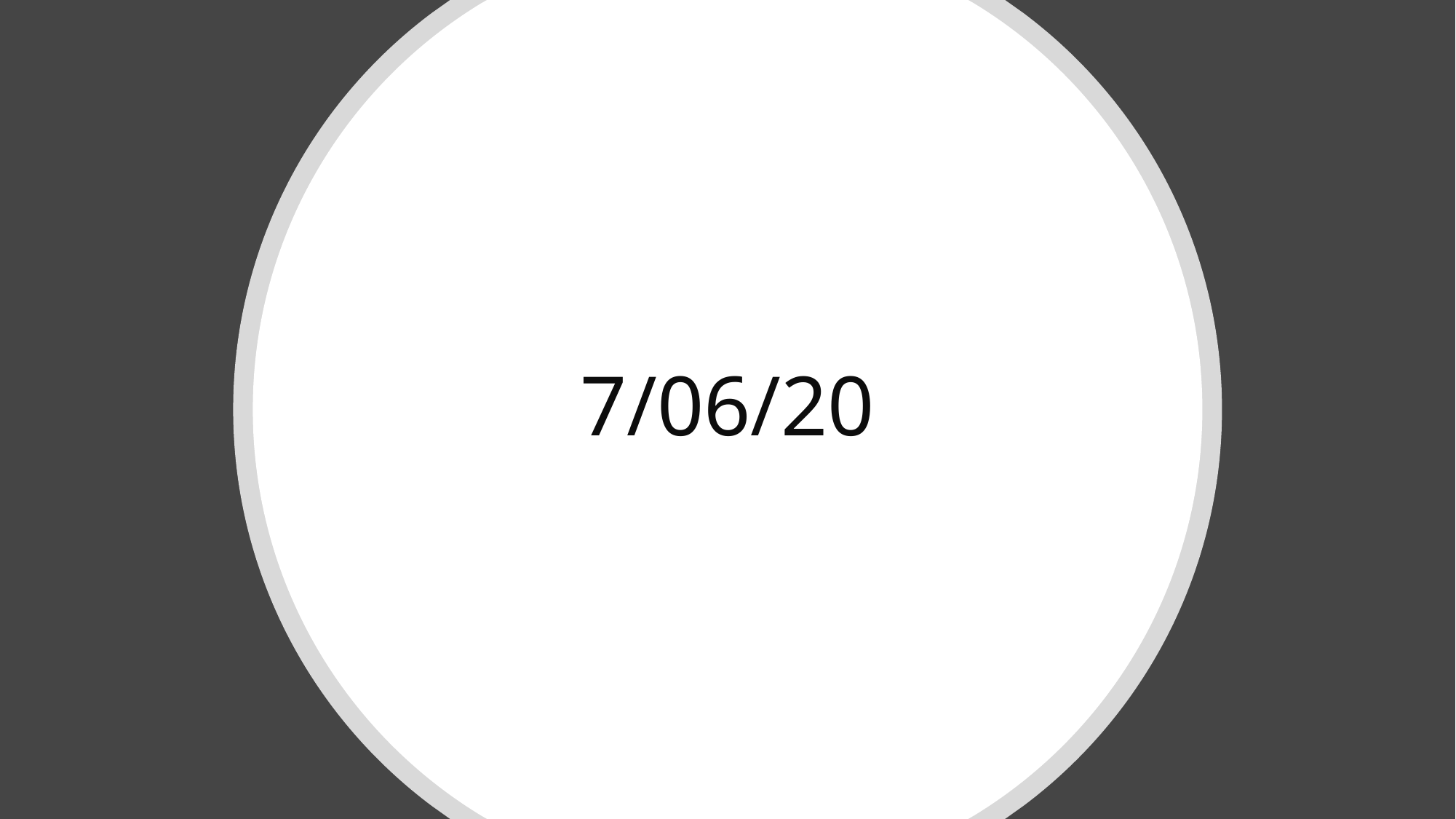

# 7/06/20

## Slide 10
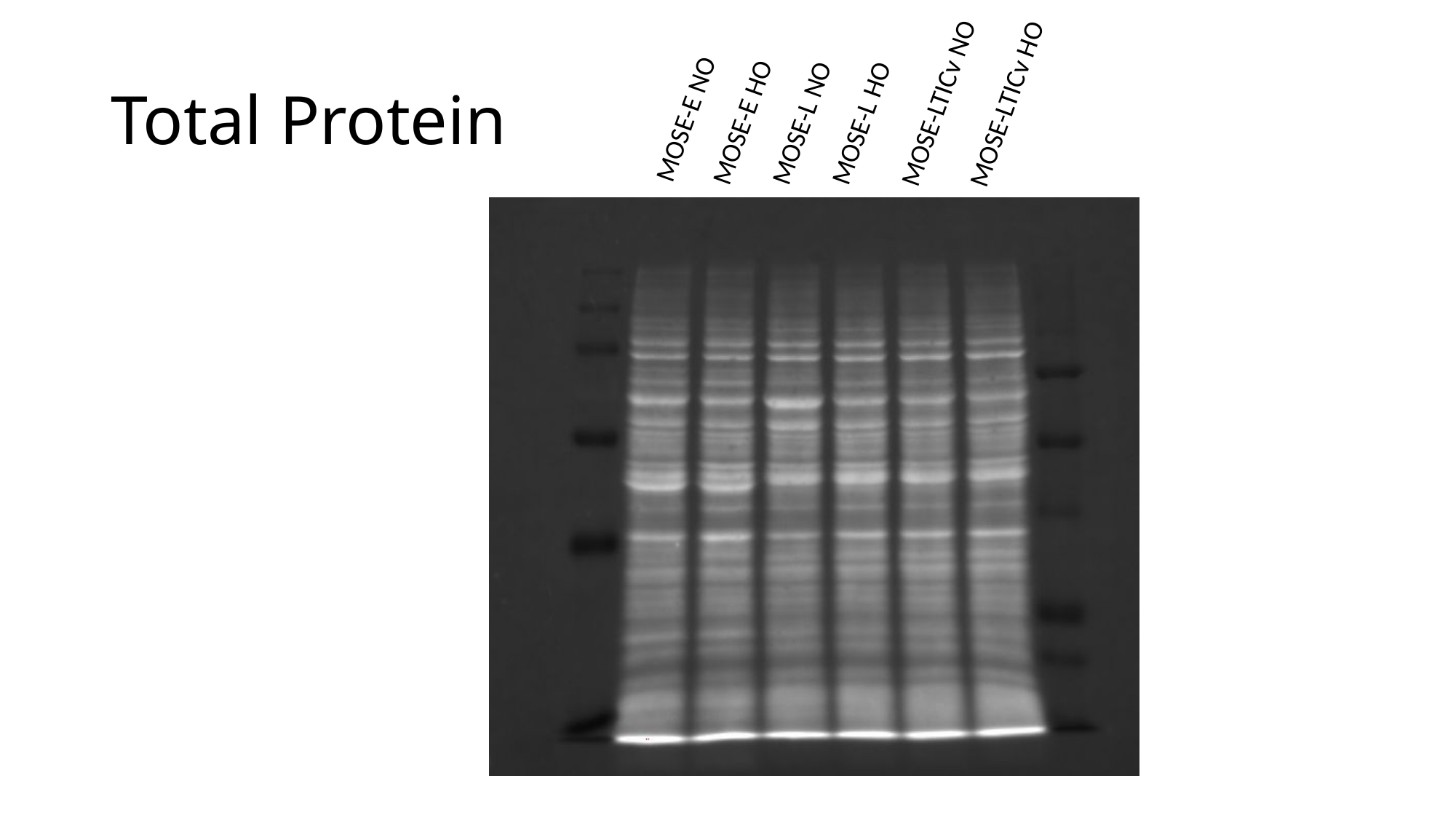

MOSE-LTICv NO
MOSE-LTICv HO
MOSE-E NO
MOSE-E HO
MOSE-L NO
MOSE-L HO
# Total Protein

## Slide 11
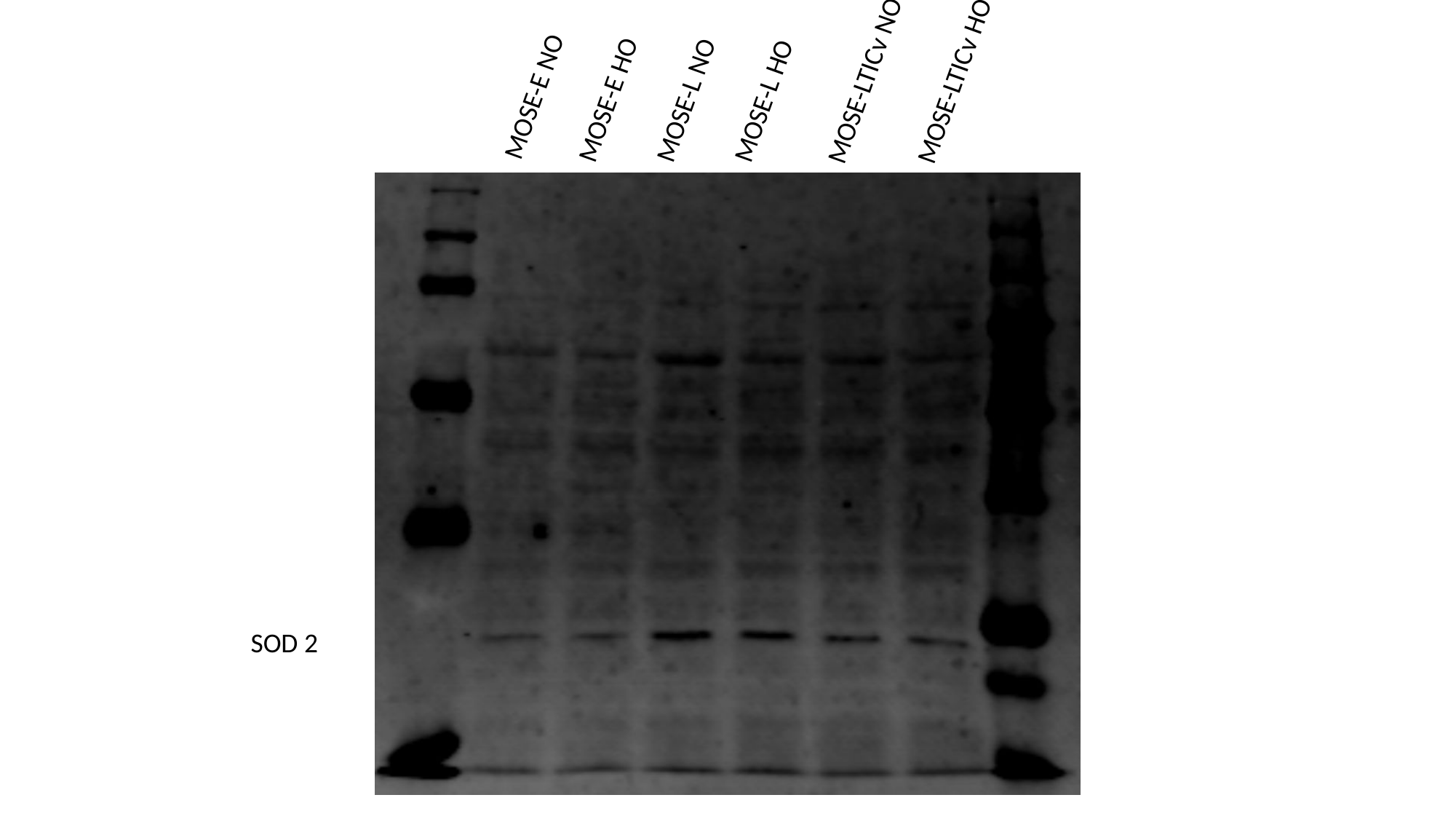

MOSE-LTICv NO
MOSE-LTICv HO
MOSE-E NO
MOSE-E HO
MOSE-L NO
MOSE-L HO
SOD 2

## Slide 12
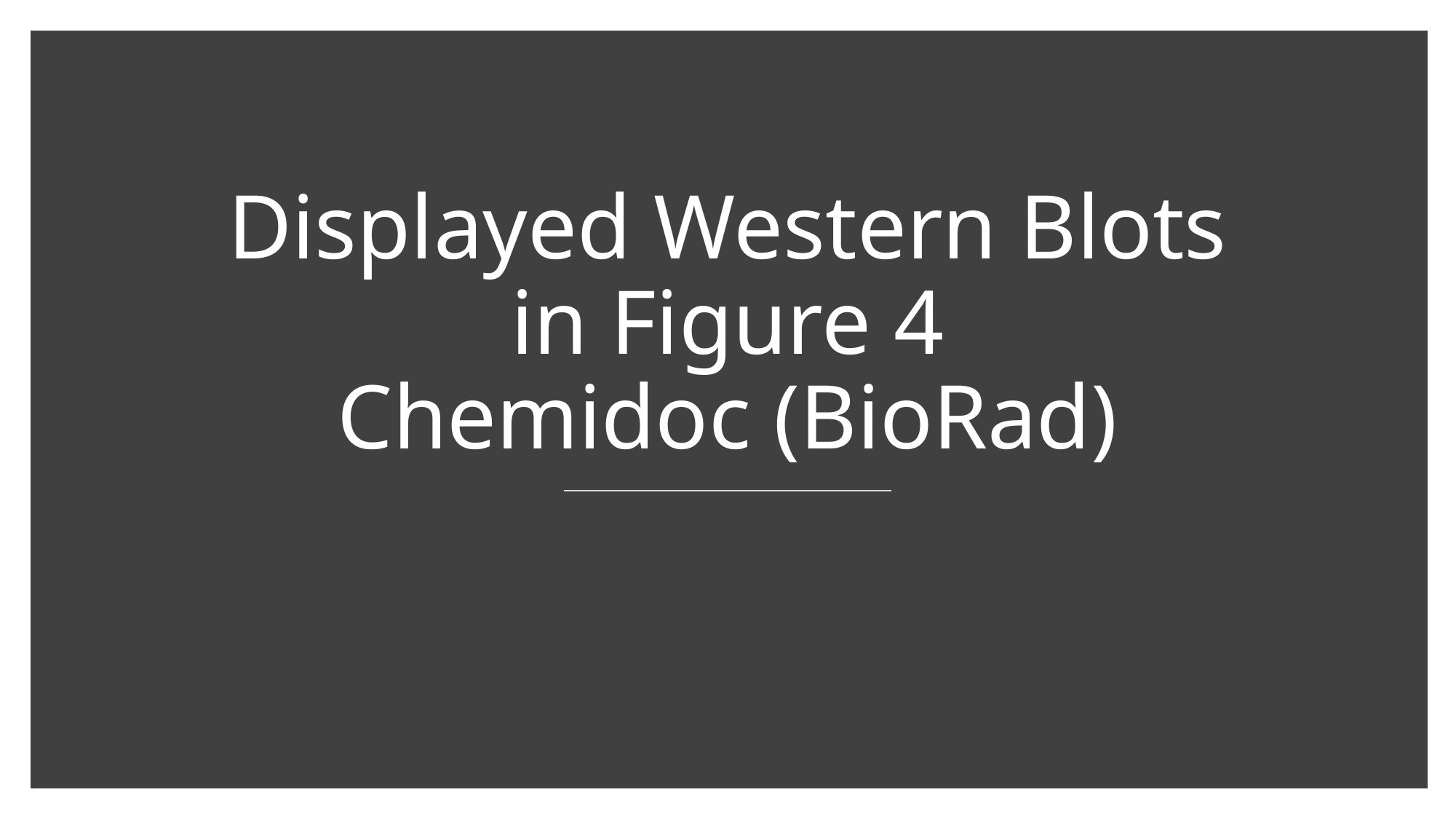

# Displayed Western Blots in Figure 4 Chemidoc (BioRad)

## Slide 13
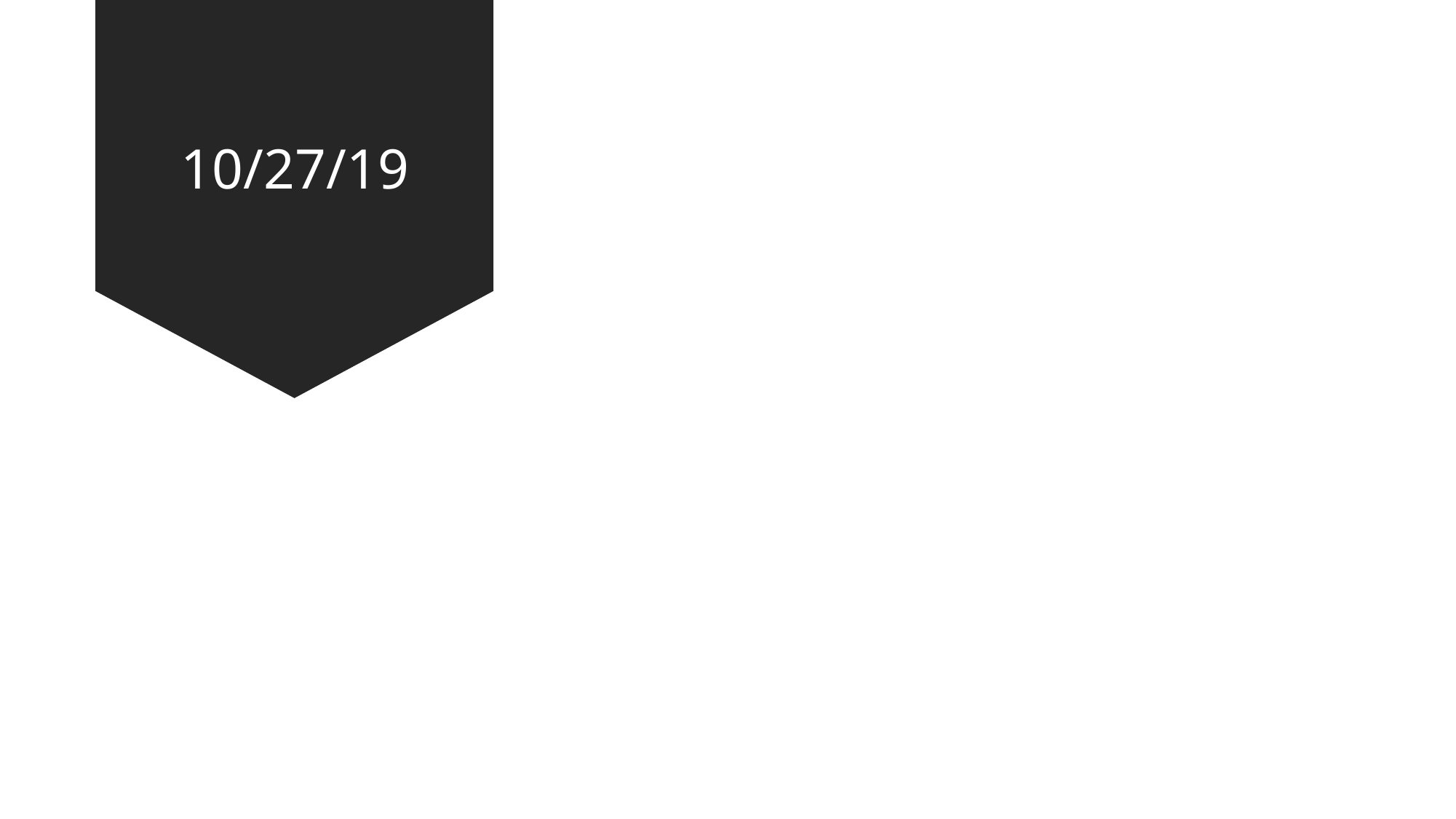

# 10/27/19

## Slide 14
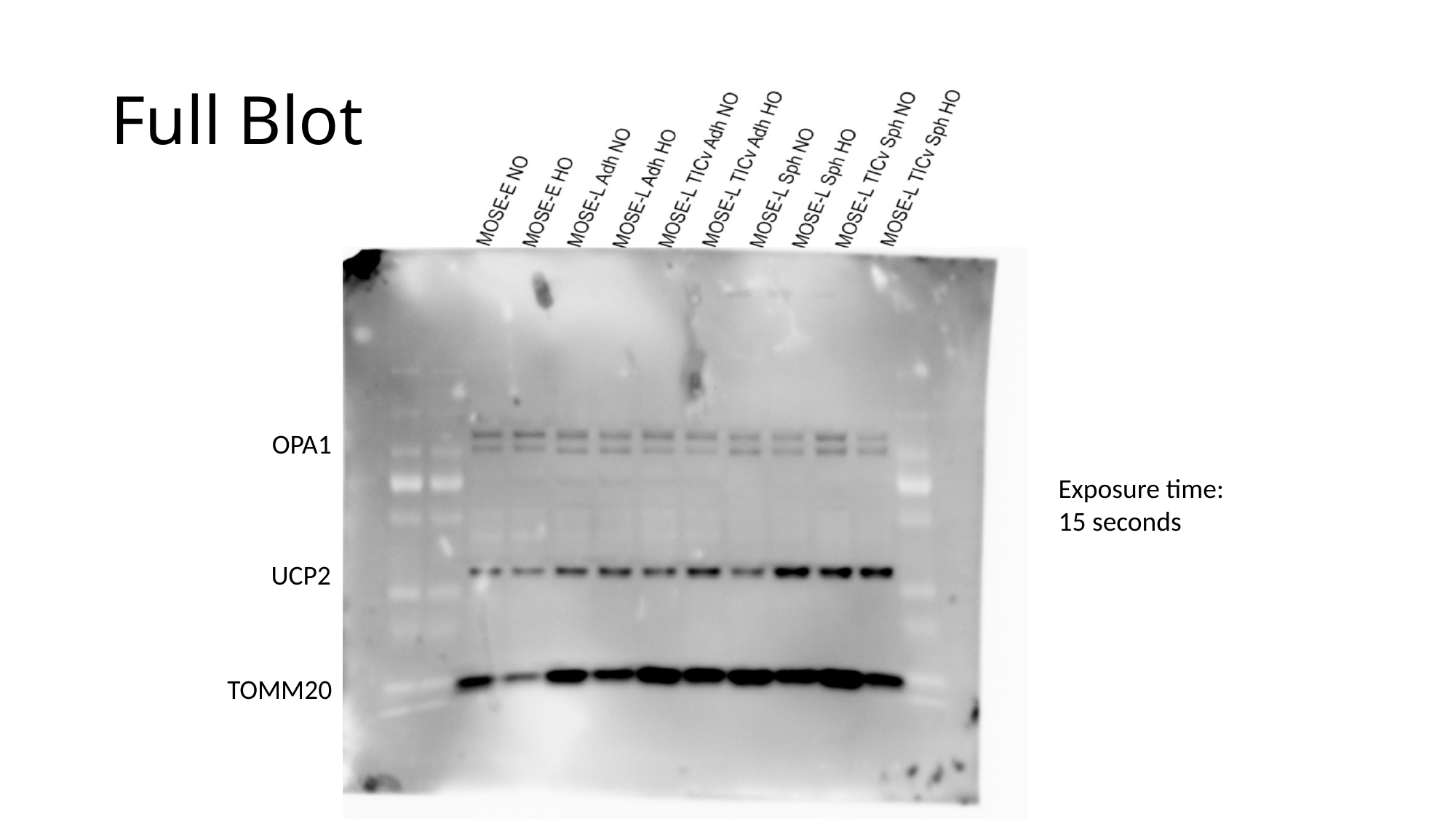

# Full Blot
OPA1
Exposure time: 15 seconds
UCP2
TOMM20

## Slide 15
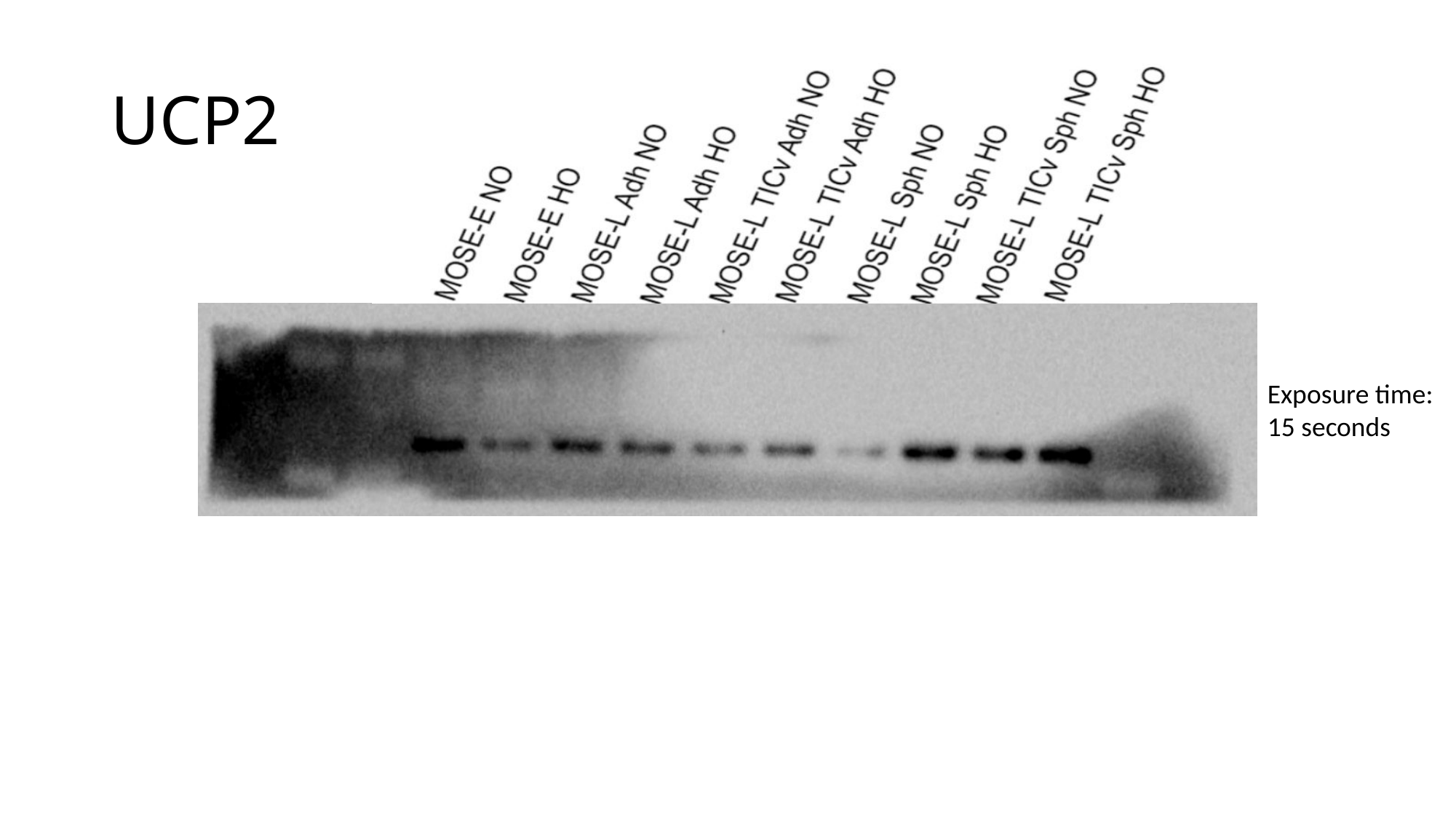

# UCP2
Exposure time: 15 seconds

## Slide 16
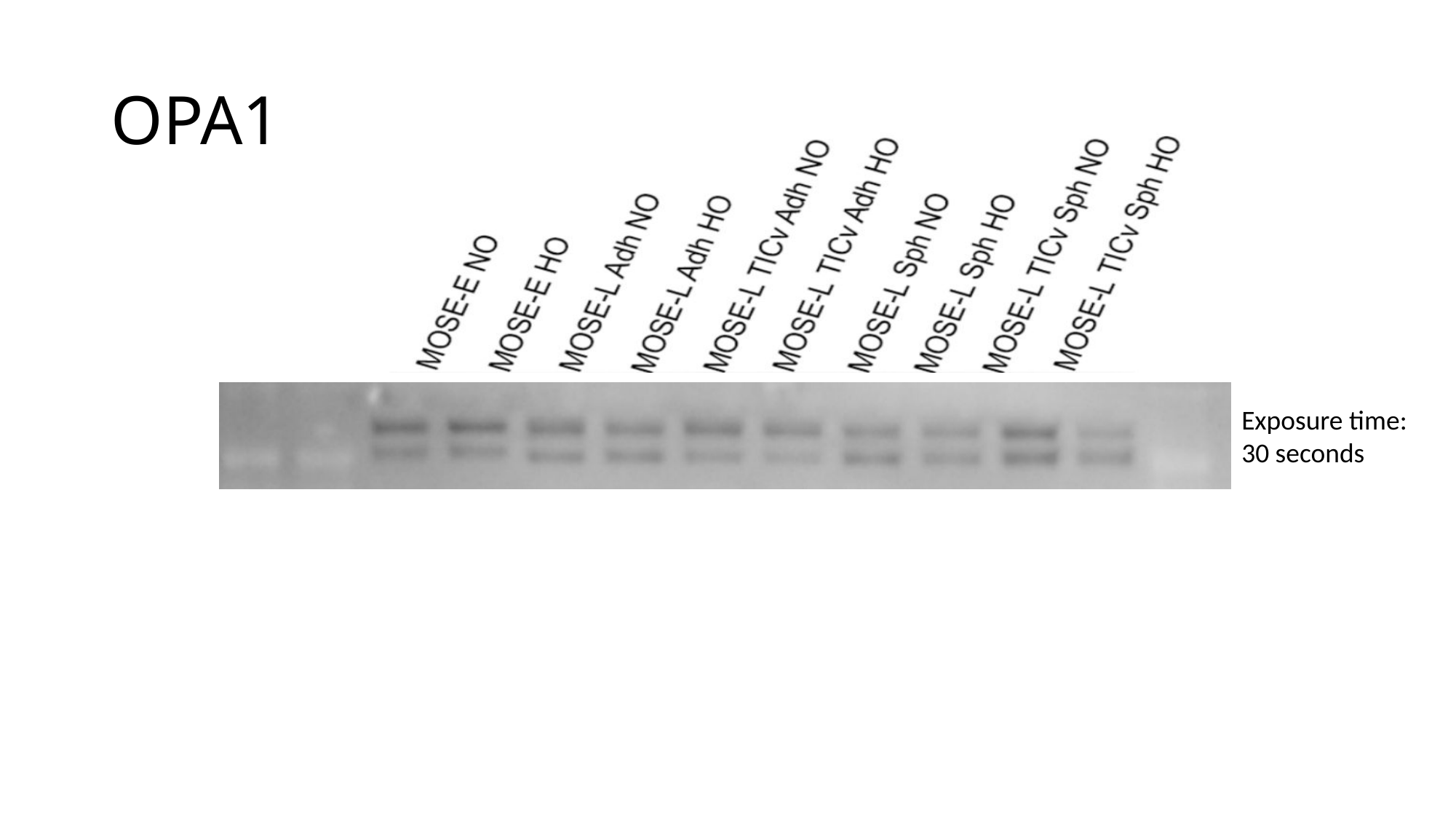

# OPA1
Exposure time: 30 seconds

## Slide 17
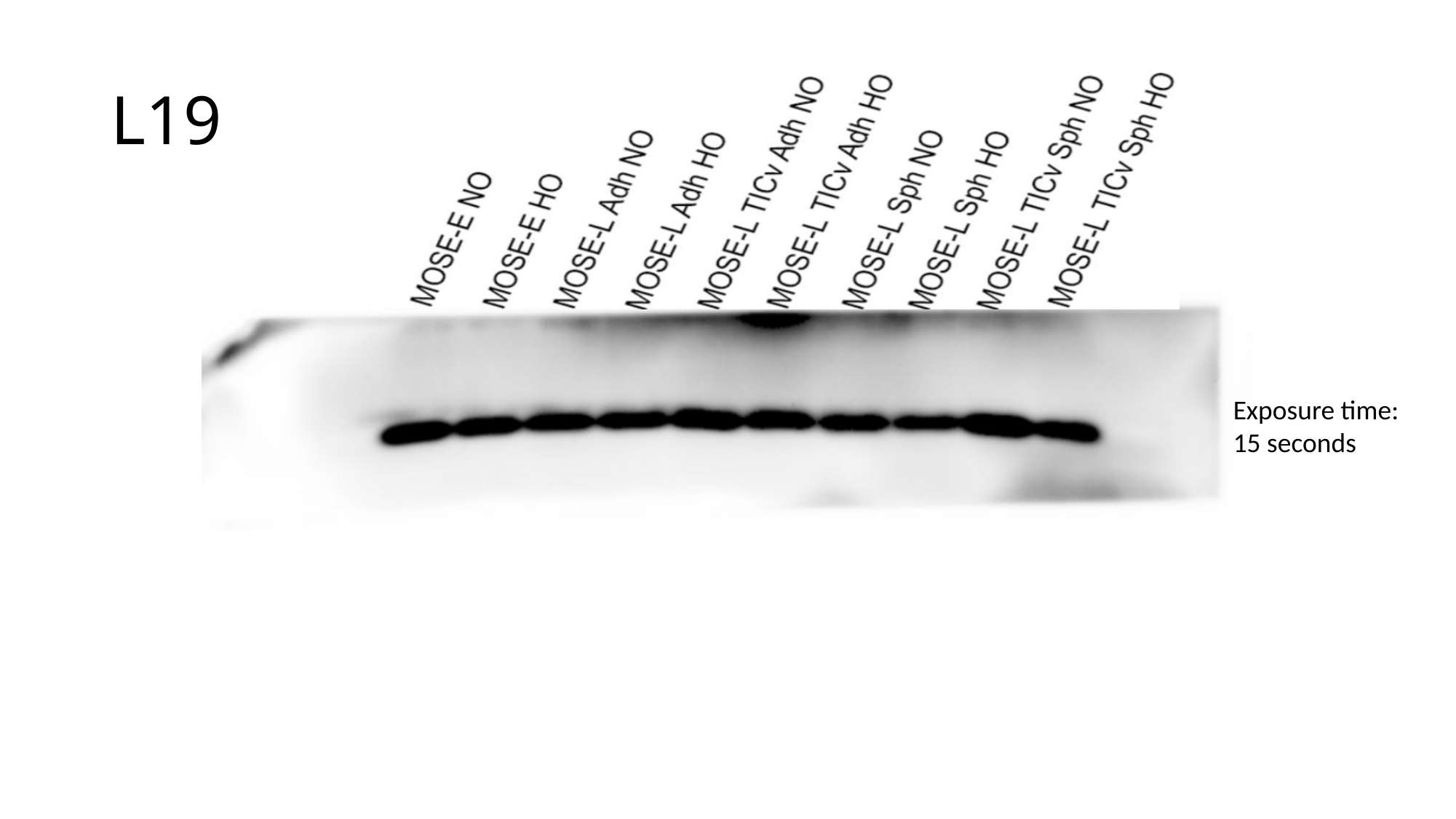

# L19
Exposure time: 15 seconds

## Slide 18
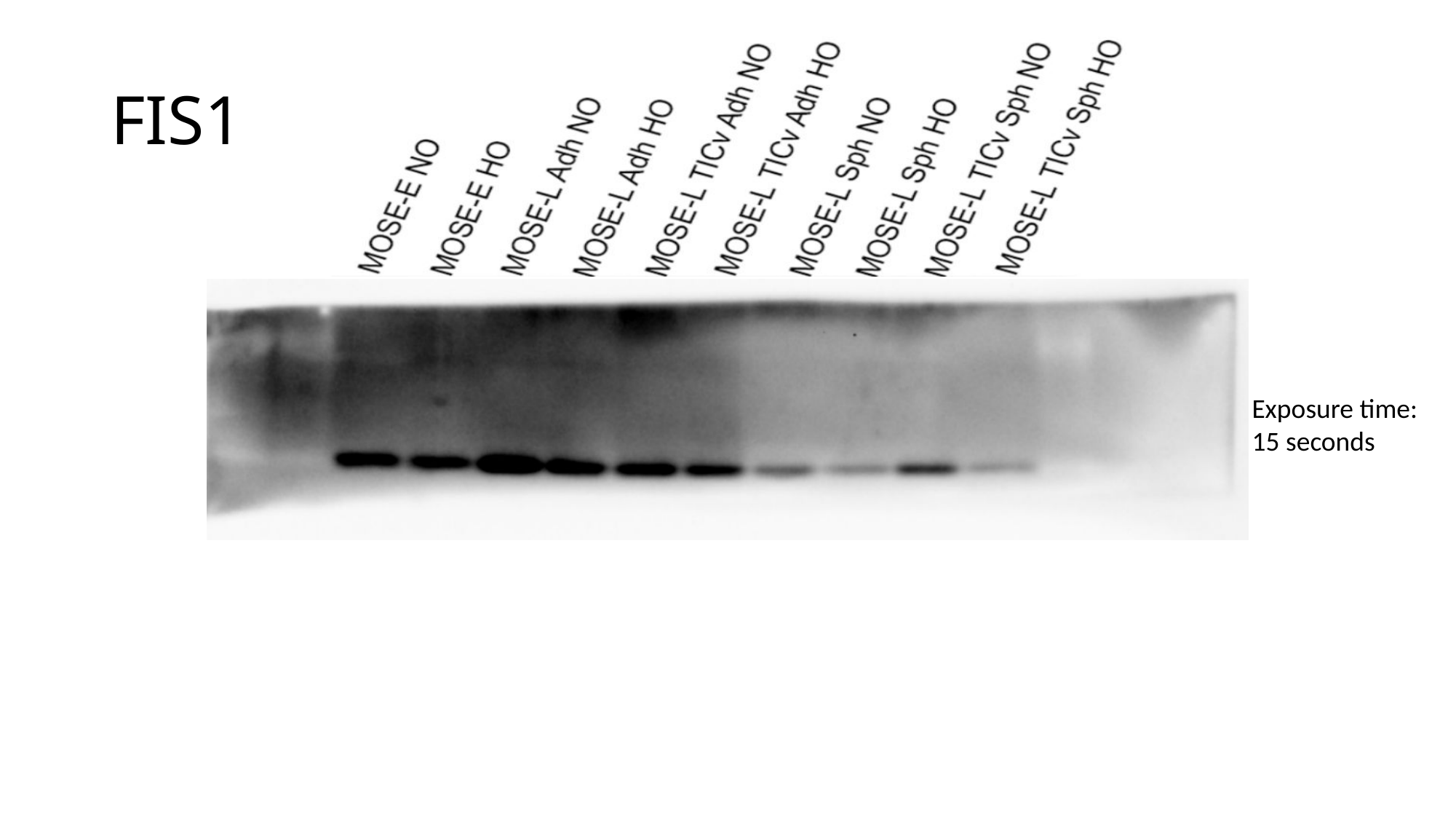

# FIS1
Exposure time: 15 seconds

## Slide 19
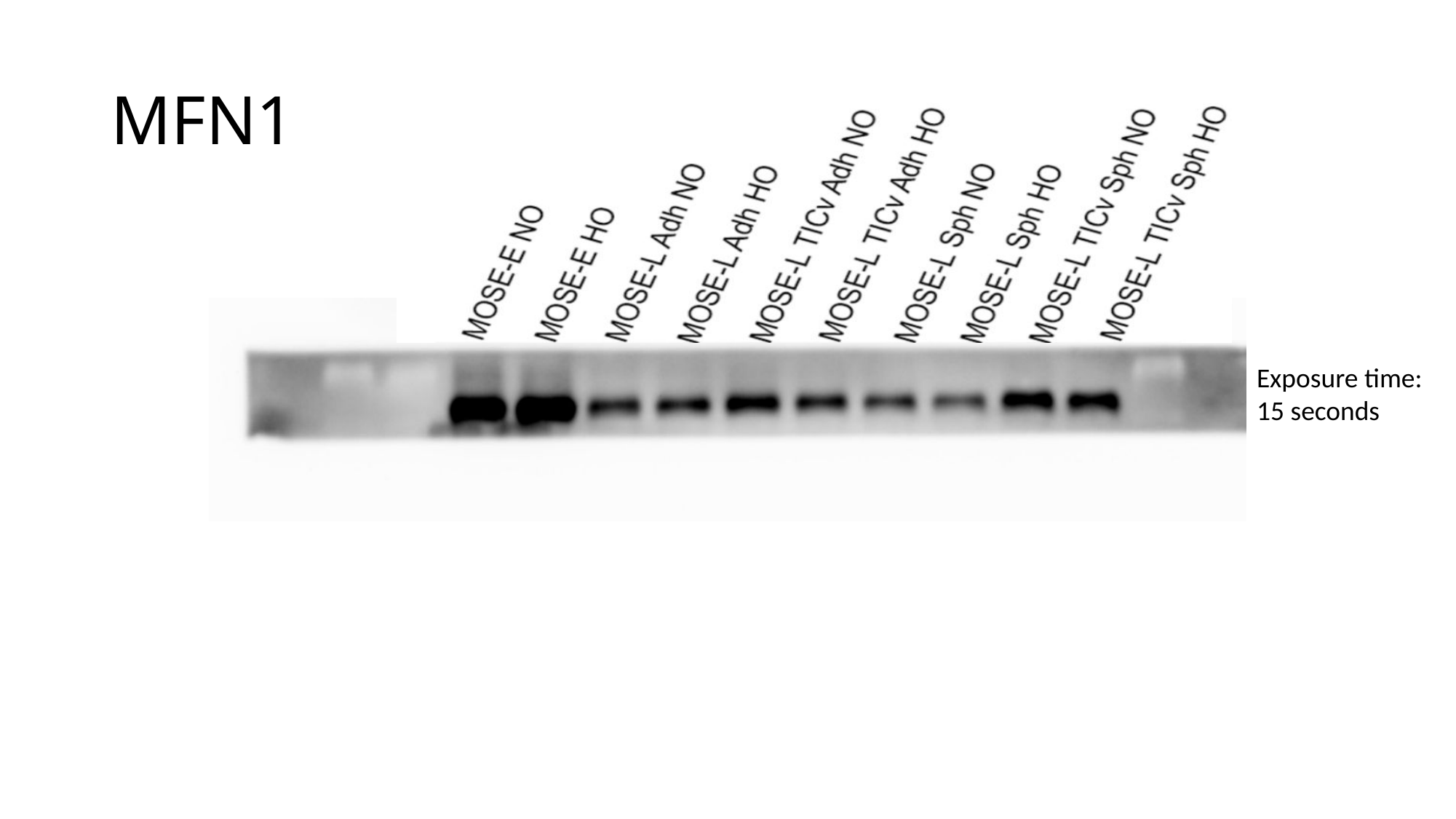

# MFN1
Exposure time: 15 seconds

## Slide 20
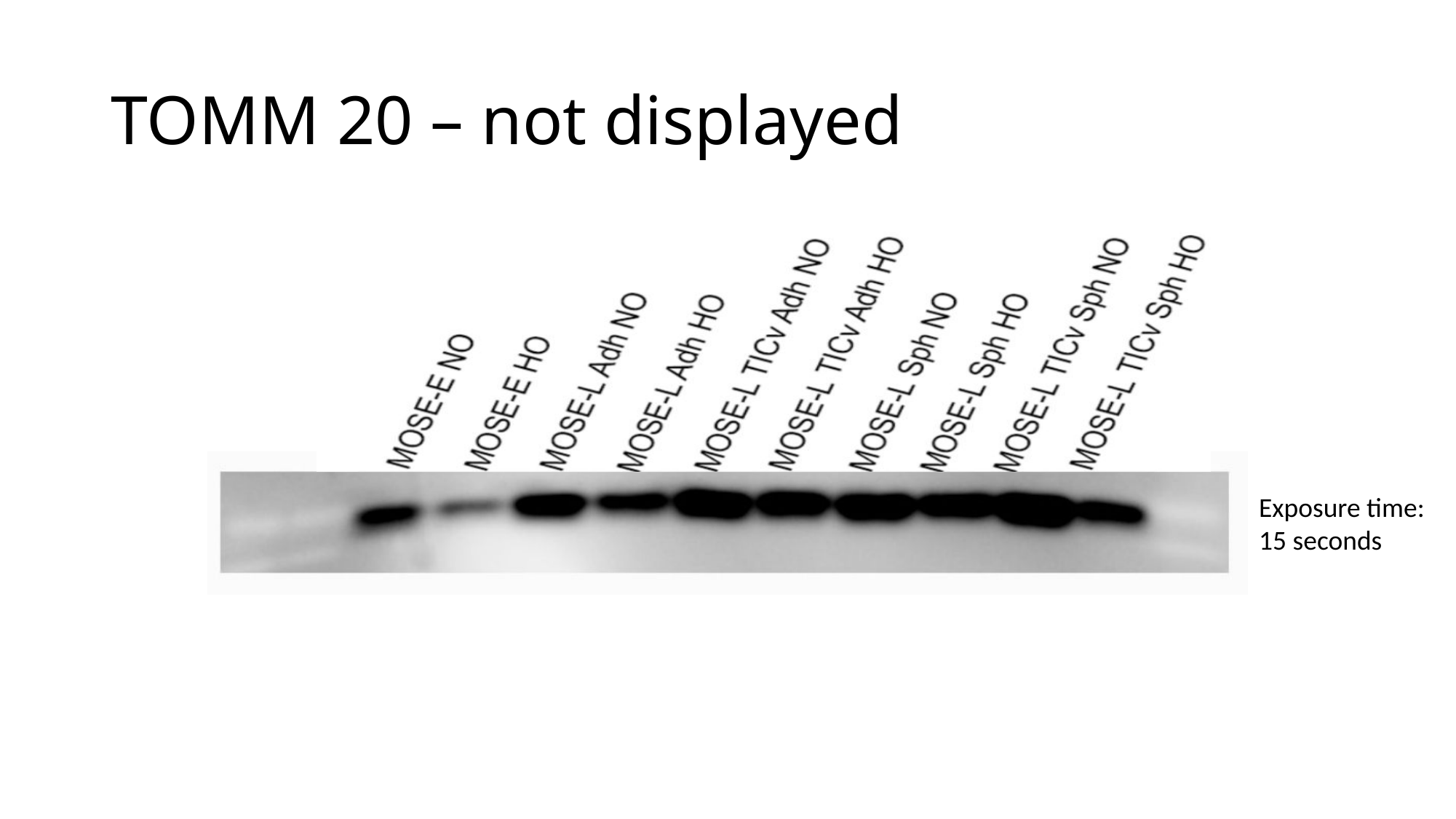

# TOMM 20 – not displayed
Exposure time: 15 seconds

## Slide 21
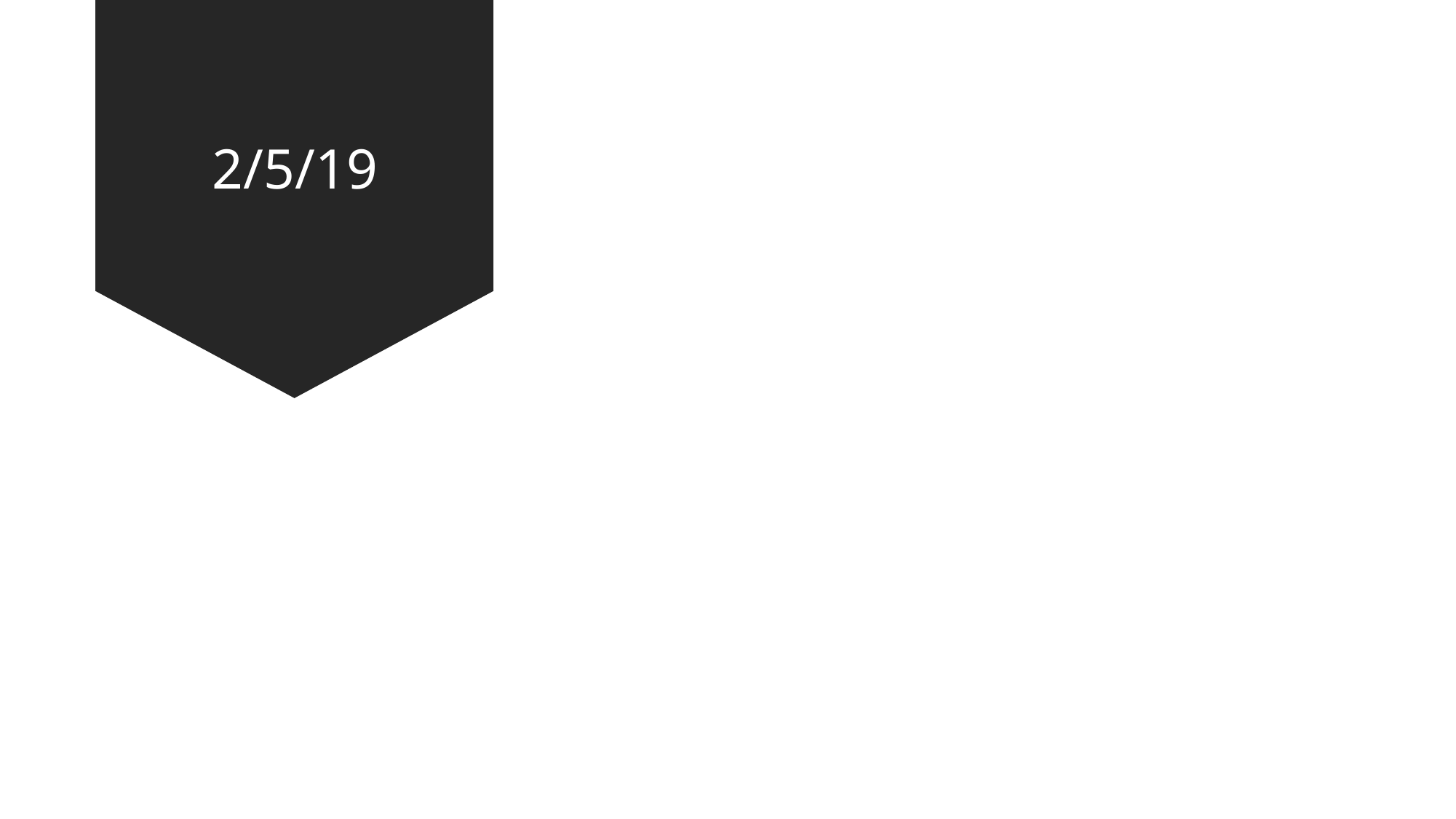

# 2/5/19

## Slide 22
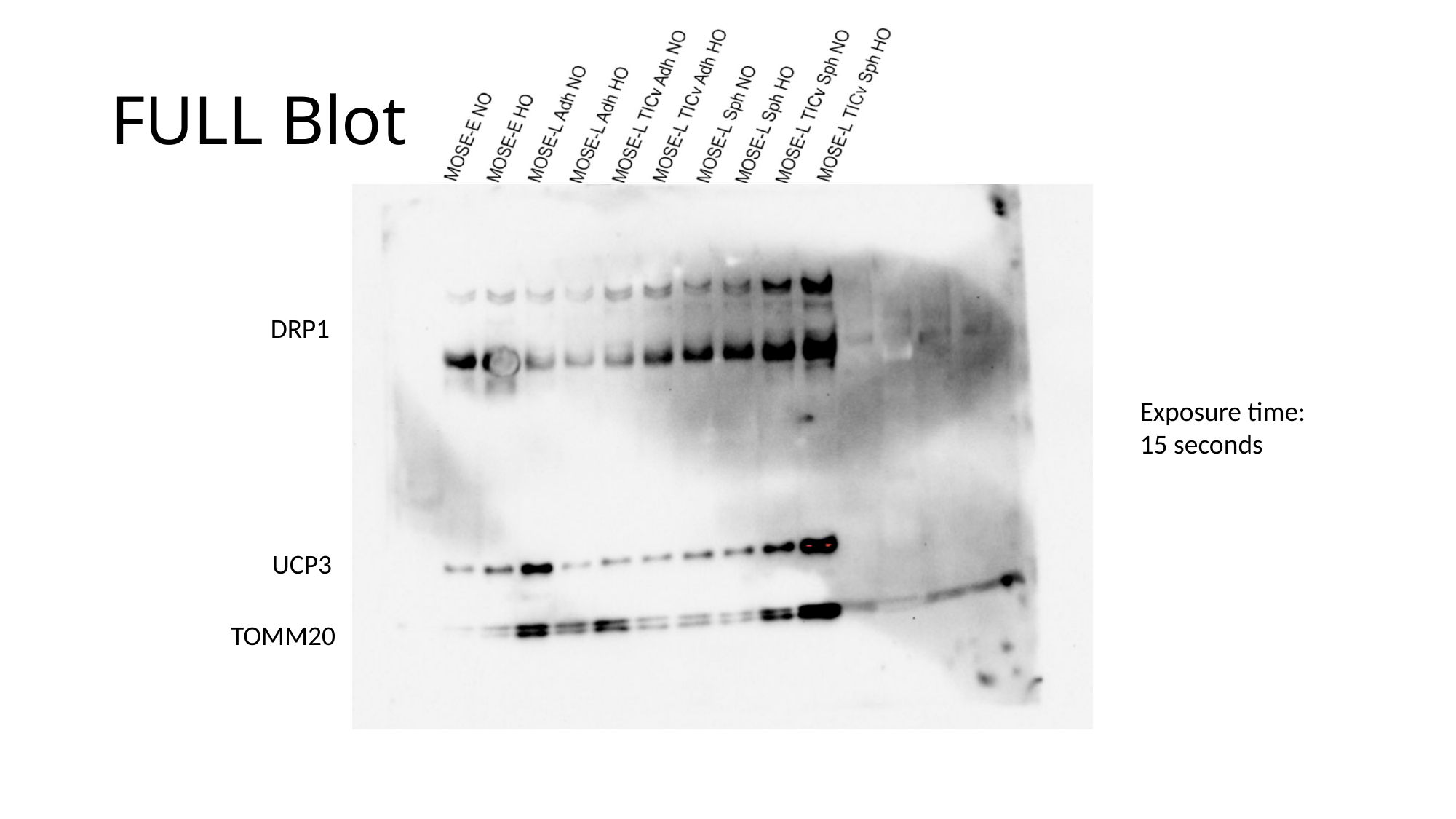

# FULL Blot
DRP1
Exposure time: 15 seconds
UCP3
TOMM20

## Slide 23
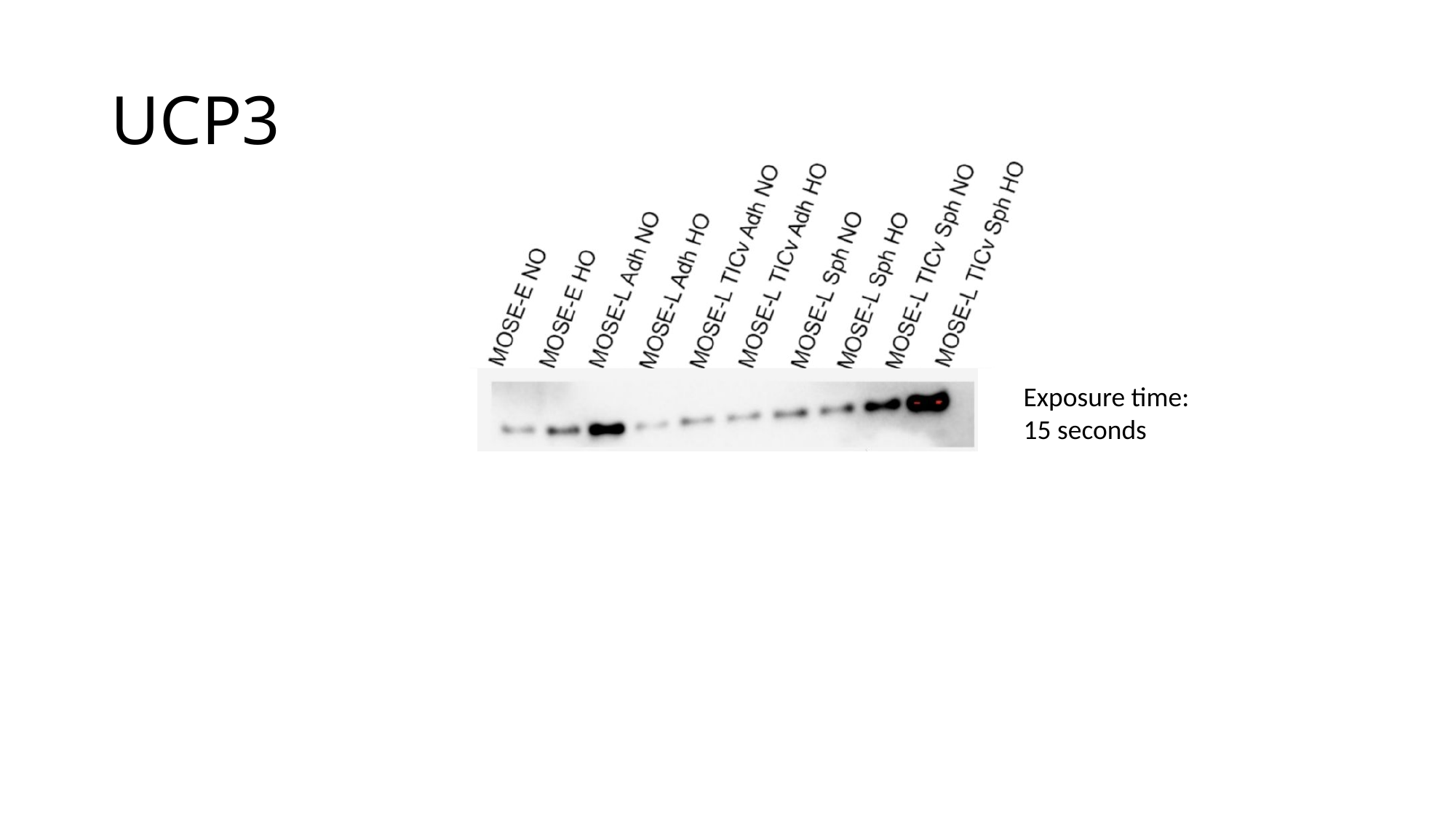

# UCP3
Exposure time: 15 seconds

## Slide 24
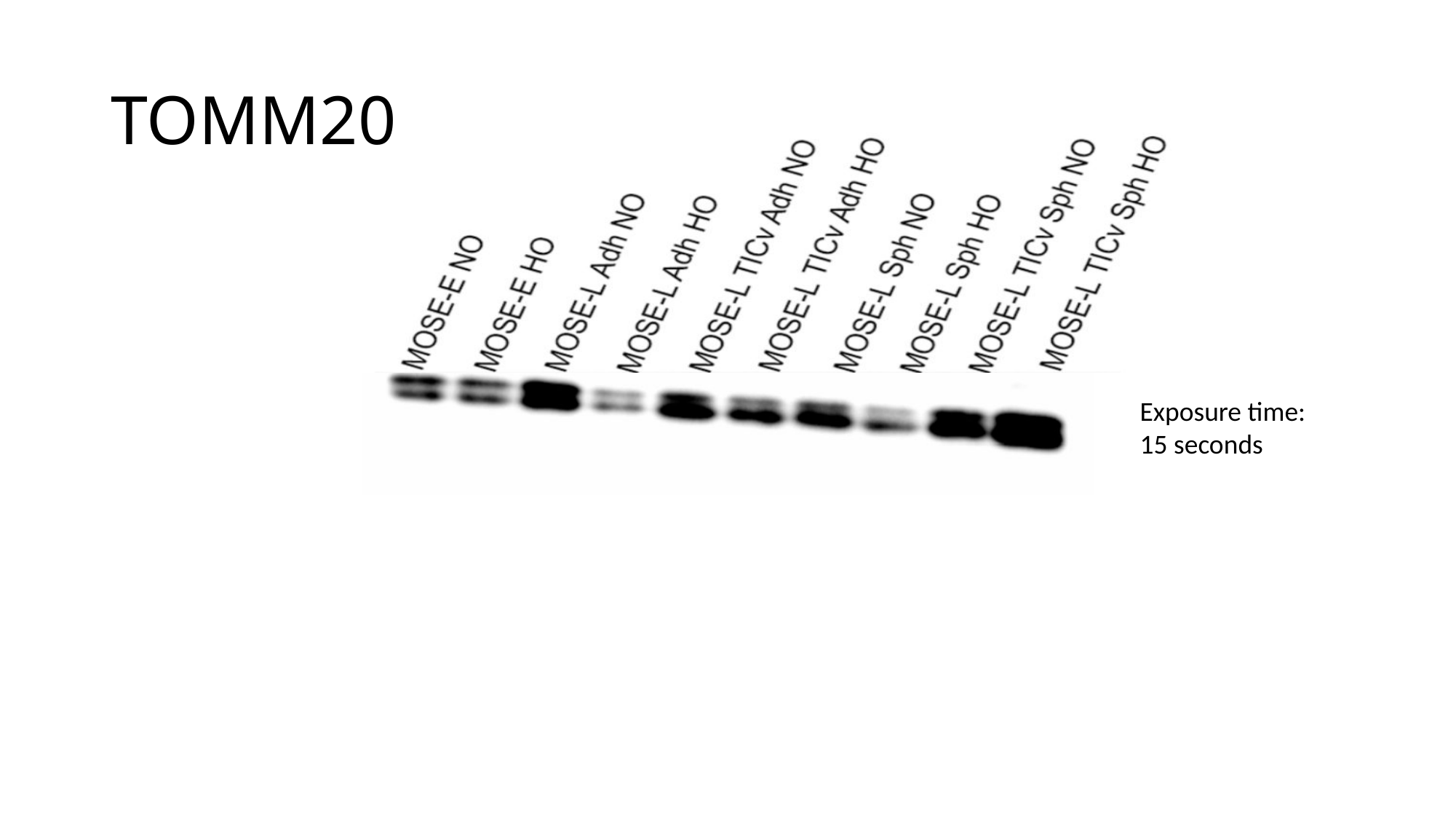

# TOMM20
Exposure time: 15 seconds

## Slide 25
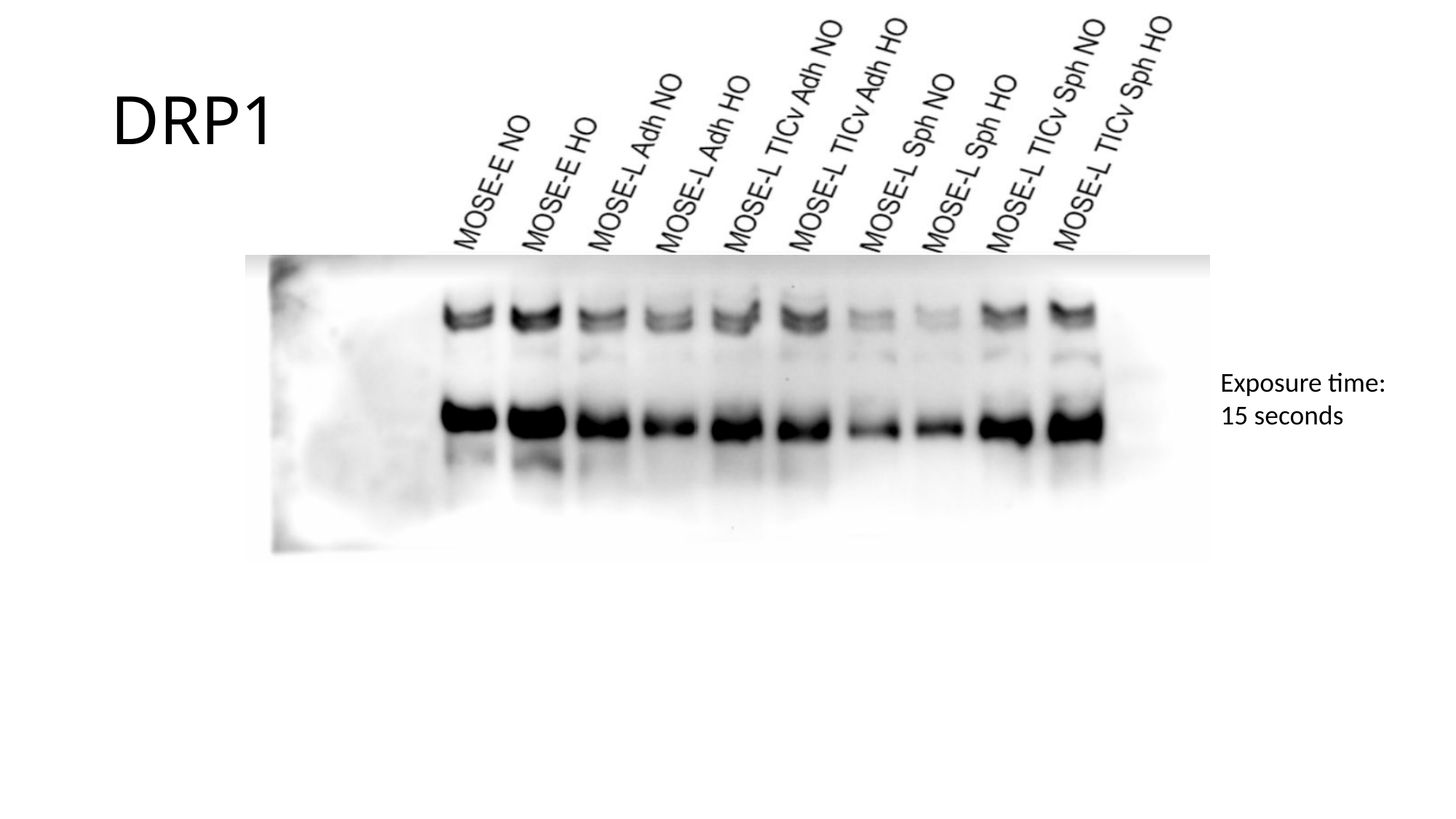

# DRP1
Exposure time: 15 seconds

## Slide 26
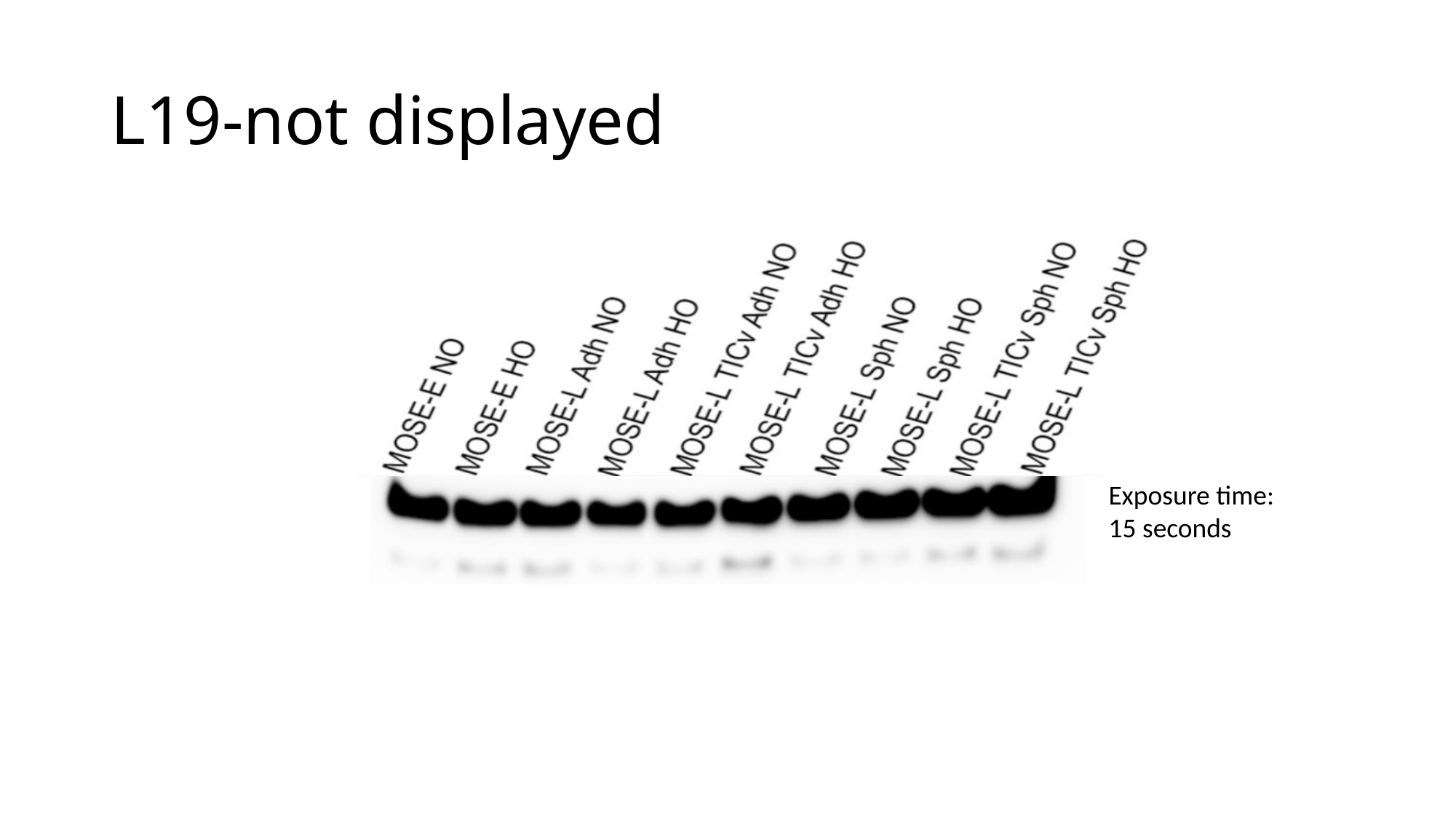

# L19-not displayed
Exposure time: 15 seconds

## Slide 27
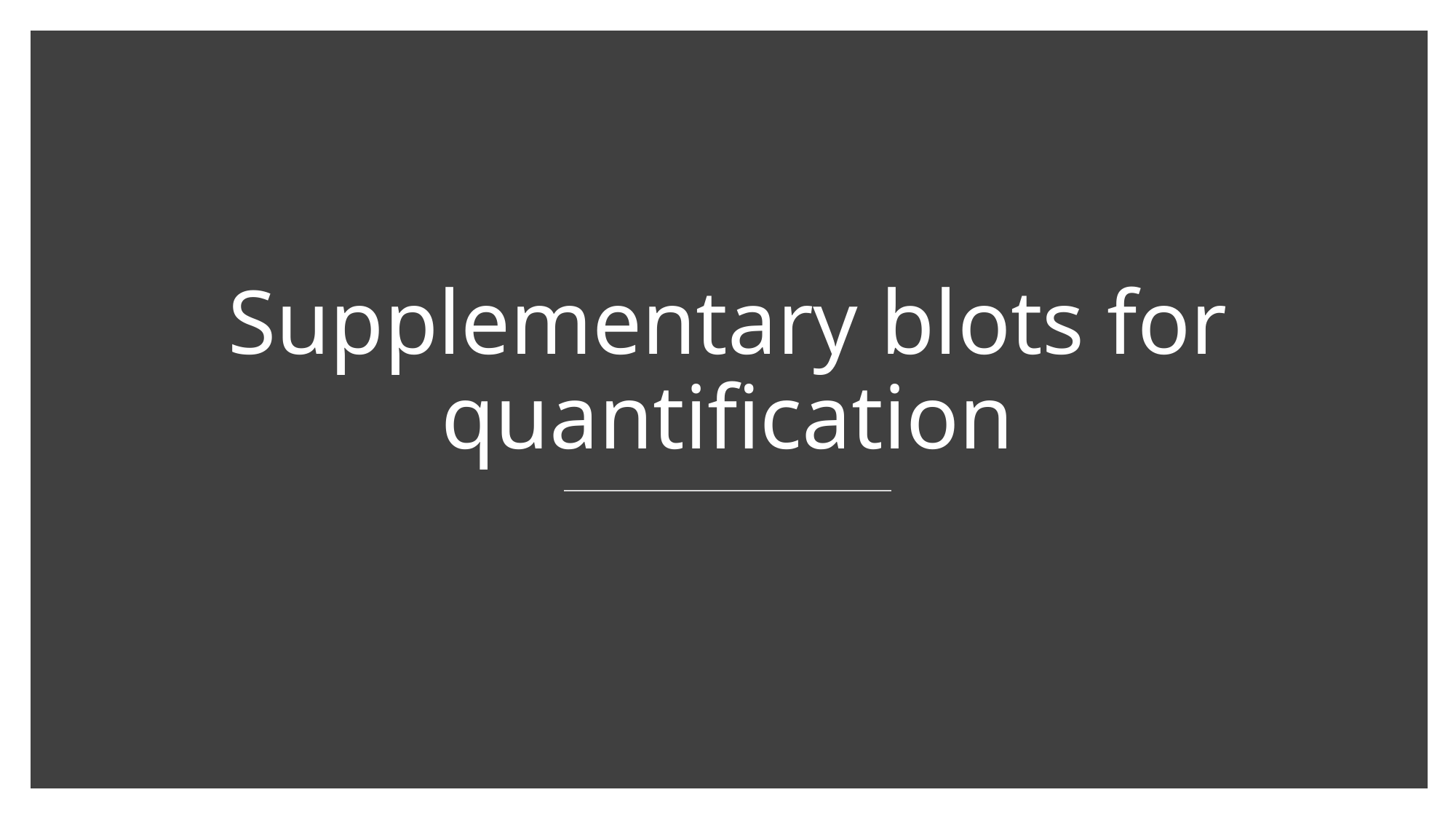

# Supplementary blots for quantification

## Slide 28
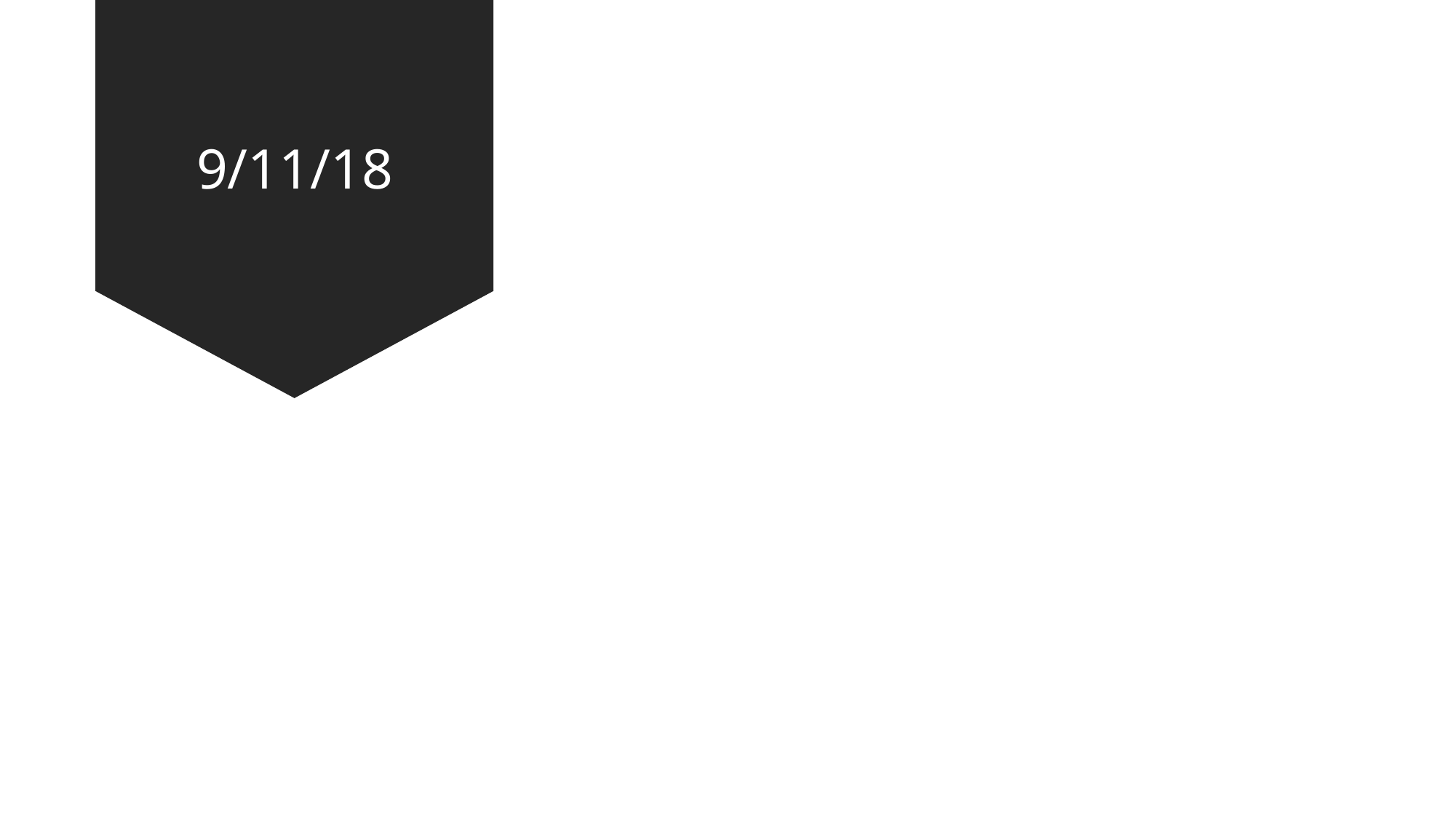

# 9/11/18

## Slide 29
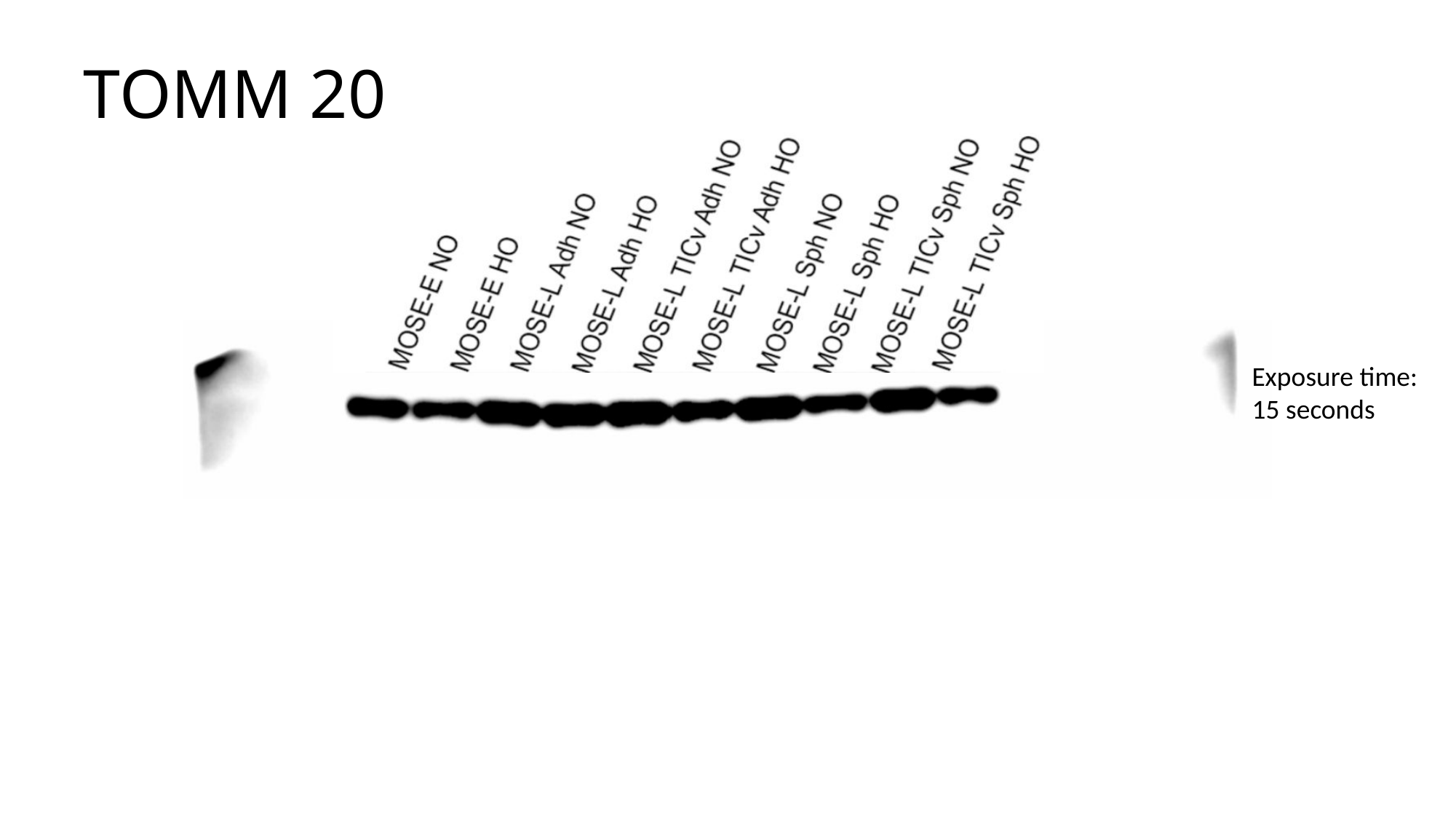

# TOMM 20
Exposure time: 15 seconds

## Slide 30
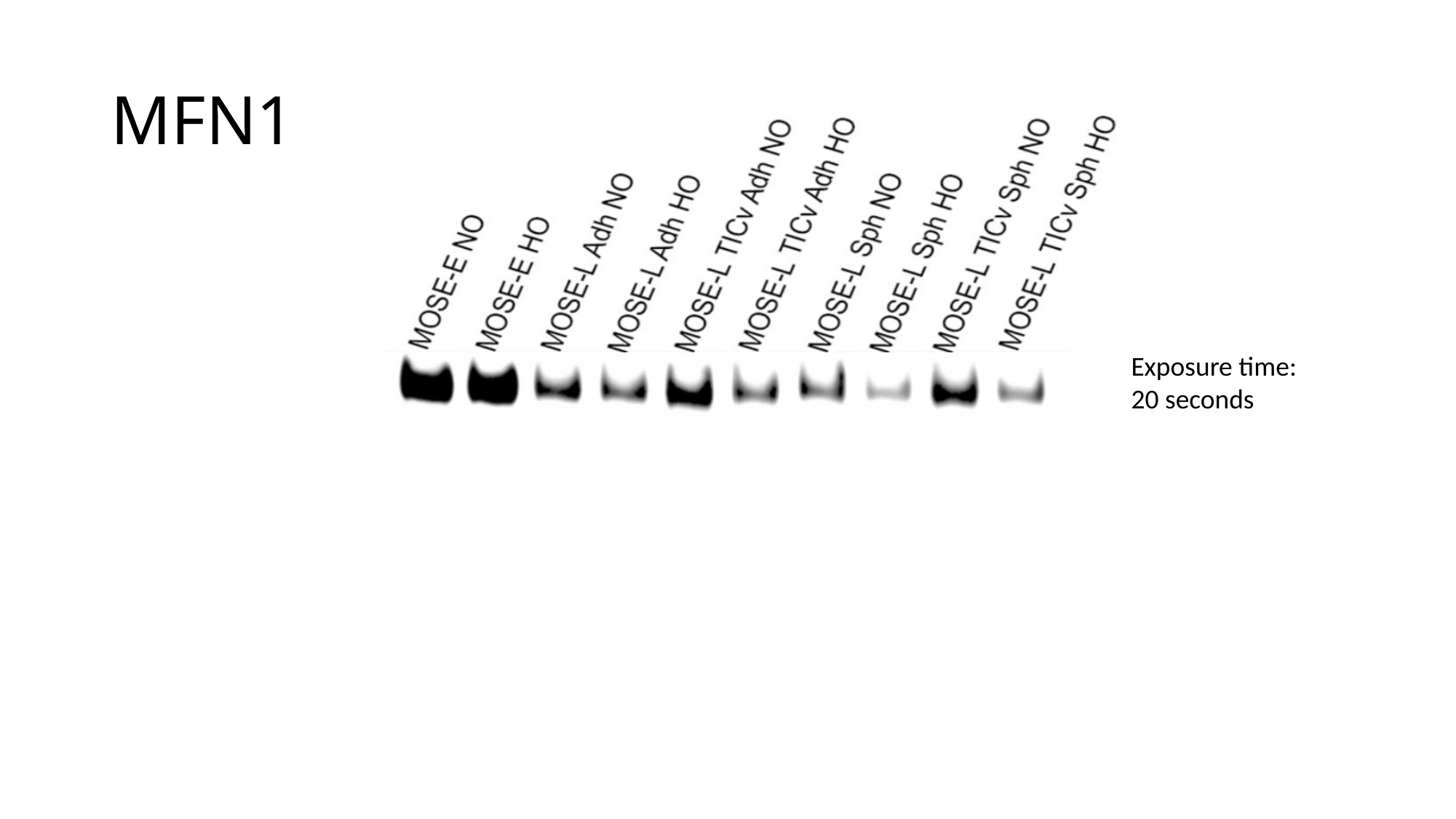

# MFN1
Exposure time: 20 seconds

## Slide 31
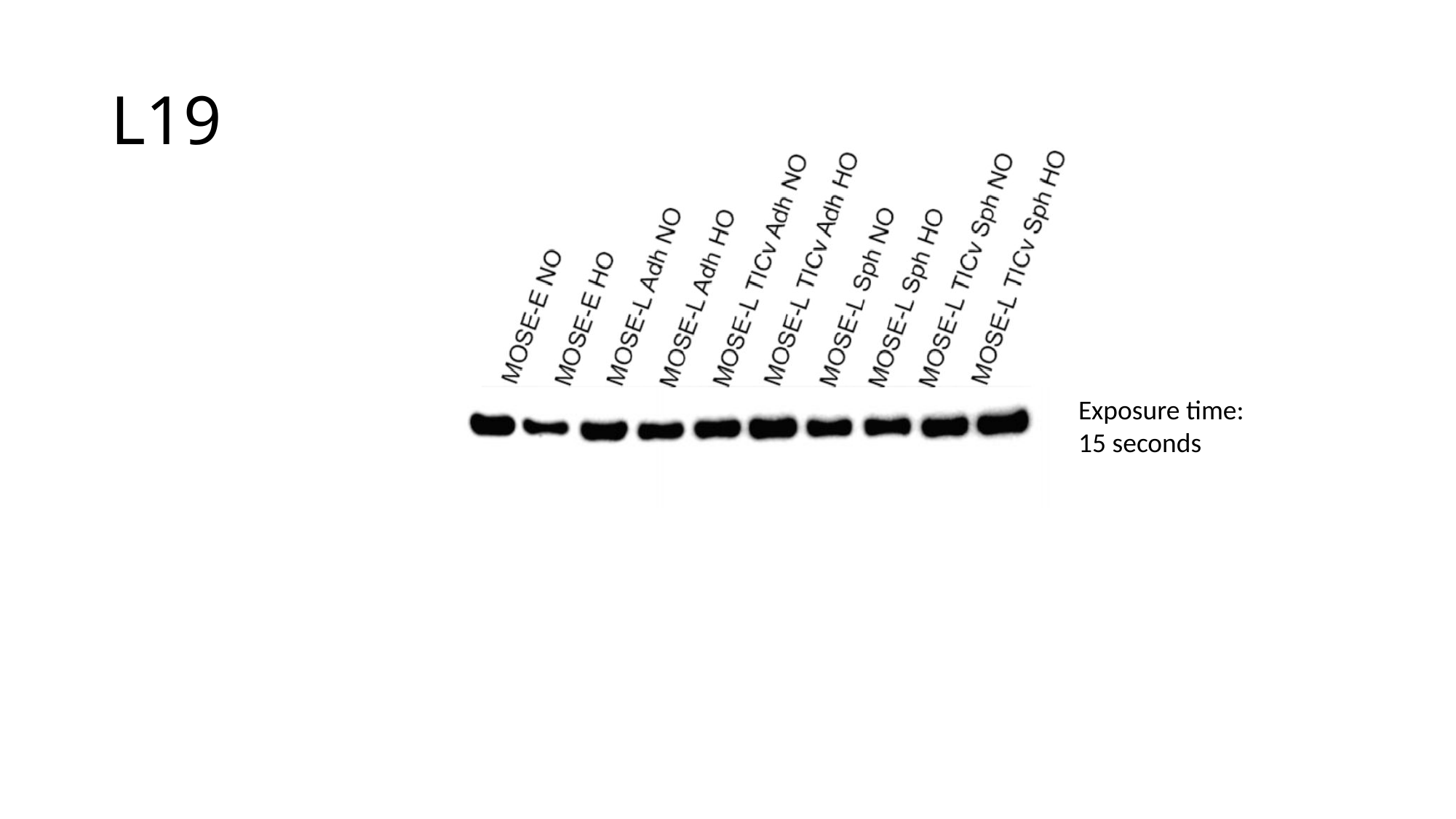

# L19
Exposure time: 15 seconds

## Slide 32
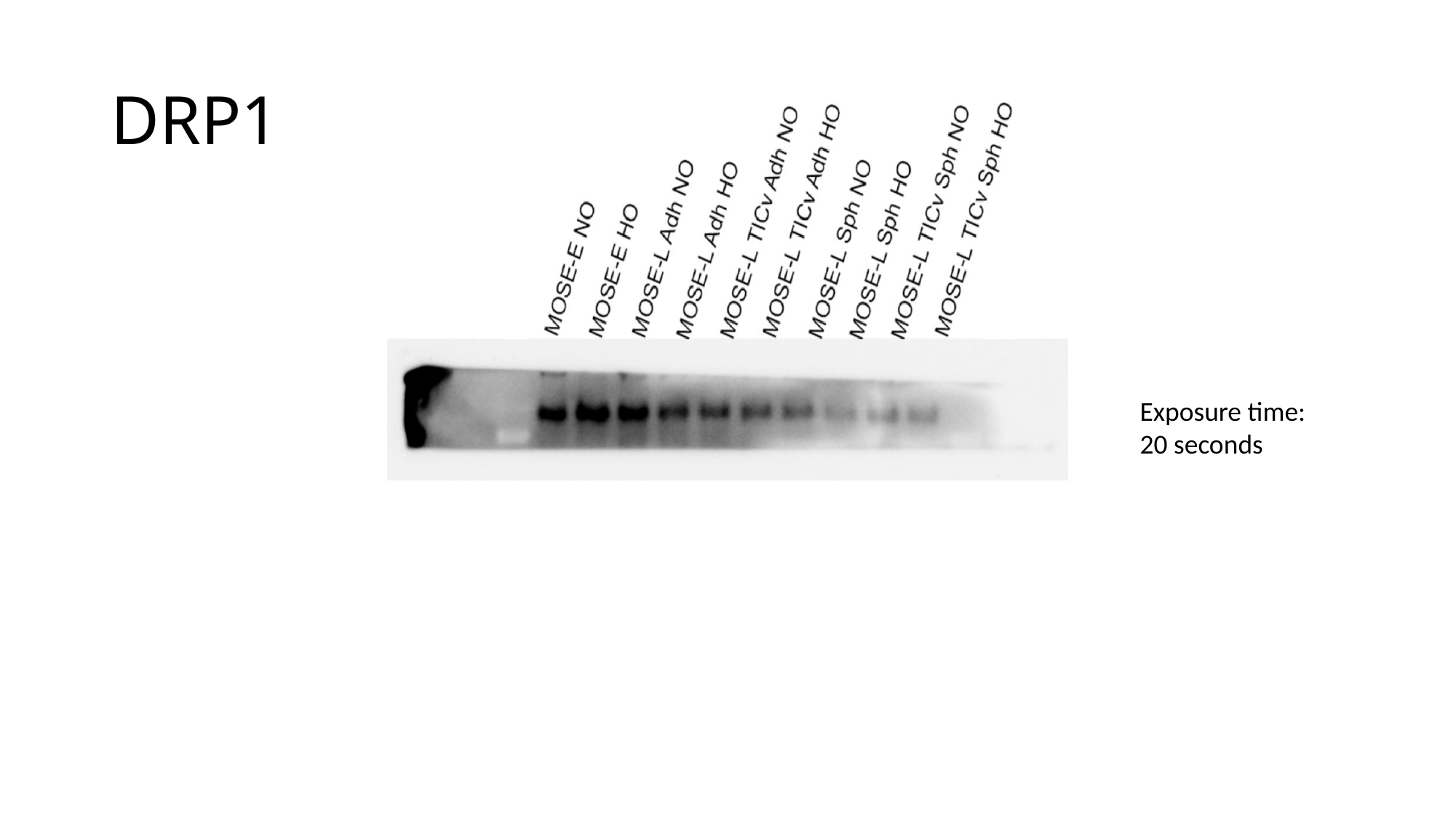

# DRP1
Exposure time: 20 seconds

## Slide 33
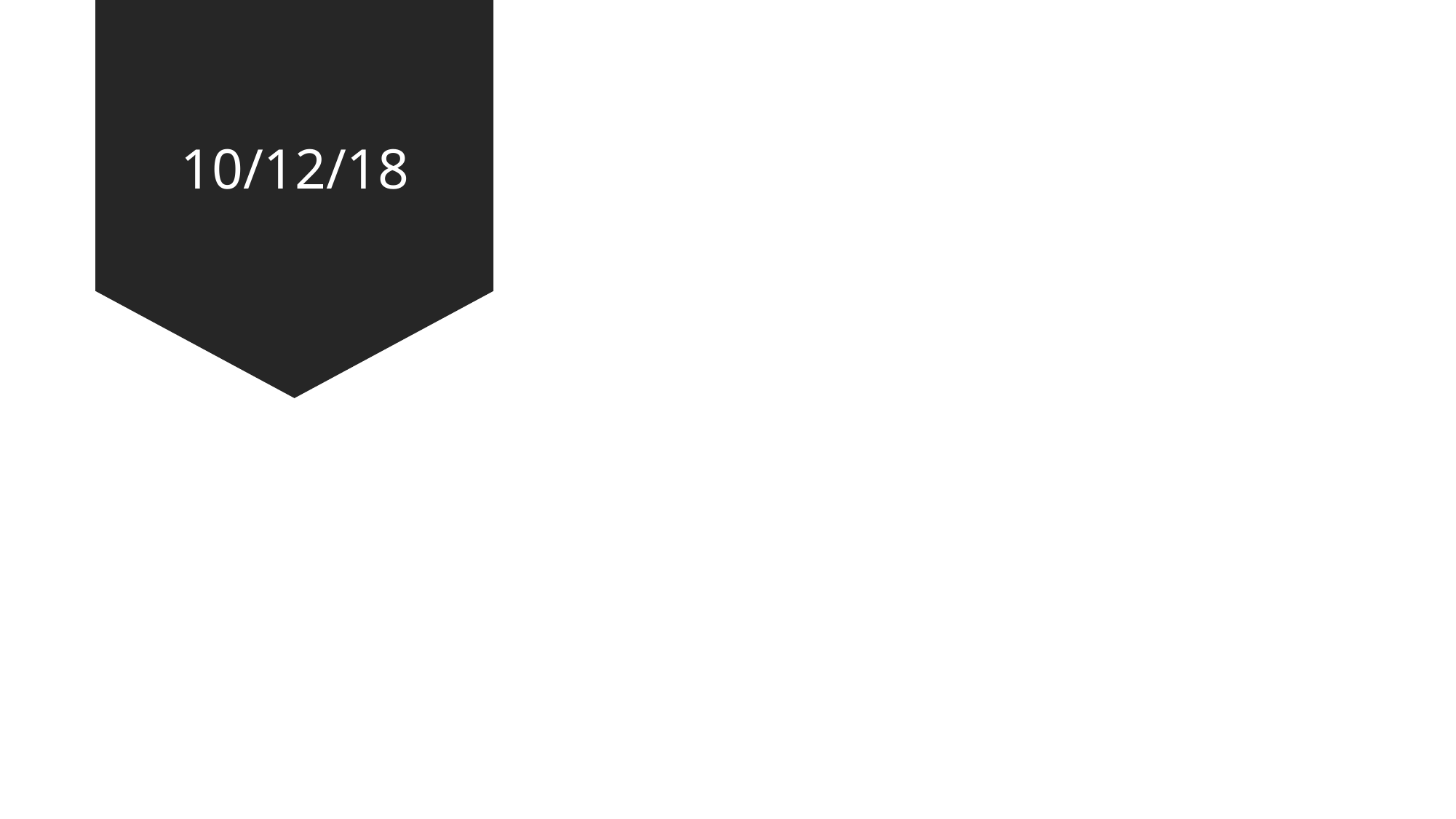

# 10/12/18

## Slide 34
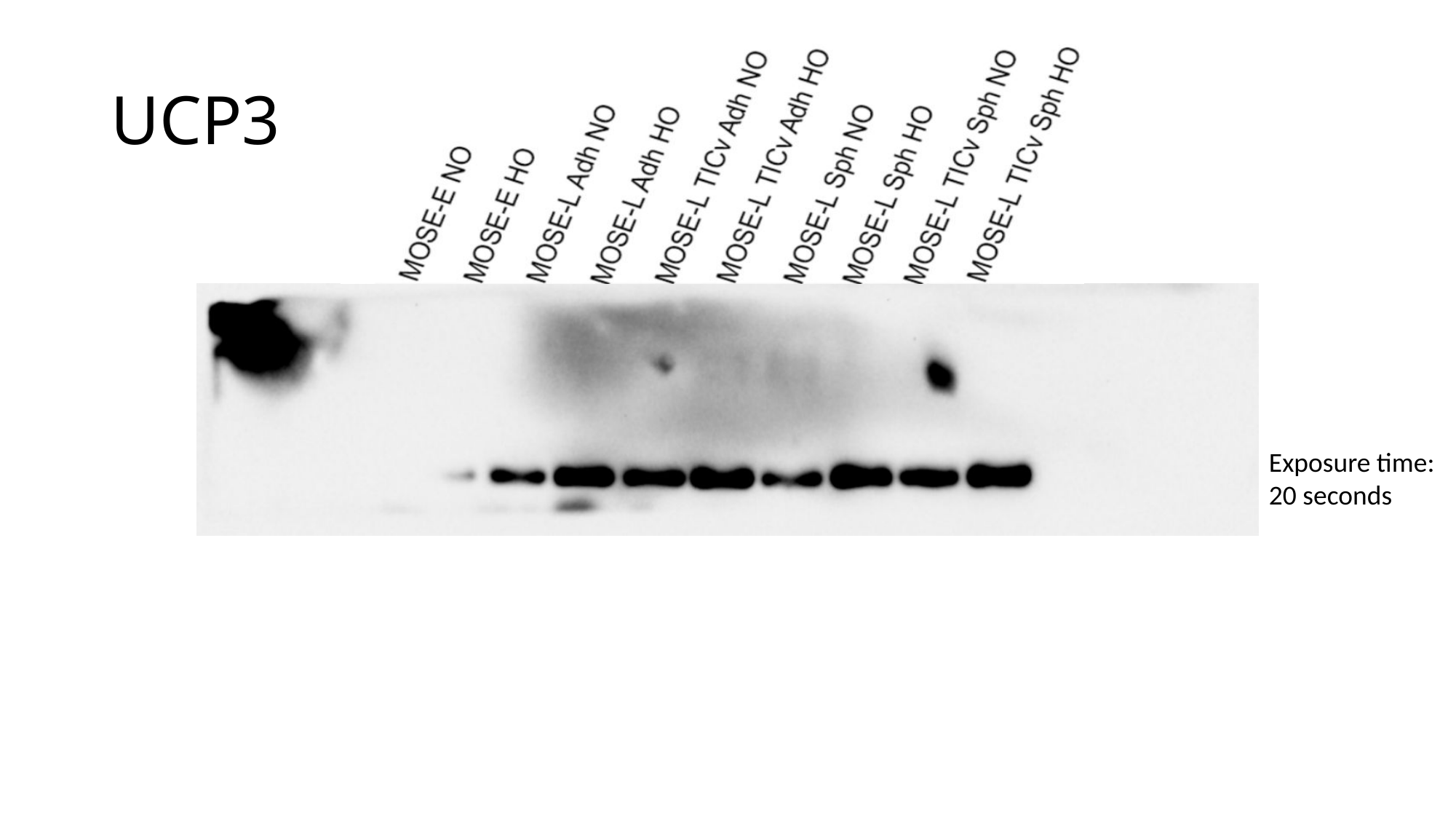

# UCP3
Exposure time: 20 seconds

## Slide 35
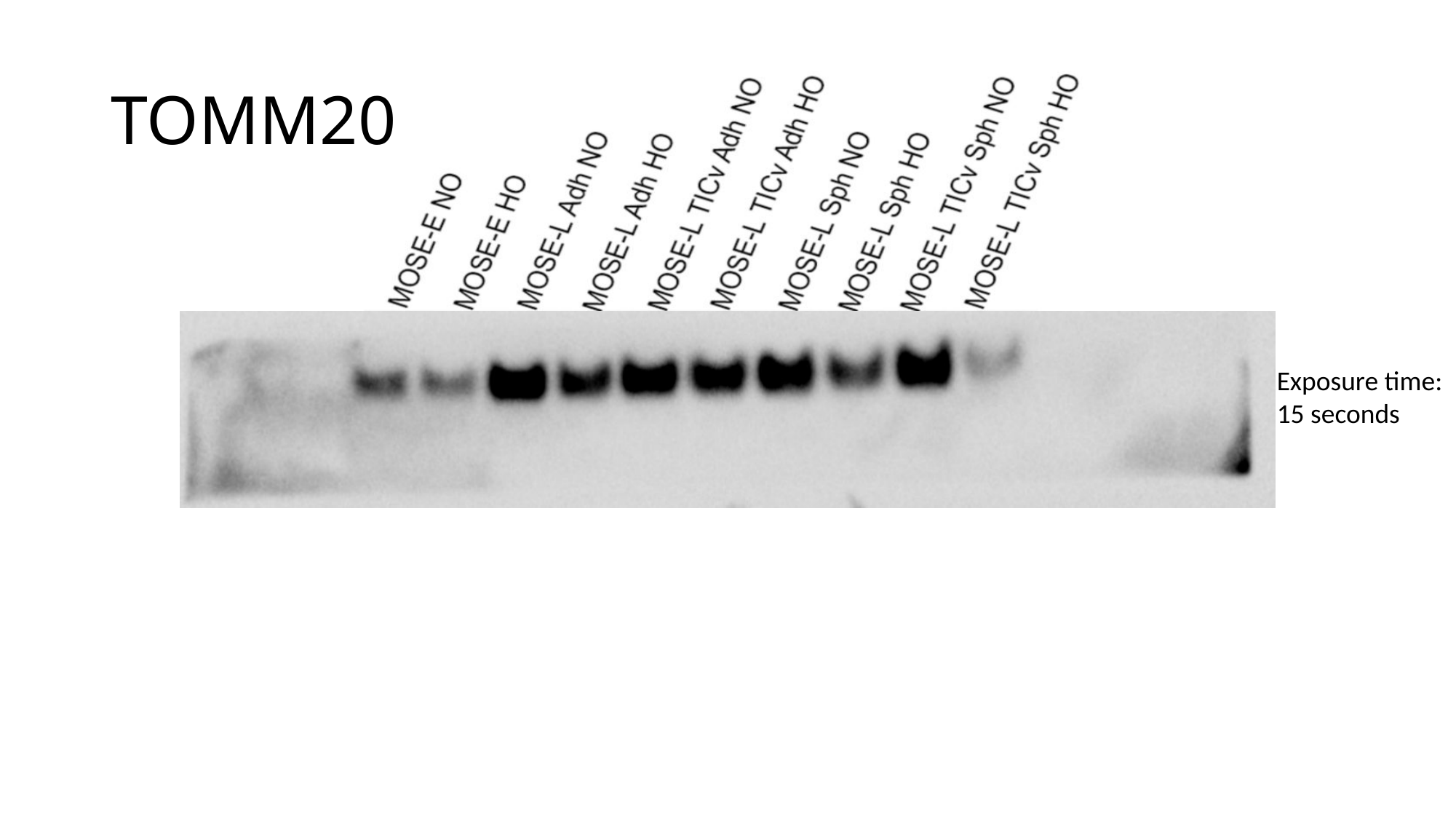

# TOMM20
Exposure time: 15 seconds

## Slide 36
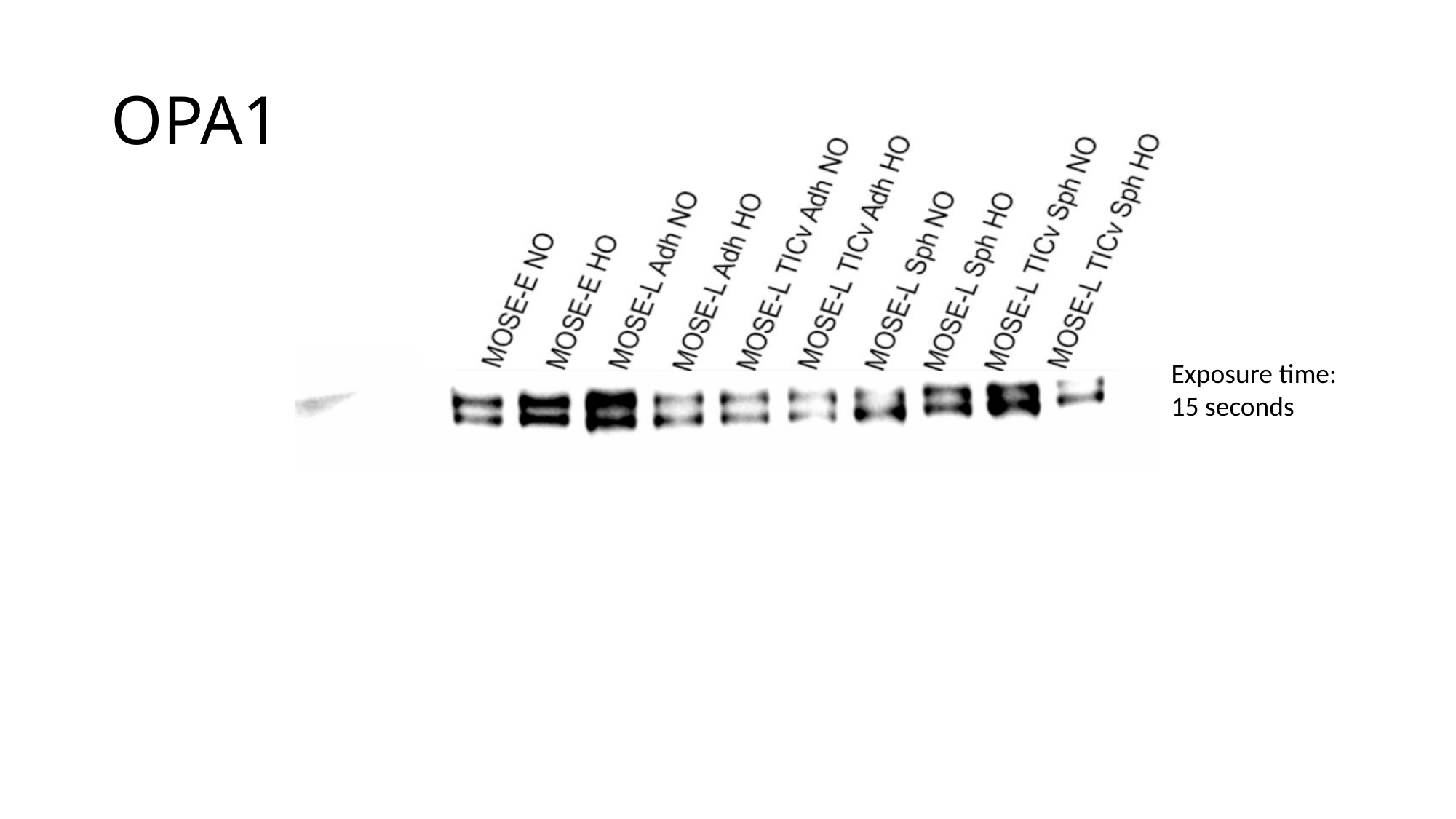

# OPA1
Exposure time: 15 seconds

## Slide 37
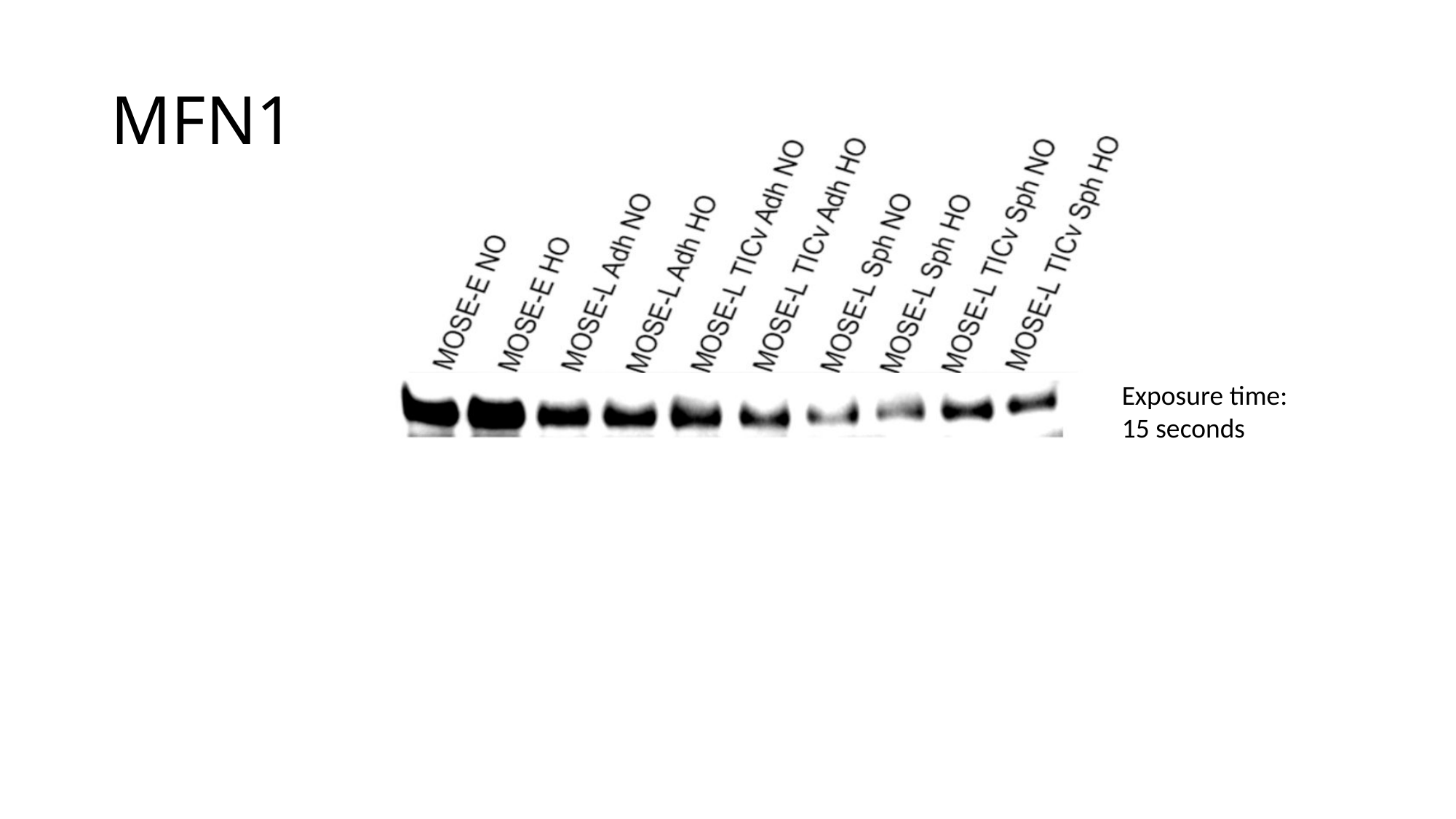

# MFN1
Exposure time: 15 seconds

## Slide 38
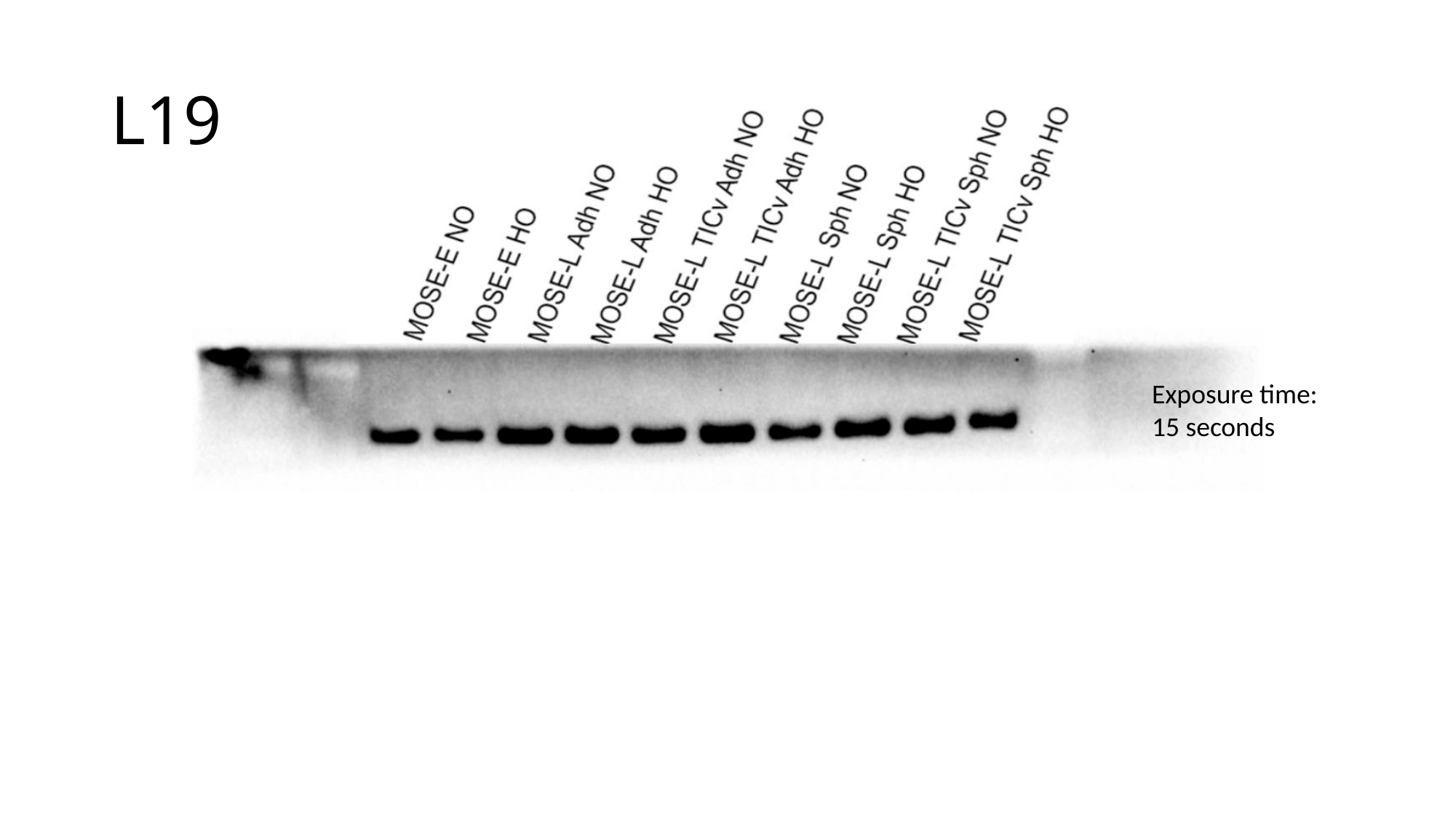

# L19
Exposure time: 15 seconds

## Slide 39
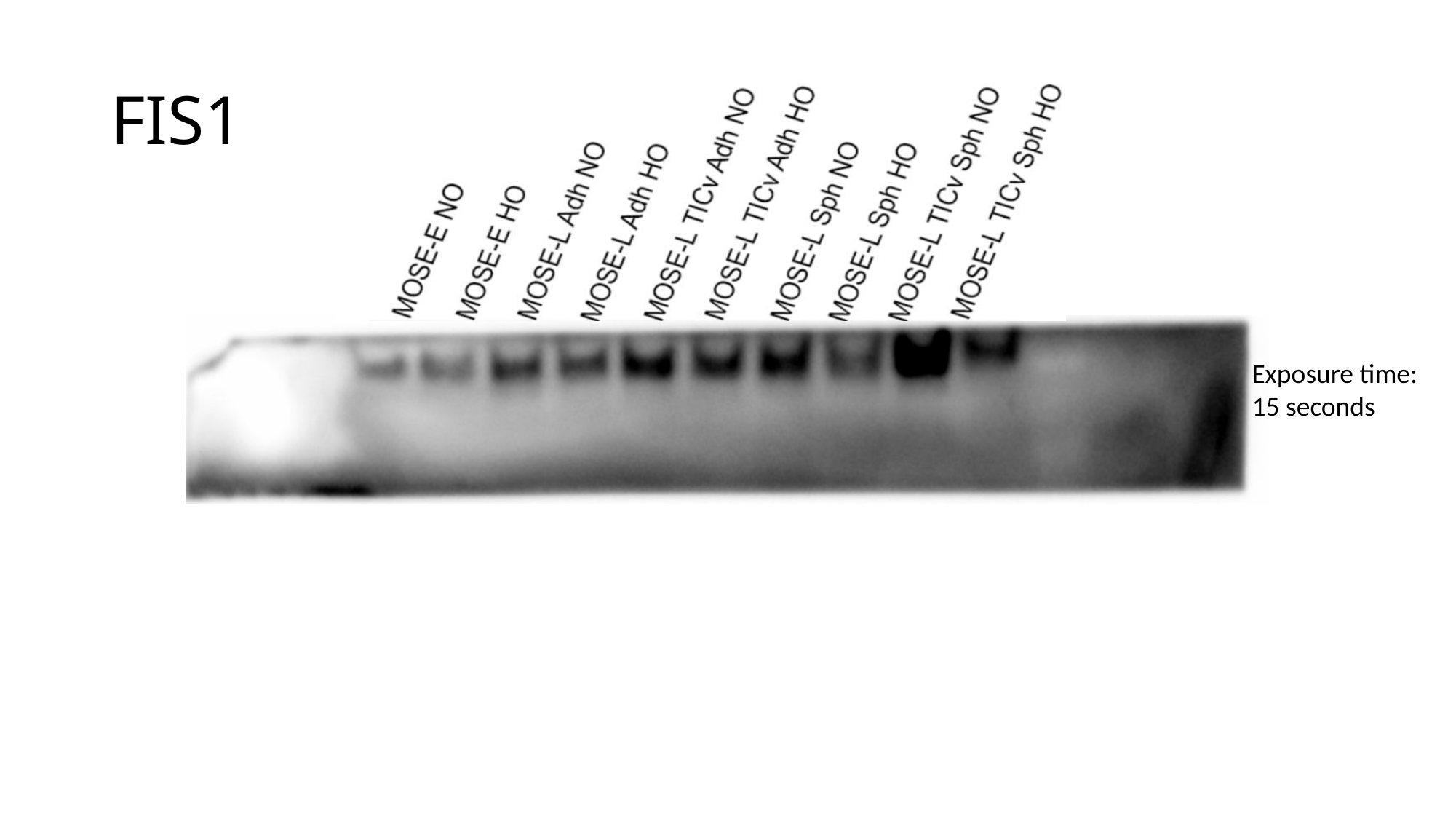

# FIS1
Exposure time: 15 seconds

## Slide 40
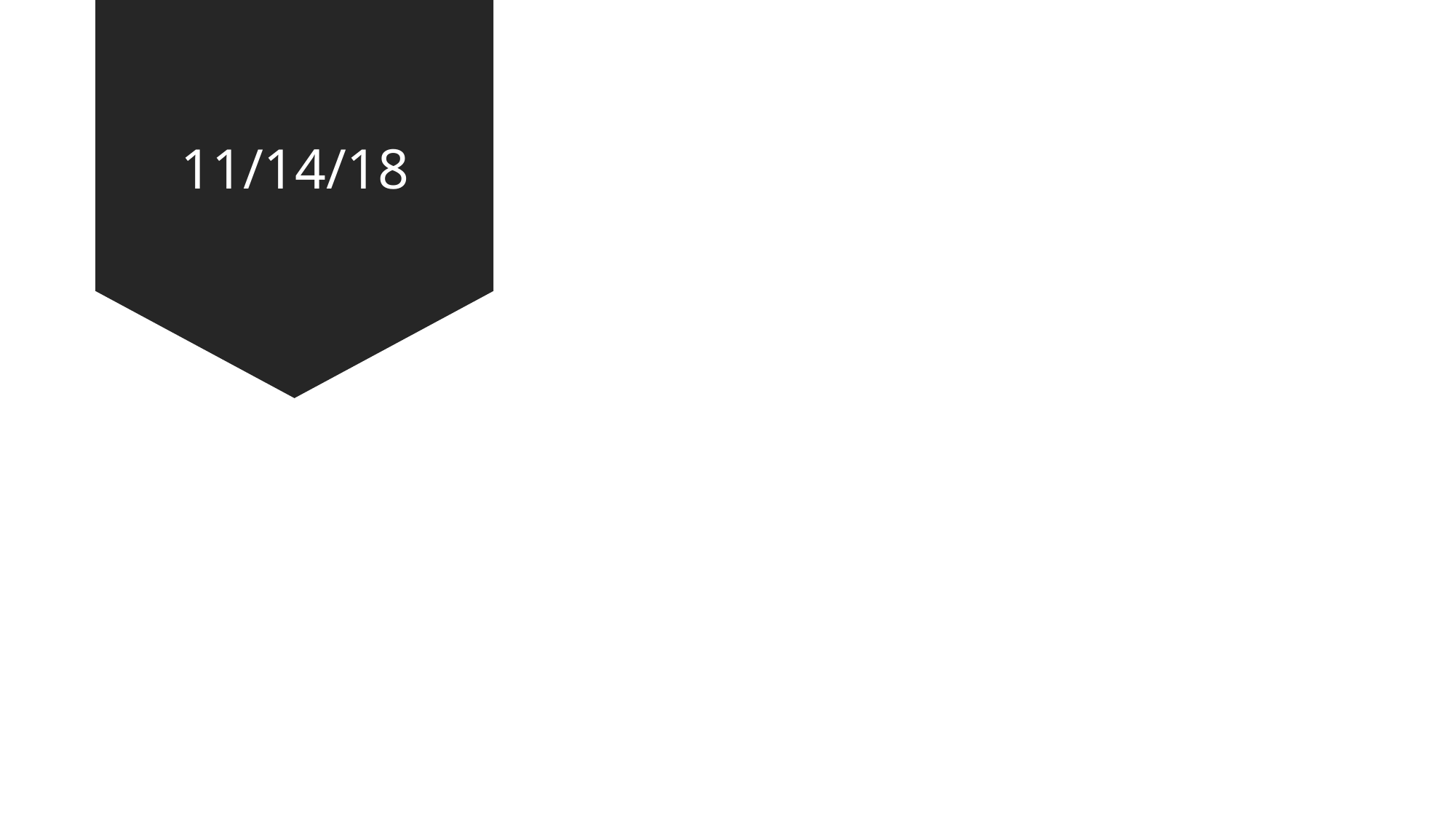

# 11/14/18

## Slide 41
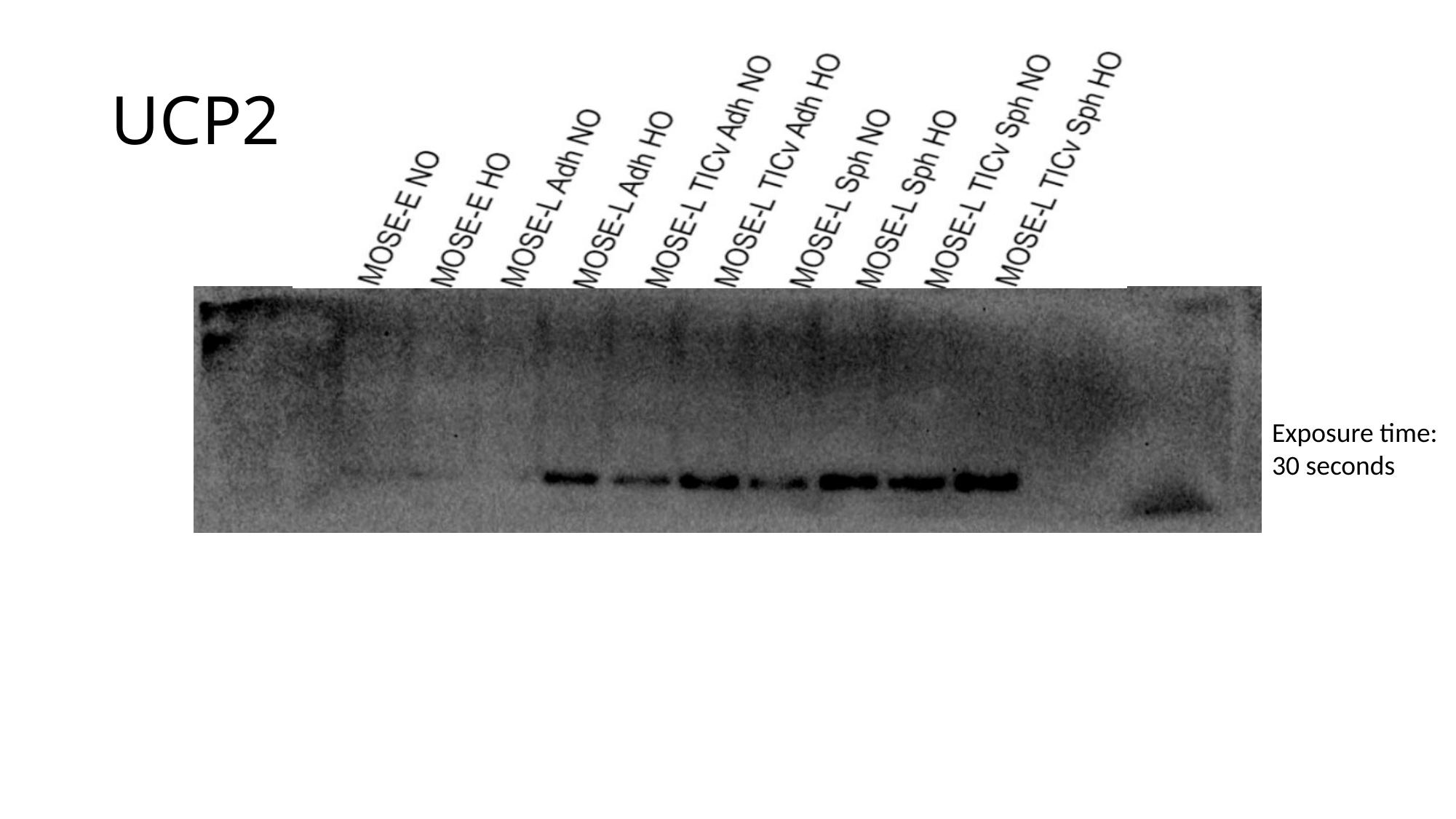

# UCP2
Exposure time: 30 seconds

## Slide 42
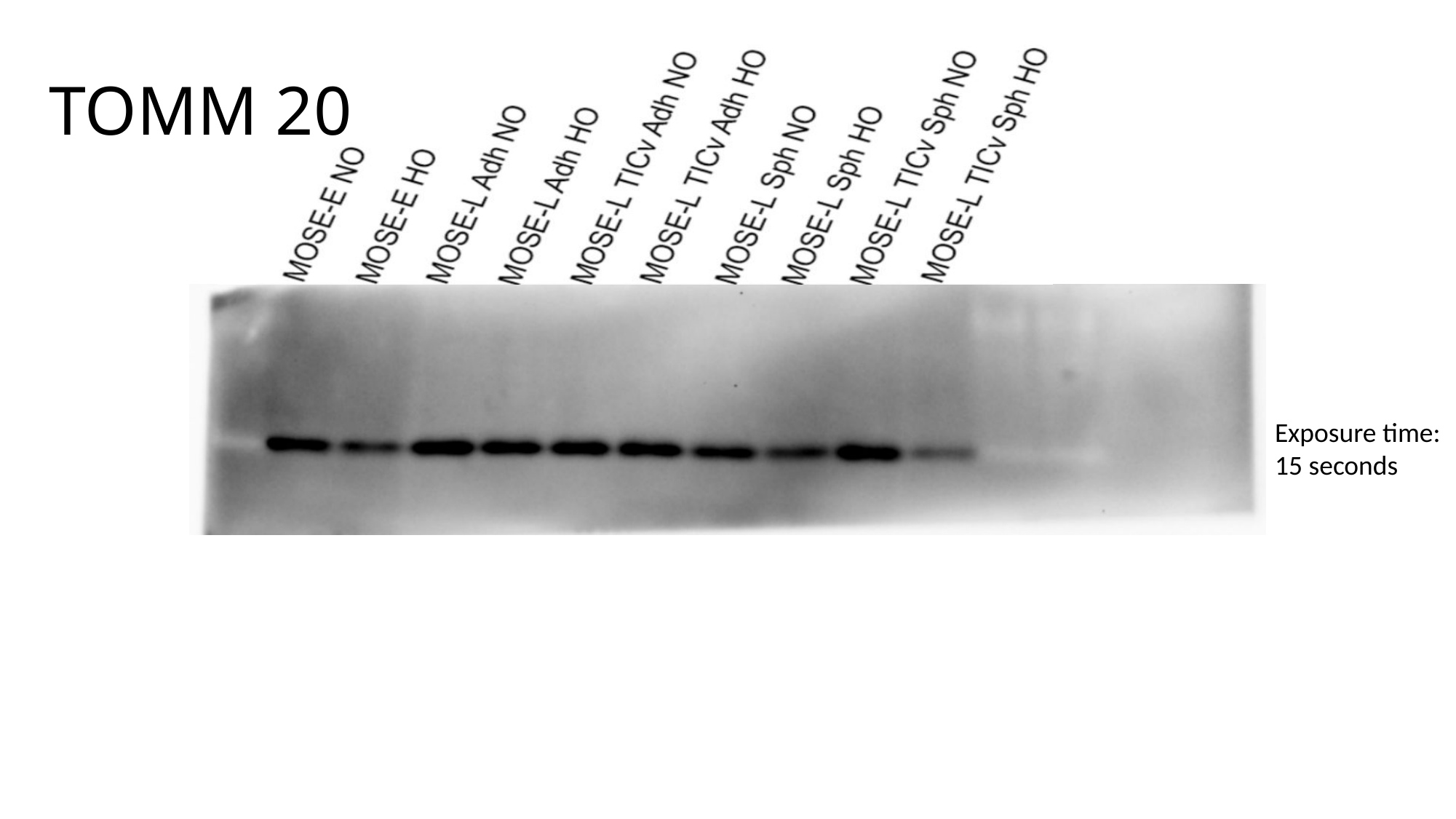

# TOMM 20
Exposure time: 15 seconds

## Slide 43
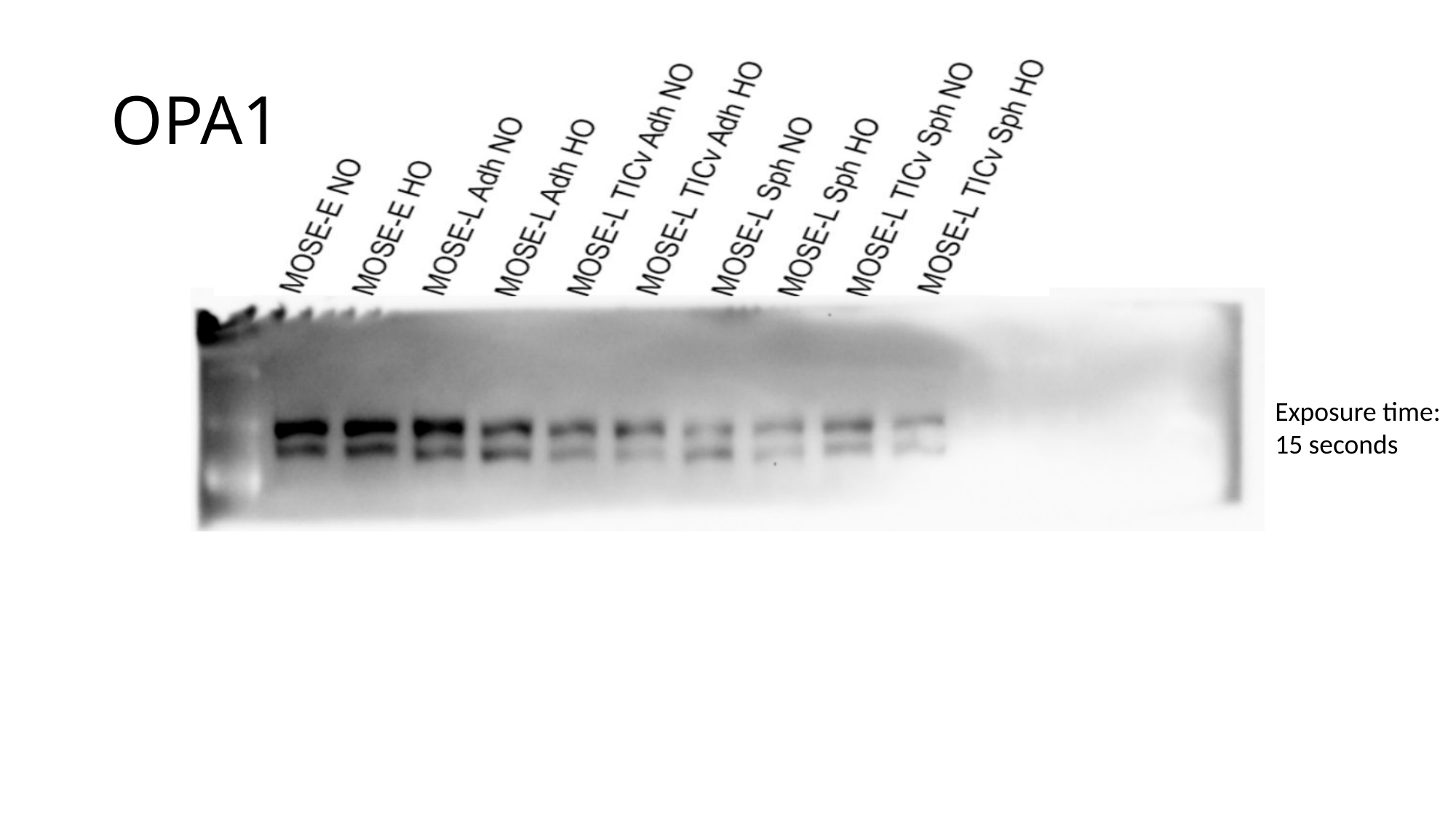

# OPA1
Exposure time: 15 seconds

## Slide 44
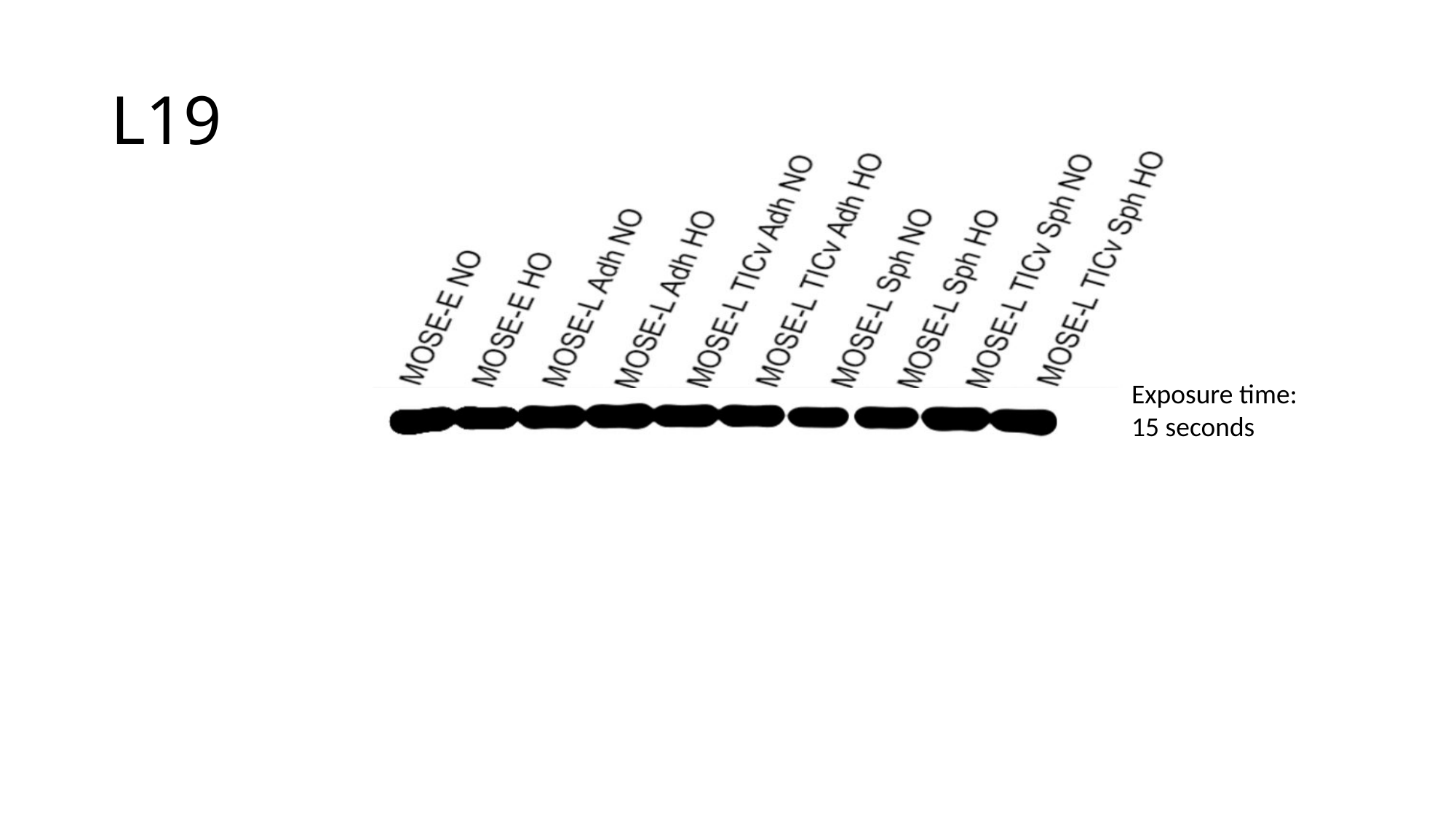

# L19
Exposure time: 15 seconds

## Slide 45
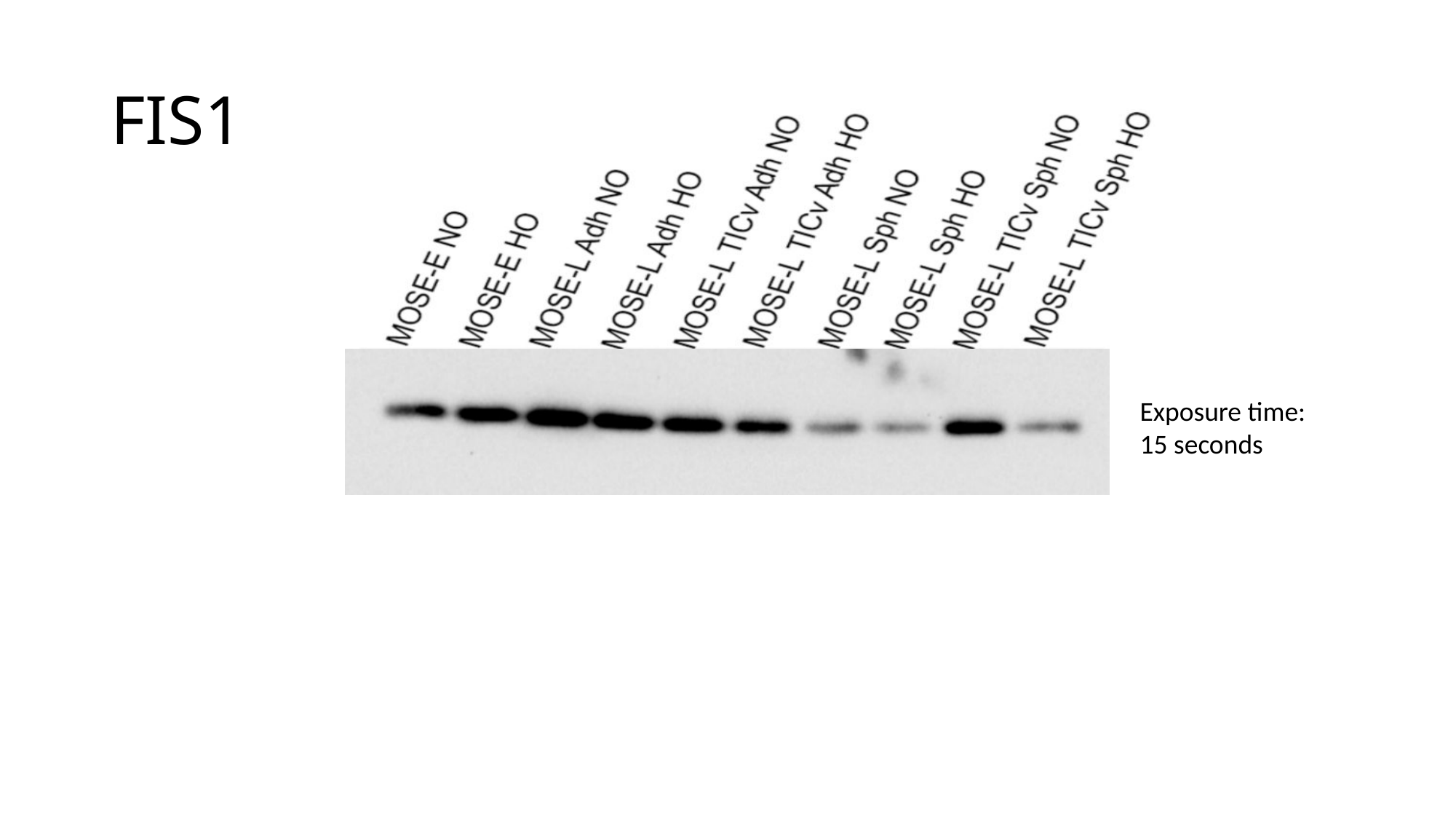

# FIS1
Exposure time: 15 seconds

## Slide 46
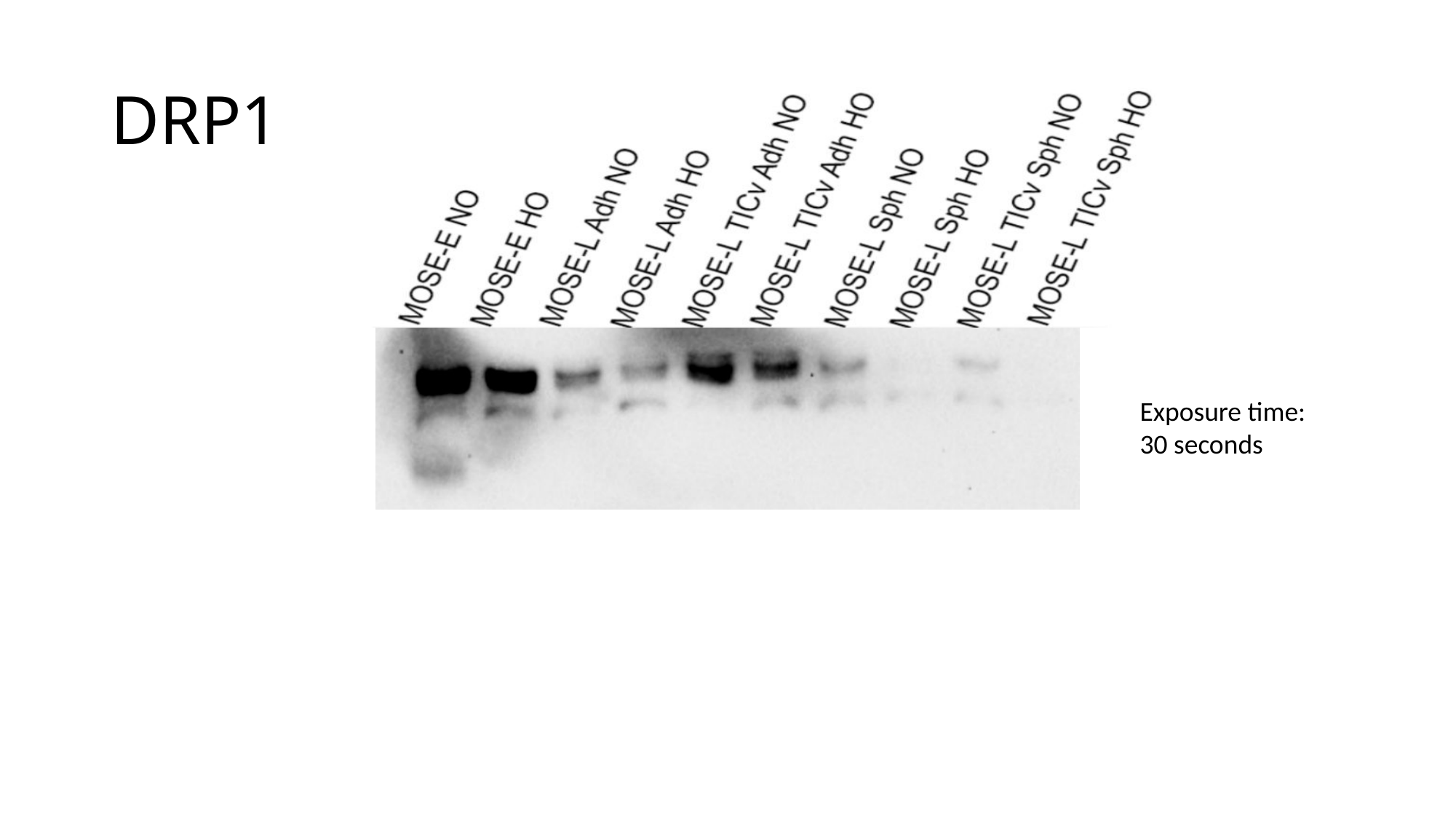

# DRP1
Exposure time: 30 seconds

## Slide 47
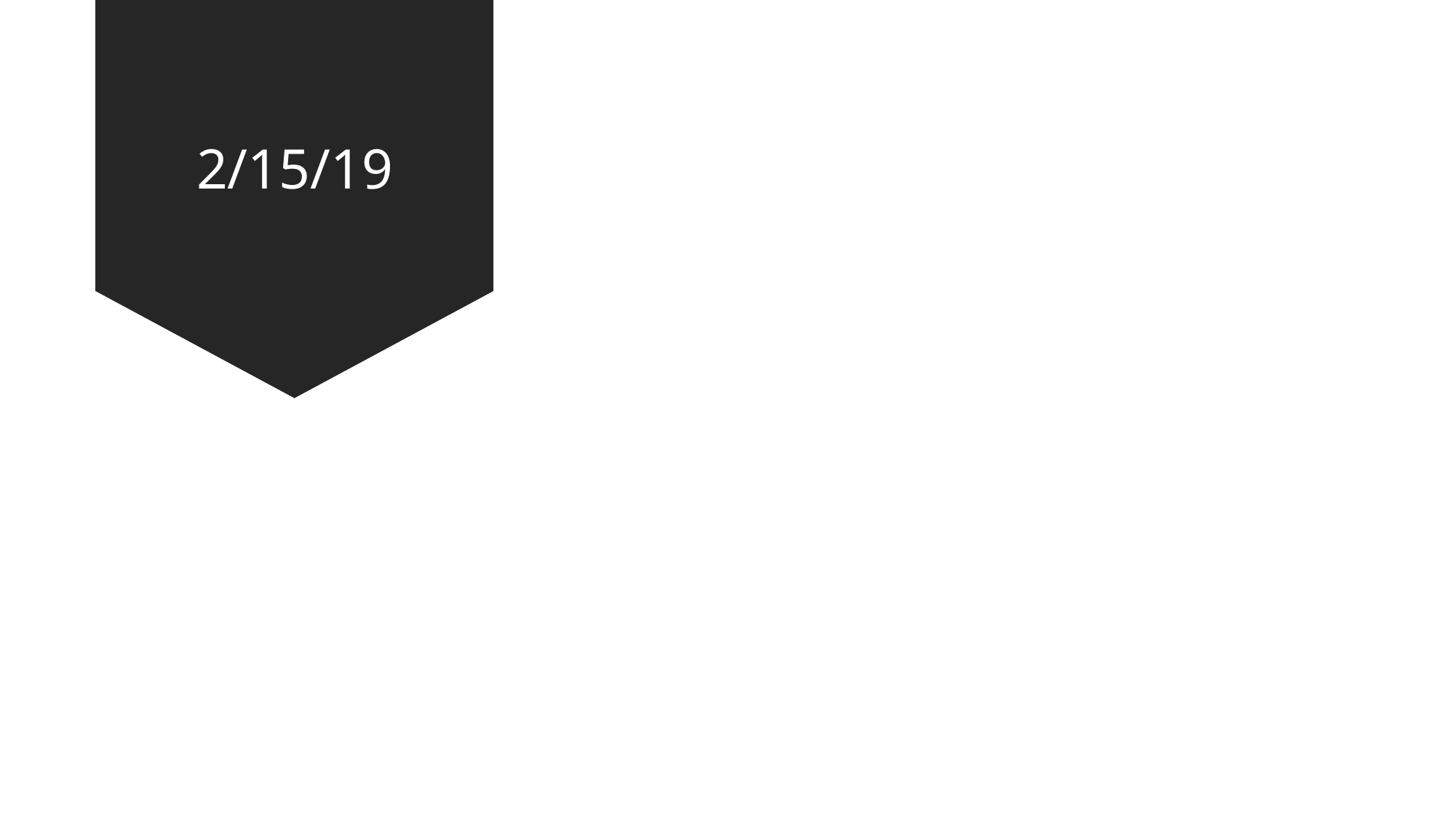

# 2/15/19

## Slide 48
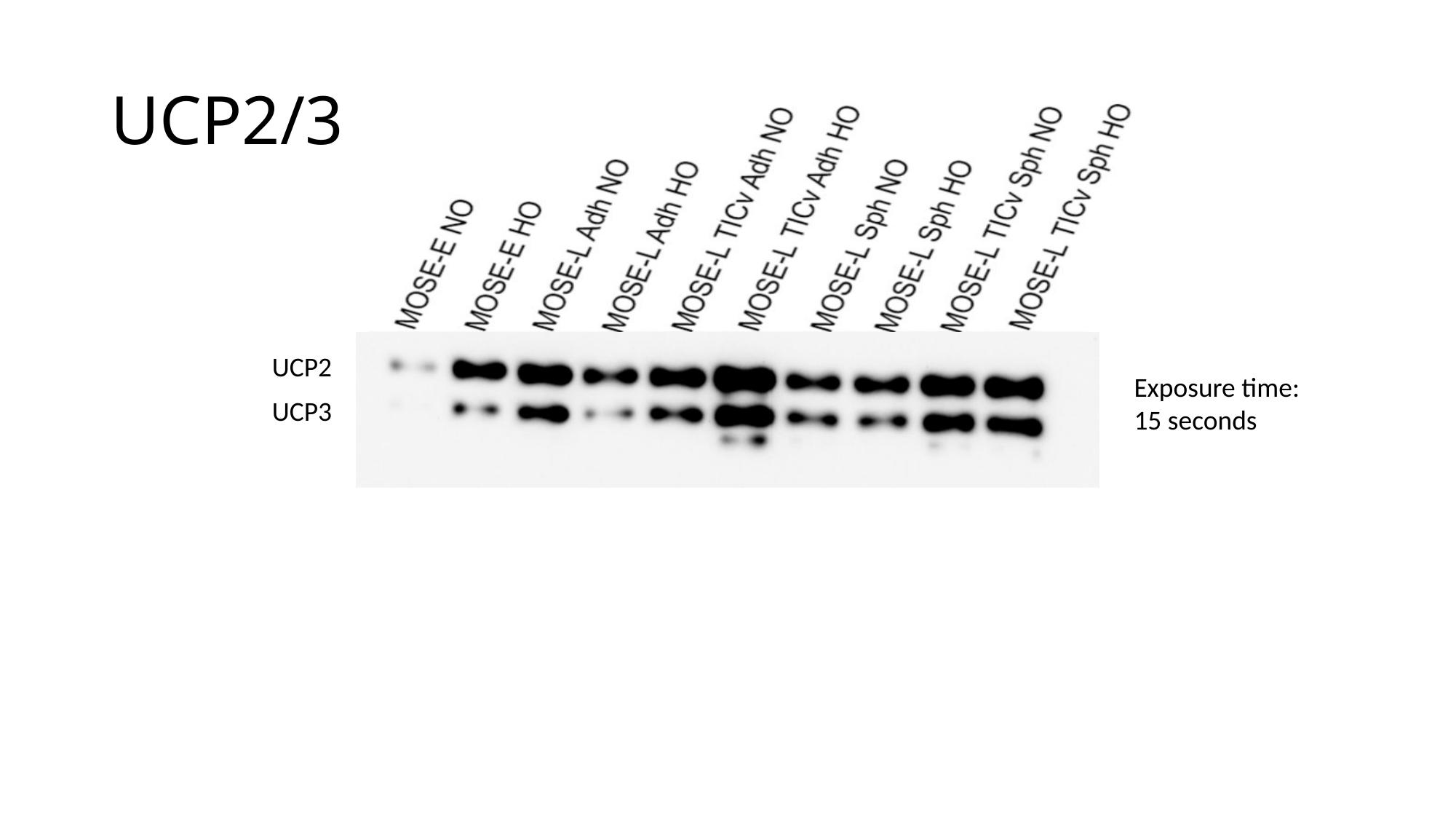

# UCP2/3
UCP2
Exposure time: 15 seconds
UCP3

## Slide 49
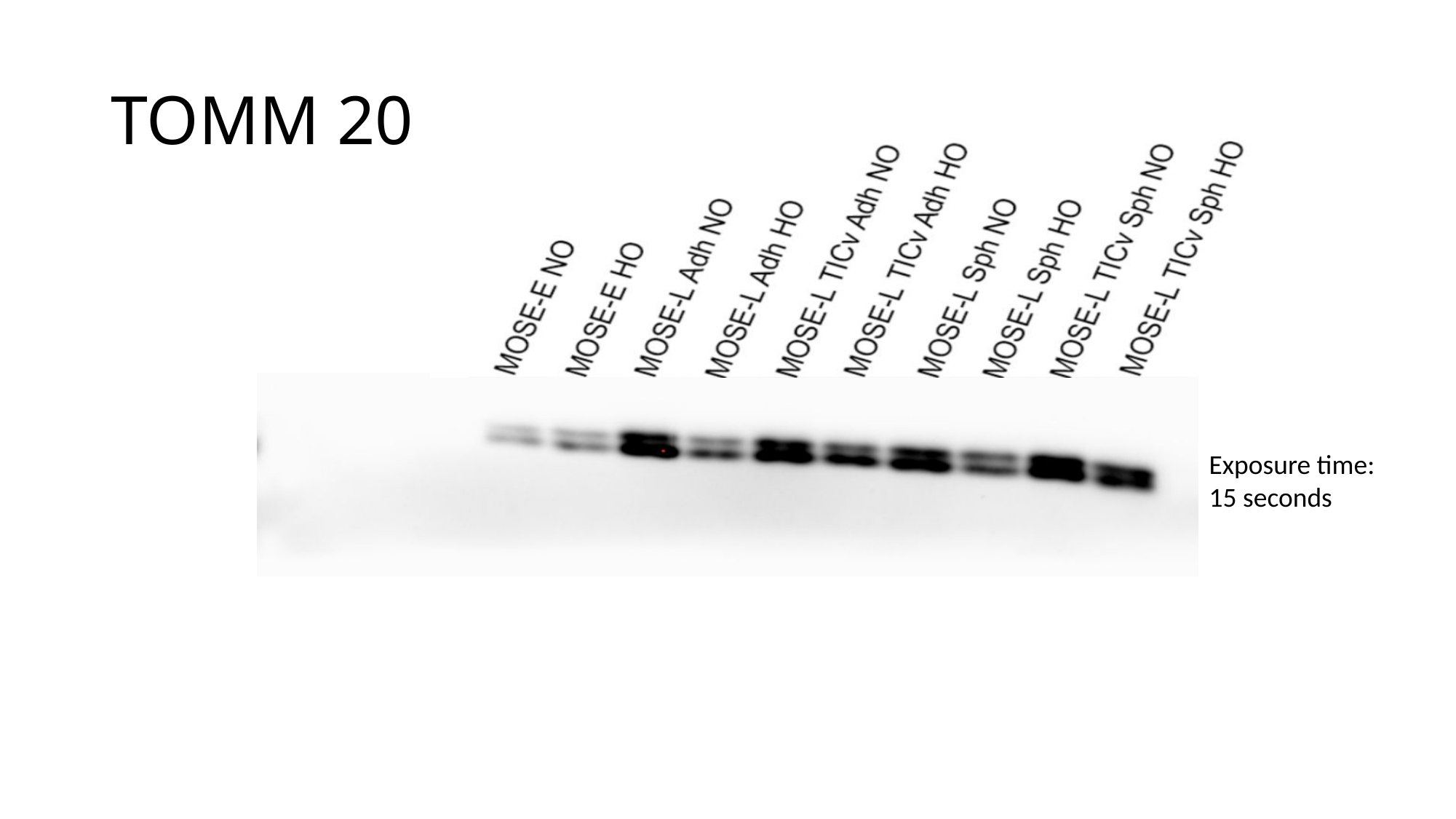

# TOMM 20
Exposure time: 15 seconds

## Slide 50
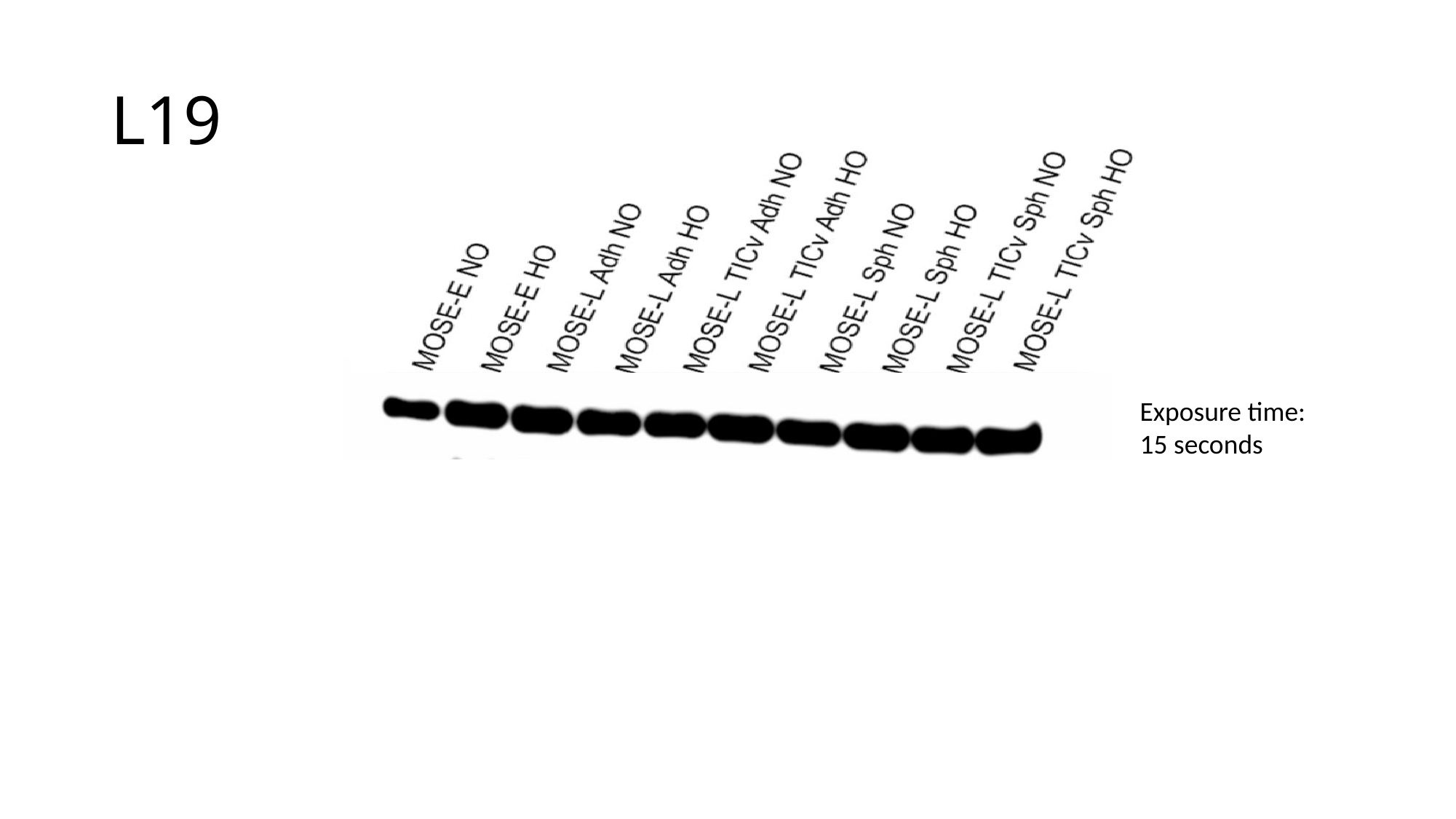

# L19
Exposure time: 15 seconds
